# Bile acid metabolism and signaling in health and disease: molecular mechanisms and therapeutic targets

**DOI:** 10.1038/s41392-024-01811-6

**Published:** 2024-04-26

**Authors:** Joshua S. Fleishman, Sunil Kumar

**Affiliations:** Department of Pharmaceutical Sciences, College of Pharmacy and Health Sciences, St. John’s University, Queens, NY USA

**Keywords:** Structural biology, Physiology, Drug discovery

## Abstract

Bile acids, once considered mere dietary surfactants, now emerge as critical modulators of macronutrient (lipid, carbohydrate, protein) metabolism and the systemic pro-inflammatory/anti-inflammatory balance. Bile acid metabolism and signaling pathways play a crucial role in protecting against, or if aberrant, inducing cardiometabolic, inflammatory, and neoplastic conditions, strongly influencing health and disease. No curative treatment exists for any bile acid influenced disease, while the most promising and well-developed bile acid therapeutic was recently rejected by the FDA. Here, we provide a bottom-up approach on bile acids, mechanistically explaining their biochemistry, physiology, and pharmacology at canonical and non-canonical receptors. Using this mechanistic model of bile acids, we explain how abnormal bile acid physiology drives disease pathogenesis, emphasizing how ceramide synthesis may serve as a unifying pathogenic feature for cardiometabolic diseases. We provide an in-depth summary on pre-existing bile acid receptor modulators, explain their shortcomings, and propose solutions for how they may be remedied. Lastly, we rationalize novel targets for further translational drug discovery and provide future perspectives. Rather than dismissing bile acid therapeutics due to recent setbacks, we believe that there is immense clinical potential and a high likelihood for the future success of bile acid therapeutics.

## Introduction

Globally, cardiometabolic diseases present a substantial health risk, positively correlating with most, if not all of the top CDC-listed causes of American death.^1^ The prevalence of these conditions has increased rapidly from an American morbidity of 37.5% in 2011 to 41.8% in 2018, primarily comprised of type 2 diabetes mellitus (T2DM), obesity, non-alcoholic fatty liver disease (NAFLD), non-alcoholic steatohepatitis (NASH), and atherosclerotic cardiovascular disease (ASCVD).^2,3^ Alongside cardiometabolic diseases, non-metabolic conditions such as inflammatory bowel disease (IBD) and cancer have also been realized as intricate challenges to human health. Despite the apparent heterogeneity, these diseases share the commonality of complex biochemistry, all lack fully deterministic models for disease pathogenesis, and have no well-defined curative treatments. This shared complexity imposes a significant burden on humanity, diminishing quality of life and leading to mortality. Novel treatments are imperative to both better control and potentially cure these diseases.

Bile acids (BAs) are hepatically synthesized cholesterol derivatives that function as amphipathic surfactants and systemic endocrine hormones. Alongside regulating their own synthesis and enterohepatic circulation, BAs are potent modulators of macronutrient (lipid, carbohydrate, protein) metabolism and the systemic pro-inflammatory/anti-inflammatory balance. BAs provide complex physiological modulation by binding to Farnesoid X Receptor (FXR) and Takeda G Protein-Coupled Receptor 5 (TGR5), the canonical BA receptors, while exerting effects at other more recently characterized non-canonical BA receptors.^4,5^ Alterations in BA physiology are directly correlated to the pathogenesis of cardiometabolic, inflammatory, and neoplastic diseases.^6^ Hence, the complex role of BAs on physiology provides ample targets for drug discovery.

The most developed BA therapeutic is the FXR agonist candidate Obeticholic Acid (OCA) by Intercept Pharmaceuticals. Although already approved for primary biliary cholangitis (PBC) at lower doses, it was rejected for NASH indication approval by the FDA due to significant dose-dependent toxicities.^7^ Rather than dismissing BA therapeutics due to recent setbacks, we believe that there is immense clinical potential and a high likelihood for the future success of BA therapeutics. In this review, we provide a bottom-up approach on BAs, mechanistically explaining their biochemistry, physiology, and pharmacology at canonical and non-canonical receptors. Using this mechanistic model of BAs, we explain how abnormal BA physiology drives disease pathogenesis, emphasizing how ceramide synthesis may serve as a unifying pathogenic feature for cardiometabolic diseases. We provide an in-depth summary on pre-existing BA receptor modulators, explaining their shortcomings and theorizing how they be remedied. Lastly, we rationalize novel targets for further translational drug discovery and provide future perspectives.

## BA biochemistry

### Primary BA synthesis

#### Pathway overviews

**Fig. 1:**
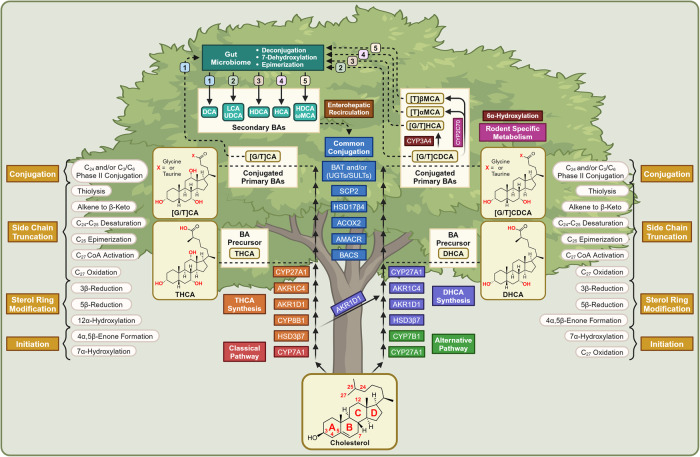
Biochemical synthesis and maturation of BAs. Schematic diagram depicting the biochemical synthesis of BAs. A prefix of G represents a glycine conjugate, while a prefix of T represents a taurine conjugate. Far left and far right labels describe the enzymatic reactions shown parallel within the tree. Top numbering keeps track of which conjugated primary BAs become which secondary BAs. This figure was created with BioRender.com

Hepatic BA synthesis tends to follow a common four-stage pattern: 7α-hydroxylation initiation, sterol ring modification, side chain truncation, and phase II conjugation (Fig. 1).^8^ Each of the previously mentioned metabolic steps takes place in the: endoplasmic reticulum (ER), cytosol, mitochondria, and peroxisome, respectively.^9^ There are two parallel metabolic pathways, the classical pathway and the alternative pathway, which both perform the first three steps of BA synthesis but use the same enzymes for phase II conjugation. ~90% of human BAs and ~75% of mice BAs are products of the classical pathway. The remaining ~10% in humans and ~25% in mice are synthesized via the alternative pathway, otherwise known as the acidic pathway.^10^ Metabolic flux through the alternative pathway has been correlated with the upregulation of Cytochrome P450 Family 7 Subfamily B Member 1 (CYP7B1) expression in response to adaptive physiological responses, such as those in response to liver disease, a high fat (HF)/high cholesterol (HC) diet, or cold exposure.^11–13^

#### The classical pathway of BA synthesis

The classical pathway for BA synthesis begins with 7α-hydroxylation initiation of cholesterol through the pathway’s rate-limiting enzyme Cytochrome P450 Family 7 Subfamily A Member 1 (CYP7A1).^14^ For the first branch of the classical pathway, following initiation, C_3_, C_4_, and C_5_ are oxidized to a 4α,5β-enone by 3-Beta-Hydroxysteroid Dehydrogenase Type 7 (HSD3β7) and are 12α-hydroxylated by Cytochrome P450 Family 8 Subfamily B Member 1 (CYP8B1). Subsequently, the A ring is reduced in the 5β and 3β positions by Aldo-keto Reductase Family 1 Member D1 (AKR1D1) and Aldo-keto Reductase Family 1 Member C4 (AKR1C4), respectively. Lastly, C_27_ is oxidized three times into an alcohol, aldehyde, and eventually a carboxylic acid by Cytochrome P450 Family 27 Subfamily A Member 1 (CYP27A1). This first metabolic branch produces Trihydroxycholestanoic Acid (THCA), the precursor for the BA Cholic Acid (CA). In comparison, after CYP7A1 metabolism, BAs may partake in a second metabolic branch that skips 12α-hydroxylation by CYP8B1. Instead, 7α-hydroxycholesterol is metabolized by HSD3β7, AKR1D1, AKR1C4, and CYP27A1 to produce Dihydroxycholestanoic Acid (DHCA), the precursor for the BA Chenodeoxycholic Acid (CDCA).^15^

#### The alternative pathway of BA synthesis

The alternative pathway for BA synthesis begins with C_27_ oxidation of cholesterol to a spectrum of alcohol, aldehyde, and carboxylic acid metabolites by CYP27A1. Subsequently, the alternative pathway 7α-hydroxylates with CYP7B1, the alternative pathway analogous enzyme to CYP7A1.^14^ The remainder of the alternative pathway is identical only to the DHCA-synthetic branch of the classical pathway: HSD3β7, AKR1D1, AKR1C4, and CYP27A1.^14,15^ However, the final CYP27A1 mediated step for the alternative pathway is different compared to the classical pathway, in which only one or two oxidations are performed to complete the oxidation of C_27_ into a carboxylic acid.^14^

#### Convergent synthesis—BA conjugation

Convergently, both the classical and alternative pathways meet at the common conjugation phase of synthesis. The carboxylates of THCA and DHCA are activated to form thioesters with Coenzyme-A (CoA) by BA-CoA Synthase (BACS) and are C_25_ epimerized from R to S stereochemistry by α-Methylacyl-CoA Racemase (AMACR). The thioester is α,β desaturated at C_24_ and C_25_ by Acyl-CoA Oxidase 2 (ACOX2), is oxidized to a β-keto thioester by D-Bifunctional Protein (HSD17β4), and is thiolysed by Peroxisomal Thiolase 2 (SCP2) to release propionyl-CoA.^15^ These last three reactions heavily mimic the β-oxidation of odd-numbered fatty acids: desaturation, β-keto thioester formation, and thiolysis. The resulting CA-CoA and CDCA-CoA are then C_24_ phase II conjugated by BA-CoA:amino acid N-acetyltransferase (BAT) to produce C_24_ glycine or taurine conjugated BAs in humans or only taurine conjugates in rodents.^16^ In small quantities sulfation at C_3_ or C_6_ by Sulfotransferase Family 2 A Member 1 (SULT2A1) or Family 2B Member 8 (SULT2B8) may occur. In addition, small quantities of glucuronidated metabolites at C_3_, C_6_, or C_24_ by UDP-Glucuronosyltransferase Family 1 Member A3 (UGT1A3), Family 2 Member B4 (UGT2B4), or Family 2 Member B7 (UGT2B7) may occur.^17,18^ Products synthesized after 7α-hydroxylation initiation, sterol ring modification, side chain truncation, and phase II conjugation are denoted as primary conjugated BAs: glycine/taurine conjugates of CA and CDCA in the classical pathway and only CDCA in the alternative pathway.^19^ CDCA may be further metabolized by Cytochrome P450 Family 3 Subfamily A Member 4 (CYP3A4) to produce Hyocholic Acid (HCA), alternatively known as γ-Muricholic Acid (γMCA), in trace quantities in humans and substantial quantities in rodents. Only rodents metabolize CDCA with Cytochrome P450 Family 2 Subfamily C Member 70 (CYP2C70) to produce α-Muricholic Acid (αMCA), an intermediate whose 7α-alcohol is further epimerized by CYP2C70 to 7β to produce β-Muricholic Acid (βMCA).^20–23^

### Microbial maturation of primary BAs to secondary BAs

#### A brief overview of BA maturation

**Fig. 2:**
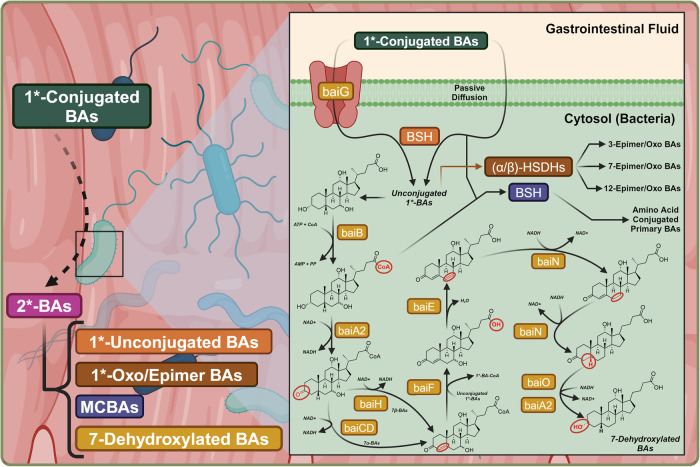
Microbial maturation of primary BAs to secondary BAs. Schematic diagram depicting the gut maturation of primary conjugated BAs. 1* and 2* are used as shorthand notation to represent primary and secondary, respectively. NAD+ and NADH are the biochemical cofactors Nicotinamide Adenine Dinucleotide (NAD+) and Nicotinamide Adenine Dinucleotide Hydride (NADH), respectively. Red circled regions and atoms highlight the motifs changed by the prior performed metabolic reaction. This figure was created with BioRender.com

Enterohepatic circulation and gut exposure provide ample opportunities for BA modification and diversification (Fig. 2). The gut microbiome can modify primary BAs by deconjugation with Bile Salt Hydrolase (BSH), 7α/β-dehydroxylation with proteins of the *bai* operon, and epimerization by the family of Hydroxysteroid Dehydrogenases (HSDHs) to produce what are known as secondary BAs.^24^ Once systemically reabsorbed, a topic developed in further sections, secondary and primary unconjugated BAs may be reconjugated. Absent in humans, mice express Cytochrome P450 Family 2 Subfamily A Member 12 (CYP2A12) and are able to 7α-rehydroxylate 7α/β-dehydroxylated secondary BAs.^25^ In humans, the main secondary BAs are Deoxycholic Acid (DCA), Lithocholic Acid (LCA), and Ursodeoxycholic Acid (UDCA), while Hyodeoxycholic Acid (HDCA) is found in trace quantities.^19^ Similar to humans, rodents synthesize DCA, LCA, but generate larger quantities of UDCA. Rodents additionally metabolize αMCA and βMCA to create substantial quantities of HDCA and ω-Muricholic Acid (ωMCA).^26^

#### Deep-dive, BSH - BA deconjugation & microbially conjugated BAs

BSH is a N-terminal nucleophilic hydrolase that uses a N-terminal cysteine residue to deconjugate primary BAs, commonly thought to serve as gatekeeper reaction for further BA maturation.^27,28^ Control over BA deconjugation via BSH prevalence is important since the conversion of CA/CDCA conjugates into their free form greatly enhances their antimicrobial nature, restricting *Clostridium difficile* growth.^29,30^ BSH is found across most major phyla such as gram-positive *Bifidobacterium*, *Lactobacillus*, *Clostridium*, *Enterococcus*, *Listeria*, and gram-negative *Stenotrophomonas*, *Brucella*, and *Bacteroides*, which is suggested to play a substantial role in total deconjugation.^31–39^ This widespread prevalence of BSH suggests that *BSH* may be horizontally transferable.^28,40,41^

Contrary to original belief, BA conjugation is not a process that is exclusive to the liver. In addition to deconjugating glycine/taurine conjugates, microbial BSH may also conjugate microbially synthesized (CA/CDCA/DCA/UDCA)-CoA to other amino acids in a microbiota-dependent manner. In addition, BSH may catalyze trans-amidation reactions, where glycine/taurine C_24_ motifs are exchanged for that of other amino acids.^42^ All proteinaceous amino acid conjugates have been found except those containing aspartate or proline. This process has been identified in *Enterocloster bolteae*, *Clostridium perfringens*, and the resulting molecules are referred to as microbially conjugated BAs (MCBAs).^28,43,44^

In comparison to the role of glycine/taurine conjugates, MCBAs contain varied lipophilicities and carry modified steric bulk, modulating BA physiochemical and pharmacological properties.^45^ The more hydrophobic the MCBA, the more potent it acts as an antimicrobial agent, perhaps acting as a regulatory feedback loop against MCBA synthesis.^43^ MCBAs have already been characterized to both increase and decrease the pharmacological properties of canonical BAs at nuclear transcription factor receptors.^44^ The differential pharmacological activity of MCBAs compared to classical BAs is strongly species dependent. Comparing humans and mice, some MCBAs provide similar pharmacological effects relative to canonical BAs, while others provide entirely different or neutral effects.^44^ Microbial conjugates of CA have been found to retain FXR agonism and are enriched in IBD and cystic fibrosis patients, although it is unknown if it is causative or correlative. More research is needed to see how this new form of conjugation modifies the properties of other BAs and impacts disease pathogenesis.

##### Deep-dive, 7α/β-dehydroxylation and the *bai* operon

7α/β-dehydroxylation of BAs has been strongly associated with the *Clostridium* family of bacteria, including *C. scindens*, *C. hylemonae*, *C. hiranonis*, and *C. sordellii*, encoded by the 9 gene *bai* operon.^46–50^ Through the process of 7α-dehydroxylation, CA is converted into DCA and CDCA/UDCA are converted into LCA. C_7_ hydroxylated BAs such as CA and CDCA/UDCA are transported into bacteria by baiG, and from such are C_24_ conjugated into a thioester with CoA by baiB.^51^ BaiA2 then oxidizes the C_3_ alcohol to form a ketone. BaiCD oxidizes C_4_/C_5_ of 7α-hydroxy BAs, while baiH performs the same for 7β-hydroxy BAs, both of which creating 4α,5β-enones, similar in outcome to the reaction performed by HSD3B7 in primary BA synthesis.^50,52,53^ The enzyme baiF then performs a trans-thioesterification, in which the CoA of the oxidized 7-hydroxy metabolite is transferred to a new substrate BA.^46^

The rate limiting step of this pathway is performed by the 7-dehydratase baiE, which although not specific for CoA conjugation, is enzymatically more efficient with CoA conjugates.^53^ Catalytically a D35/H83 catalytic dyad abstracts the 6α-H, allowing for electron delocalization within the adjacent conjugated enone π-system. Through a suspected E1cb mechanism, the 7-hydroxyl (OH) is eliminated as a leaving group which retrieves the proton originally abstracted by H83 and exits the enzyme as water, resulting in the formation of a 4α,5β,6γ,7δ-enone.^54^ Two steps of reduction are performed by baiN to convert the substrate from a 4α,5β,6γ,7δ-enone to a 4α,5β-enone and to a 3-oxo-BA, while baiA2/baiO performs the final step in reducing C_3_ back to an alcohol.^55,56^

##### Bacteria conserve nine genes to perform BA 7α/β-dehydroxylation

For what reason do bacteria conserve the entire *bai* operon if it is just baiE/baiN that perform the net reaction? In other words, what value do the other genes add to the operon that cannot be found purely with baiE/baiN? BaiB likely serves as a commitment enzyme analogous to that of thiokinase in the process of β-oxidation. In this case, baiB likely provides the bacteria with a way to regulate pathway activity besides that intrinsic to operon transcriptional regulation. BaiF likely serves multiple valuable functions. In situations of high bacterial BA uptake, it may enhance the steady-state rate at which BAs are committed to the pathway. If baiB does exhibit product inhibition or post-translational regulation, baiF may allow the pathway to continue at a basal rate regardless of baiB activity. Lastly, baiF by performing a trans-thioesterification is energetically thrifty, preventing the bacterial expenditure of more ATP for CoA ligation.

In metabolism it is uncommon if not unseen to see direct deoxygenation of a monofunctional alcohol to an alkane. The rest of the *bai* operon proves its value by breaking this single complex problem down to multiple standard biochemical reactions. BaiA2 oxidizes the C_3_ alcohol to a ketone to enhance its electron-withdrawing power, using the standard reversible biochemistry of alcohol to ketone oxidations (Ex: Lactate Dehydrogenase). This new ketone increases the ease at which baiCD/baiH is able to further oxidize, resembling the reverse reaction of enzymes commonly associated with steroid metabolism (Ex: 5α-Reductase). BaiE dehydrates and baiN reduces in close proximity to the baiA2-baiCD/baiH installed electron-withdrawing enone, very similar to fatty acid synthesis (Ex: Fatty Acid Synthase). BaiO/baiA2 finishes the pathway by reversing the initial alcohol to ketone oxidation. At first glance, this pathway seems redundant, but upon further observation it is incredible to see how metabolism repurposes various standard archetypal reactions to obtain new outcomes.

#### Deep-dive, HSDH epimerization

BAs may be oxidized and epimerized by a two-step reaction, in which the existing alcohol is oxidized to a ketone by the removal of a hydride ion, followed by reduction of the ketone on the opposite face. These two reactions are enzymatically performed by different position-specific HSDHs, likely not originating from the same species of bacteria.^57^ BAs may be oxidized at the 3, 7, or 12 positions to generate oxo-intermediates and are then reduced to form 3, 7, or 12 epimers. 3-dehydrogenation is associated with *Blautia producta*, *Eggerthella* genus, *Enterorhabdus mucosicola*, and *Acinetobacter lwoffii*.^56,58–61^ *Eggerthella lenta* 3α-HSDH is a rare exception that is able to metabolize BAs in their conjugate form, challenging the general idea that BSH metabolism is necessary for further maturation into a secondary BA.^62^ 7α-dehydrogenation is confirmed in *Clostridium baratii*, while 7β-dehydrogenation is found in *Ruminococcus gnavus*, *Clostridium absonum*, *Stenotrophomonas maltophilia*, and *Collinsella aerofaciens*.^63–67^ 12α-dehydrogenation has been found in *Eggerthella lenta*, *Enterorhabdus mucosicola*, *Clostridium scindens*, *Peptacetopacter hiranonis*, *Clostridium hylemonae*, and *Bacteroides*.^41,56,60,61,68^ 12β-dehydrogenation has been found in *Clostridium paraputrificium*, *Clostridium tertium*, and *Clostridium difficile*.^69^ Through the process of epimerization, CA may be C_7_ epimerized to Ursocholic acid (UCA), C_3_ epimerized to isoCA, or C_12_ epimerized to Epicholic acid (ECA). CDCA may be C_7_ epimerized to form UDCA or C_3_ epimerized to form isoCDCA, while LCA is C_3_ epimerized to isoLCA.

### BA pool composition: structure/functionality

#### Structural diversity provides varying degrees of hydrophobicity

**Fig. 3:**
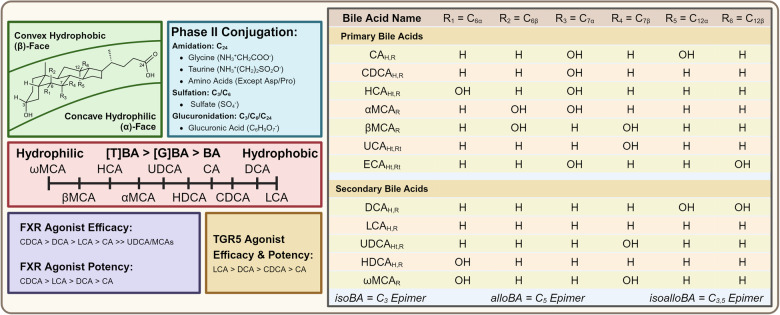
Structural, physiochemical, and pharmacological properties of BAs: Representation of the structural scaffold, conjugates, physiochemical properties, and ranking of endogenous agonist pharmacology at the canonical BA receptors. Sites R_1_ through R_6_ represent locations for enzymatic hydroxylation. α-orientation groups are located beneath the steroidal plane, while β-orientation hydroxyl groups are located above the steroidal plane. Subscripted values next to BA names represent the species of origin: (H = Human), (R = Rodent), (Ht = Human Traces), (Rt = Rodent Traces). This figure was created with BioRender.com

The tetracyclic fused steroidal backbone of BAs has both a concave hydrophilic α-face and a hydrophobic convex β-face, in which hydrophilicity is correlated to both the number and positioning of OH groups on C_6_, C_7_, and C_12_ (Fig. 3). Experimentation with hydrophilic-hydrophobic indices has revealed that the addition of a single β-OH group to an already existing poly-α-hydroxylated scaffold can greatly increase hydrophilicity. The addition of polar surface area to the hydrophobic β-face disrupts pure hydrophobic interactions, resulting in a BA that is less amphipathic and more hydrophilic. The relative physiochemical hydrophilicities of unconjugated free BAs are as follows: ωMCA > βMCA > HCA > αMCA > UDCA > HDCA > CA > CDCA > DCA > LCA. Relative rankings remain the same regardless of the type of conjugation. As a whole, the hydrophilicities of BA conjugation types are as follows: taurine conjugates > glycine conjugates > free BAs.^70^

#### Individual BAs work together to produce the BA pool

The BA pool is defined as all the BAs within enterohepatic circulation, disregarding those in systemic circulation. Breaking this down by tissue, ~85–90% of BAs are in the gut, 10–15% in the gallbladder, and 1% in the liver.^71^ As a guiding principle, BAs found in the liver are strongly conjugated. Those within the biliary tree, small intestine, and systemic circulation are moderately conjugated, while large fractions of free BAs are found in the large intestine and feces.

BA pool composition is defined as the following four metrics: percent occurrence per BA, percent 12α-hydroxylated, percent glycine/taurine conjugated, and percent primary BAs vs. secondary BAs. The BA pool composition of different biological tissues/fluids is frequently measured by liquid chromatography-mass spectrometry analysis (Table 1). The human hepatic BA pool is mostly comprised of CA and CDCA. Once exposed to the gut and excreted as feces, the human BA pool composition shifts strongly more towards the hydrophobic BAs DCA and LCA. The human serum BA composition, derived from hepatic excretion and gut BA reabsorption, is mostly comprised of CA, CDCA, DCA, and UDCA.

**Table 1:** Interspecies variation in BA pool composition: human vs. mice

	Human liver (%) (*n* = 10)^723^	Mice liver (%) (*n* = 20)^724^	Human feces (%) (*n* = 97)^725^	Mice colon (%) (*n* = 8)^726^	Mice ileum (%) (*n* = 11–12)^727^	Human serum (%) (*n* = 56)^511^	Mice serum (%) (*n* = 9)^724^	Human plasma (%) (*n* = 18)^724^	Mice plasma (%) (*n* = 6)^724^	Human adipose tissue (%) (*n* = 24)^726^	Mice gallbladder (%) (*n* = 8)^726^
*Primary BAs*											
CA	Not Measured	0.3	5.16	6.12	7.39	7.18	0.7	3.85	2.45	0	1.16
[G]CA	Not Measured	0.21	18.32	0.12	Not Measured	7.53	0.2	7.27	0.13	8.83	0.18
[T]CA	Not Measured	36.85	0.38	8.31	7.53	1.78	39.4	1.99	41.26	14.72	42.63
CDCA	Not Measured	0.02	2.72	0.44	0.23	14.08	0.04	8.98	0.2	0	0.01
[G]CDCA	Not Measured	0	Not Measured	0.18	Not Measured	23.28	0.01	32.2	0.03	18.73	0.02
[T]CDCA	Not Measured	1.66	0.31	0.42	0.18	3.43	2.69	7.12	1.52	3.06	0.68
HCA/γMCA	Not Measured	0	0.62	0.16	Not Measured	0	0	0	0	0	0
[G]HCA	Not Measured	Not Measured	Not Measured	0	Not Measured	Not Measured	Not Measured	Not Measured	Not Measured	0	0
[T]HCA	Not Measured	0	Not Measured	0.23	Not Measured	Not Measured	Not Measured	Not Measured	Not Measured	0	0.3
αMCA	Not Measured	0.11	Not Measured	0.38	0.97	0	0.13	0	0.2	0	0.04
[T]αMCA	Not Measured	7.86	Not Measured	3.06	4.27	Not Measured	11.07	0	7.84	0	9.79
βMCA	Not Measured	1.27	Not Measured	1.46	3.69	0	0.69	0	2.09	0	0.08
[T]βMCA	Not Measured	32.59	Not Measured	8.95	18.67	Not Measured	24.18	0	29.91	5.65	20.46
*Secondary BAs*											
DCA	Not Measured	0.02	0	32.29	0.21	17.53	0.54	12.97	0.96	11.07	0.01
[G]DCA	Not Measured	0	10.15	0.11	Not Measured	10.38	0	10.12	0	17.55	0.01
[T]DCA	Not Measured	1.78	0.44	4.89	0.15	1.53	2.05	2.71	1.84	4.95	1.43
LCA	Not Measured	0	50.43	2.01	0	0.78	0	0	0	0	0.01
[G]LCA	Not Measured	0	0.02	0.3	Not Measured	0.63	0	1.28	0	9.54	0.01
[T]LCA	Not Measured	0.11	0.09	0.24	0	Not Measured	0.04	0.13	0.09	0	0.15
UDCA	Not Measured	0.01	0	0.59	0.27	3.68	0.02	0.14	0.66	0	0
[G]UDCA	Not Measured	0	0.07	0.02	Not Measured	8.25	0.01	9.26	0.01	2.36	0.01
[T]UDCA	Not Measured	2.75	Not Measured	0.45	0.73	Not Measured	3.24	0.85	2.22	0.94	4.21
HDCA	Not Measured	0.01	8.32	10.41	Not Measured	0	0	0	0.33	0	0
[G]HDCA	Not Measured	Not Measured	0.07	0.02	Not Measured	0	Not Measured	Not Measured	Not Measured	0.24	0.01
[T]HDCA	Not Measured	0.01	Not Measured	2.05	Not Measured	Not Measured	Not Measured	Not Measured	Not Measured	0	1.84
ωMCA	Not Measured	0.21	Not Measured	1.23	2.92	0	0.51	0	0.67	0	0.04
[T]ωMCA	Not Measured	13.52	Not Measured	14.16	52.78	Not Measured	13.09	0	6.54	2.36	16.56
*Special BA Derivatives*											
alloCA	Not Measured	0.04	Not Measured	Not Measured	Not Measured	Not Measured	0.18	0	0.14	Not Measured	Not Measured
7-oxo-DCA	Not Measured	0.11	Not Measured	0.96	Not Measured	Not Measured	0.54	0	0.51	0	0.35
alloDCA	Not Measured	0	Not Measured	Not Measured	Not Measured	Not Measured	0.07	0.01	0.08	Not Measured	Not Measured
Allo-3β-DCA	Not Measured	0	Not Measured	Not Measured	Not Measured	Not Measured	0	0.85	0	Not Measured	Not Measured
Allo-12β-DCA	Not Measured	0	Not Measured	Not Measured	Not Measured	Not Measured	0	0	0	Not Measured	Not Measured
Allo-3β-LCA	Not Measured	0	Not Measured	Not Measured	Not Measured	Not Measured	0	0	0	Not Measured	Not Measured
alloLCA	Not Measured	0	Not Measured	Not Measured	Not Measured	Not Measured	0	0	0	Not Measured	Not Measured
3-oxo-LCA	Not Measured	0	Not Measured	Not Measured	Not Measured	Not Measured	0	0	0	Not Measured	Not Measured
7-oxo-LCA	Not Measured	0	Not Measured	Not Measured	Not Measured	Not Measured	0	0.07	0.06	Not Measured	Not Measured
[T]7-oxo-LCA	Not Measured	0.53	Not Measured	Not Measured	Not Measured	Not Measured	0.59	0	0.29	Not Measured	Not Measured
12-oxo-LCA	Not Measured	0	Not Measured	0.41	Not Measured	Not Measured	0.01	0.2	0	0	0
6,7-dioxo-LCA	Not Measured	0.03	Not Measured	0	Not Measured	Not Measured	0	0	0	Not Measured	Not Measured
isoLCA	Not Measured	Not Measured	2.9	Not Measured	Not Measured	0	Not Measured	Not Measured	Not Measured	Not Measured	Not Measured
7-oxo-HDCA	Not Measured	Not Measured	Not Measured	0.02	Not Measured	Not Measured	Not Measured	Not Measured	Not Measured	0	0
*Categorical Breakdown*											
Total CA	47.2	37.4	23.86	14.55	14.92	16.49	40.48	13.11	43.97	23.56	43.97
Total CDCA	46.27	1.68	3.03	1.04	0.41	40.79	2.74	48.3	1.75	21.79	0.71
Total DCA	3.11	1.91	10.59	38.24	0.36	29.44	3.19	26.65	3.39	33.57	1.8
Total LCA	1.09	0.67	53.44	2.96	0	1.41	0.65	1.69	0.44	9.54	0.17
Total UDCA	2.33	2.76	0.07	1.06	1	11.93	3.27	10.26	2.89	3.3	4.22
Total MCA	Not Measured	55.58	Not Measured	42.13	83.3	Not Measured	49.67	0	47.58	8.25	49.12
Glycine Conjugates	Not Measured	0.21	28.63	0.75	0	50.07	0.22	60.13	0.17	57.25	0.24
Taurine Conjugates	Not Measured	97.87	19.54	42.88	84.31	14.27	96.55	20.07	91.64	40.51	98.23
Non-Conjugated	Not Measured	1.92	51.83	56.37	15.69	35.66	3.23	19.8	8.19	2.24	1.53

Original data was obtained and passed through a softmax function, alternatively known as a Boltzman distribution to produce the shown percentage distributions of tissue BA pool composition. BAs not measured within individual studies are listed as Not Measured

It is incredibly important to note that tissue-specific BA pool composition is highly species-dependent. To contrast the BA pool of humans with rodents such as mice, an overwhelming proportion of the mice BA pool in all tissues is composed of the highly polar MCA family. This is imperative to note since as we shall discuss the MCA family of BAs are potent FXR antagonists. Conjugation of human BAs mostly favors glycine conjugation, while in rodents is exclusive to taurine conjugation. Both in MCA prevalence and conjugation preference, interspecies differences between human and rodent BA pools make it incredibly challenging to translate research from rodent models to human applications. From such it is evident that wild-type mice models do not perfectly replicate the properties of the human BA pool.^72^

#### A balanced BA pool is essential for gut lipid absorption

The specific physiochemical properties and amphipathic nature of BAs allows for them to act as digestive surfactants, assisting in the solubilization and absorption of lipid nutrients and lipid-soluble vitamins (A, D, E, K). Deviations from a normal BA pool composition by either impaired synthetic or impaired enterohepatic circulation mechanisms will result in an impaired ability to absorb adequate nutrition and have normal growth patterns.^14,73,74^ Phase II conjugation with glycine or taurine greatly increases the acidity of BA carboxylate groups, lowering their unconjugated pKa from ~5 to ~4 in the case of glycine conjugates and ~1 in the case of taurine conjugates.^75^ The lower the average pKa of the BA pool, the higher the overall ratio of salt to protonated acid. This effectively allows for the biochemical optimization of BA pool composition to obtain physiological pH solubilization of fat-soluble nutrients by assisting in micelle formation.^76^ BA pool compositions that are too hydrophobic are hepatotoxic, causative for cholestasis, and are poor surfactants. In comparison, an overly hydrophilic composition cannot stabilize BA-nutrient micelles.^77^ Therefore, the maintenance of proper species-specific BA pool composition is essential to ensure the optimal surfactant-mediated absorption of lipid-nutrients.

#### BA pool composition drives balanced pharmacological profiles

Alongside surfactant properties, different BAs have varying pharmacological profiles at the two canonical BA receptors FXR and TGR5. The efficacy and potency rankings for common human BAs as FXR agonists are: CDCA > DCA > LCA > CA >> UDCA/MCAs, and CDCA > LCA > DCA > CA, respectively.^78–81^ The efficacy of UDCA and all MCAs are so low that functionally in the presence of other more potent BAs they act as FXR partial-agonists or antagonists.^82,83^ The analogous rankings for efficacy and potency are identical for TGR5 and is: LCA > DCA > CDCA > CA.^84,85^ Disregarding trace quantities, within the human BA pool CDCA represents the most potent/efficacious FXR agonist, while DCA and LCA are the most potent/efficacious TGR5 agonists. Both most potent/efficacious TGR5 agonists are secondary BAs, highlighting the importance of BA maturation in modulating TGR5 agonism. In healthy human physiology the BA pool consists of more CDCA than DCA/LCA. Accordingly, any factor that manipulates the relative ratio between classical and alternative pathway metabolic flux, or gut conversion of CA to DCA or CDCA to LCA is key in influencing BA pool composition and hence the BA pool agonist profile. Increasing metabolic flux through the alternative pathway by inhibiting or transcriptionally repressing the classical pathway gatekeeper enzymes CYP7A1 or CYP8B1 will produce a BA pool that has higher FXR agonism and decreased TGR5 agonism.^10,86^

## BA physiology

### Mechanisms of enterohepatic circulation & maturation of BAs

**Fig. 4:**
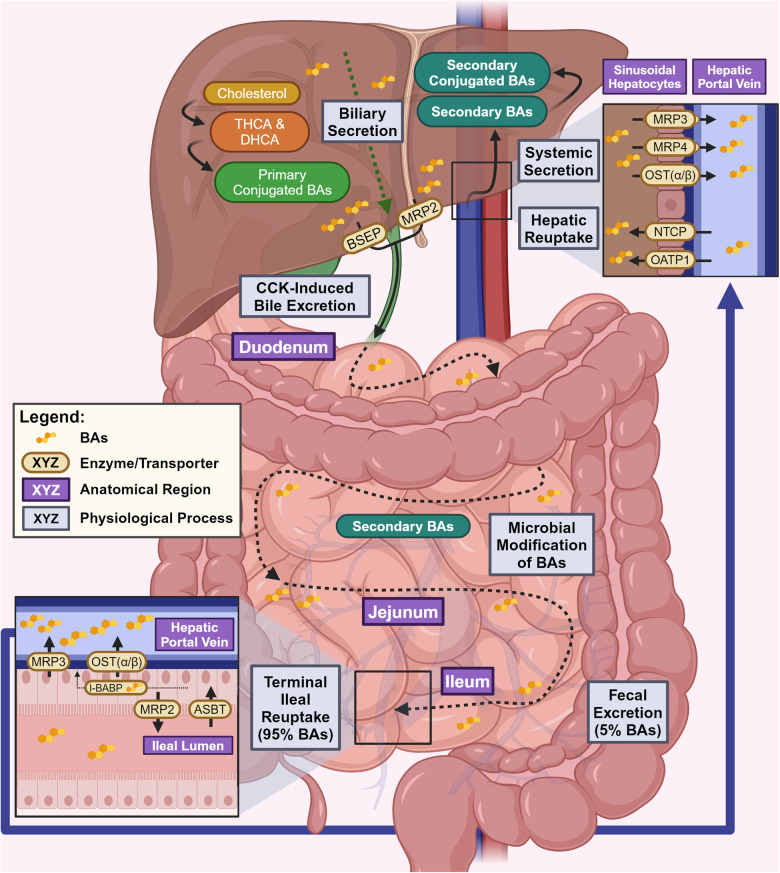
Physiological mechanisms behind BA enterohepatic circulation, maturation, and fecal excretion: Schematic diagram representation of the physiology behind BA enterohepatic circulation, maturation, and fecal excretion. BAs are synthesized in the liver and are excreted into the duodenum for maturation. Matured BAs may either be systemically reabsorbed or excreted in feces. This figure was created with BioRender.com

The steady-state BA pool composition is strongly influenced by the tightly regulated physiological processes of enterohepatic circulation, maturation, and fecal excretion of BAs (Fig. 4). Conjugated BAs are secreted from the basolateral surface of hepatocytes into the bile canaliculi for storage in the gallbladder.^87^ Glycine and taurine conjugated BAs are secreted by Bile Salt Export Pump (BSEP), while sulfate and glucuronate conjugated BAs are secreted by Multidrug Resistance-Associated Protein 2 (MRP2).^88^ The post-prandial release of Cholecystokinin (CCK) contracts the gallbladder, leading to the release of bile into the duodenum.^89^ Within the duodenum, BAs assist in the previously mentioned solubilization of fat-soluble nutrients and mature into secondary BAs.^88,90^ Approximately 95% of intestinal BAs are reabsorbed into the hepatic portal vein due to terminal ileal reuptake by Apical Sodium-Dependent BA Transporter (ASBT) and passive diffusion.^91,92^ Residual BAs that escape ileal reuptake can be excreted in feces, establishing the only known route for cholesterol excretion.^93^

Once within terminal ileal enterocytes, the cytosolic Ileal BA Binding Protein (I-BABP) chaperones BAs from the apical to basolateral membrane, where Organic Solute Transporter α/β (OST (α/β)) and Multidrug Resistance-Associated Protein 3 (MRP3) allow for BA entry into hepatic portal circulation.^94,95^ In contrast, enteric apical MRP2 effluxes BAs back into the ileal lumen. From the hepatic portal vein, conjugated BAs enter sinusoidal hepatocytes by the use of apical Sodium-Taurocholate co-Co-transporting Polypeptide (NTCP), while free BAs enter by use of apical Organic Anion-Transporting Polypeptide 1 (OATP1), both of which complete the cycle of enterohepatic circulation.^75,90^ In situations of hepatic BA overload, the liver can efflux BAs into the systemic circulation via basolateral MRP3, Multidrug Resistance-Associated Protein 4 (MRP4), or OST (α/β).^10^ The hepatic suppression of BSEP/MRP2 expression will limit the quantity of primary BAs that reach the gut for maturation. The ileal suppression of ASBT, MRP3, OST (α/β) expression or induction of MRP2 will enhance fecal loss of BAs and prevent the uptake of gut-modified secondary BAs. Lastly, modifications to the microbial composition of the gut may alter which biosynthetic pathways are utilized for BA maturation.

### BA signaling cascades

#### Canonical BA receptor—FXR, a peripheral signal for nutrient density

##### Molecular biology of FXR

**Fig. 5:**
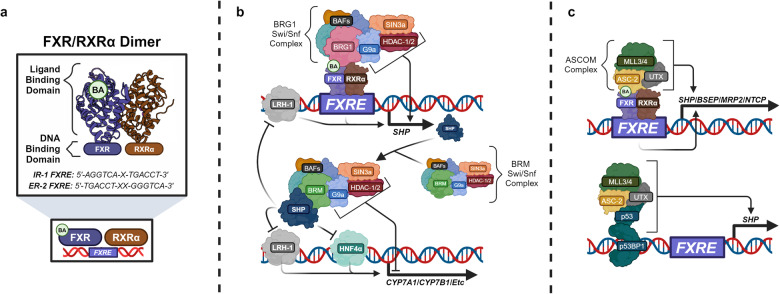
Transcriptional and epigenetic roles of FXR and the FXR-SHP axis. **a** The FXR/RXRα heterodimer binds to either an IR-1 or ER-2 containing FXRE to transcriptionally regulate target gene activity. The FXR/RXRα crystal structure shown is PDB 5Z12.^728^ **b** The FXR-SHP axis inhibits LRH-1/HNF4α-driven gene expression. SHP directly dimerizes with LRH-1/HNF4α to prevent their transcriptional activity. In addition, SHP recruits Swi/Snf complex-mediated epigenetic repression. LRH-1 inhibition represses *SHP* transcription, forming a negative feedback loop. **c** Epigenetic regulation of FXR target genes by the ASCOM complex. BA agonized FXR/RXRα recruits the ASCOM complex to enhance the expression of FXRE-containing genes. Without FXR agonism, p53 and p53BP1 provide basal *SHP* expression. This figure was created with BioRender.com

FXR is a nuclear transcription factor that is encoded by the gene *FXRα/NR1H4* and is expressed as four separate isoforms (FXRα1-α4). FXR isoforms dimerize with Retinoid X Receptor Alpha (RXRα) and bind to two distinct FXR Response Elements (FXREs), the Inverted Hexamer Spaced by 1 Nucleotide (IR-1) motif (5’-AGGTCA-X-TGACCT-3’) and the Everted Hexamer Repeat Spaced by 2 Nucleotides (ER-2) motif (5’-TGACCT-XX-GGGTCA-3’) to regulate the transcriptional activity of target genes (Fig. 5a).^80,96–98^ The ENSEMBL database identifies 388 sequence orthologues for the FXR receptor, where the sequence homologies of *Mus musculus* and *Rattus norvegicus* compared to the human sequence are 87.3% and 81.64%, respectively.^99^ Due to sequence divergence between species, results from animal models may not directly correlate to the human FXR receptor. Cumulative reports from the Protein Atlas database identify the main subcellular location of FXR as within the nucleoplasm. mRNA-seq expression reveals that FXR is expressed minorly in most organs. The following list ranks the top 8 FXR-expressing organs in terms of nTPM (normalized mRNA transcripts per million): liver, small intestine, kidney, adrenal glands, ovary, gallbladder, colon, bladder, pancreas.^100^

##### Epigenetic regulation of *FXR* transcription

Epigenetic methylation has been found to impact FXR activity, in which CpG methylation found in both mouse and human colon cancers directly reduces transcription of *FXR*.^101–103^ 13 sites for CpG methylation have been found in adenomatous polyposis coli deficient mice models.^103^ A clear relationship has been found between *FXR* CpG methylation status and the BA pool comparing healthy pregnant women to those with intrahepatic cholestasis, in which patients with reduced methylation and higher *FXR* transcription were associated with cholestasis, likely due to the repression of trans-biliary bile flux.^104^

##### Post-translational and post-transcriptional regulation of FXR activity

The transcriptional activity of FXR at FXREs is directly reduced by acetylation at K217, in which p300 acetylates and Sirtuin 1 (SIRT1) performs NAD+-dependent deacetylation.^105,106^ SIRT1 further modulates the transcription of *FXR* by activating Hepatocyte Nuclear Factor 1 Alpha (HNF1α).^107^ This regulation is further complicated by the expression of miRNA. miR-34a and miR-22 are both known to silence the expression of SIRT1, hence reducing FXR expression. In addition, FXR exhibits negative autoregulation by inducing the transcription of miR-22.^108–110^ Lastly, FXR has been found to be SUMOylated by Small Ubiquitin-Related Modifier 1 (SUMO1) and 2 (SUMO2), inhibiting its ability to trans-activate FXRE-containing genes.^111^

##### Molecular biology of the FXR-SHP axis

The nuclear FXR receptor has four main functions: to signal for incoming nutrient density (lipid, carbohydrate, amino acid), to enforce negative autoregulation of BA synthesis to prevent BA overload, to reduce the extent of enteric cholesterol absorption, and to inhibit inflammatory responses. Direct FXR agonism induces the expression of Small Heterodimer Partner (SHP), a corepressor which without a DNA binding domain dimerizes with transcription factors to directly inhibit them and/or epigenetically repress their target genes.^112^ This is known as the FXR-SHP axis. All FXR isoforms induce SHP expression by binding to a IR-1 motif, while FXRα2 and FXRα4 may additionally bind to a lone ER-2 motif to induce stronger SHP expression.^98^

##### The FXR-SHP axis transcriptionally and epigenetically regulates target genes

There are two major epigenetic transcriptional complexes associated with FXR-SHP axis activity. The first of which are the ATP-dependent Swi/Snf chromatin remodeling complexes (Fig. 5b). Swi/Snf chromatin remodeling complexes require an ATPase such as Probable Global Transcriptional Activator SNF2L2 (BRM) or Transcriptional Activator BRG1 (BRG1), other proteins known as BRM or BRG1 associated factors (BAFs), the Paired Amphipathic Helix Protein Sin3a (Sin3a)/Histone Deacetylase 1/2 (HDAC-1/2) corepressor complex, and the histone methyltransferase G9a to modulate chromatin compaction.^113–115^ In such, BA-activated FXR/RXRα dimers recruit the BRG1 Swi/Snf complex, providing chromatin relaxation and allowing for FXR-stimulated transcription, such as that which occurs with *SHP*. SHP may recruit the BRM Swi/Snf complex, which allows for H3K9/K14 deacetylation, H3K9 methylation, direct inhibition of Liver Receptor Homolog 1 (LRH-1)/Hepatocyte Nuclear Factor 4 Alpha (HNF4α) transcriptional activation, and epigenetic compaction of LRH-1/HNF4α target genes.^113,116,117^

A secondary epigenetic mechanism is associated with FXR activity, known as the Activating Signal Cointegrator-2 (ASC-2) – Containing Coactivator Complex (ASCOM) (Fig. 5c).^118^ ASCOM is additionally composed of either Histone Methyltransferase Mixed-Lineage Leukemia Proteins 3 (MLL3) or 4 (MLL4) histone methyltransferases and the histone demethylase Ubiquitously Transcribed Tetratricopeptide Repeat, X Chromosome (UTX). Respectively, these methylate H3K4 and remove methylation at H3K27, cumulatively increasing transcriptional activity.^119,120^ BA-activated FXR/RXRα dimers recruit the ASCOM complex to enhance the transcription of *SHP*, *BSEP*, *MRP2*, and *NTCP*.^120,121^ The ASCOM complex has an additional role with respect to the Tumor Suppressor Transcription Factor p53 (p53). Under normal or stressed conditions the *SHP* promoter may be bound by p53, which by the scaffolding protein p53 Binding Protein 1 (p53BP1) recruits the ASCOM complex and transcribes *SHP*.^122^

##### Molecular physiology of the FGF15/19-[FGFR4/βK]-FXR axis

**Fig. 6:**
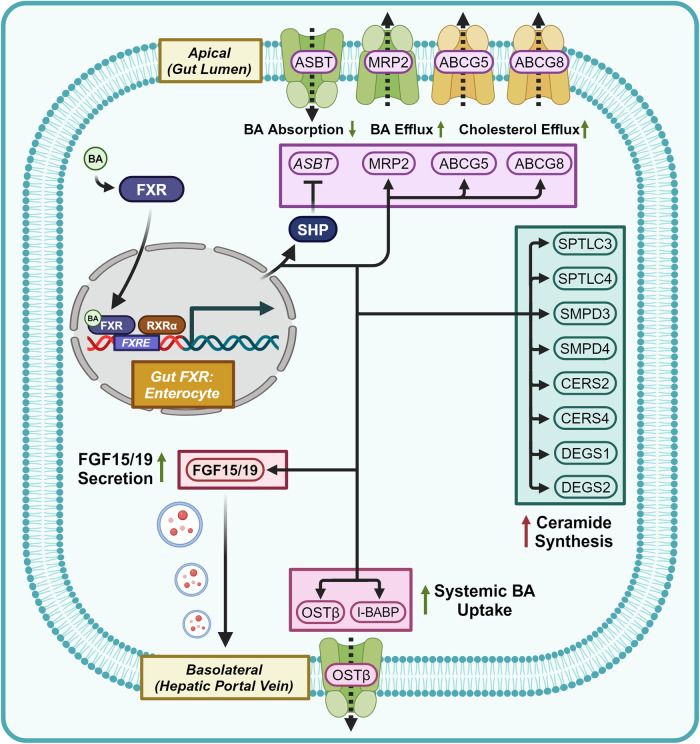
Gut FXR signaling. Gut FXR signaling within enterocytes induces BA/cholesterol efflux, ceramide synthesis, and FGF15/19 secretion. Proteins with a yellow outline represent kinases and phosphatases, while yellow arrows represent phosphorylation/dephosphorylation. Yellow P’s represent phosphate groups. Italicized text refers to genes while normal text refers to proteins. Question marks represent unknown mechanisms. This figure was created with BioRender.com

Alongside direct BA agonism of FXR, the hepatic FXR signaling axis is also modulated by downstream signaling from a complex of the integral membrane protein β-Klotho (βK) and the receptor tyrosine kinase Fibroblast Growth Factor Receptor 4 (FGFR4) (Fig. 6).^123^ Gut FXR agonism stimulates the systemic secretion of the FGFR4/βK complex agonists Fibroblast Growth Factor 15 (FGF15 – mice orthologue) and Fibroblast Growth Factor 19 (FGF19 – human orthologue), serving as long-distance signals for high enteric levels of FXR agonists.^124,125^ Specific to the hunger center of the hypothalamus, a secondary role of FGF15/19 is to suppress appetite by inhibiting Agouti-Related Peptide (AgRP) and Neuropeptide Y (NPY) neurons.^126,127^

In the liver, FGFR4/βK complex agonism phosphorylates the adaptor protein Fibroblast Growth Factor Receptor Substrate 2 (FRS2) and recruits Growth Factor Receptor Bound Protein 2 (Grb2), Grb2 Associated Binding Protein 1 (Gab1), and Src Homology Region 2 Domain-Containing Phosphatase-2 (SHP2).^128^ SHP2 binding to GAB1 promotes the dephosphorylation of Phosphoprotein Associated with Glycosphingolipid-Enriched Microdomains (PAG), preventing the membrane anchoring of the Proto-Oncogene Tyrosine-Protein Kinase Src (Src) inhibitory kinase C-Terminal Src Kinase (Csk).^129^ The release of Csk-mediated inhibition allows for Src to phosphorylate Y67 of FXR and promote nuclear translocation and transcriptional regulation.^128,130^ This is denoted as the [FGFR4/βK]-FXR axis. Grb2 additionally recruits the Ras guanine nucleotide exchange factor Son of Sevenless (Sos), which in conjunction with Src activity activates the Ras-Raf-MEK-ERK signaling cascade, resulting in the phosphorylation and activation of Extracellular Signal-Related Kinase (ERK).^123,131^ This is known as the [FGFR4/βK]-ERK axis.

Alongside FGF15/19 secretion, gut FXR agonism induces the systemic secretion of ceramides. Gut FXR transcriptionally enhances the expression of the following ceramide synthetic proteins: Serine Palmitoyltransferase Long Chain Base Subunit 3,4 (SPTLC3, SPTLC4), Sphingomyelin Phosphodiesterase 3,4 (SMPD3, SMPD4), Ceramide Synthase 2,4 (CERS2, CERS4), and Delta 4-Desaturase Sphingolipid 1,2 (DEGS1, DEGS2).^132^

##### Master regulators of BA homeostasis—FXR and the FGF15/19-[FGFR4/βK]-FXR axis

**Fig. 7:**
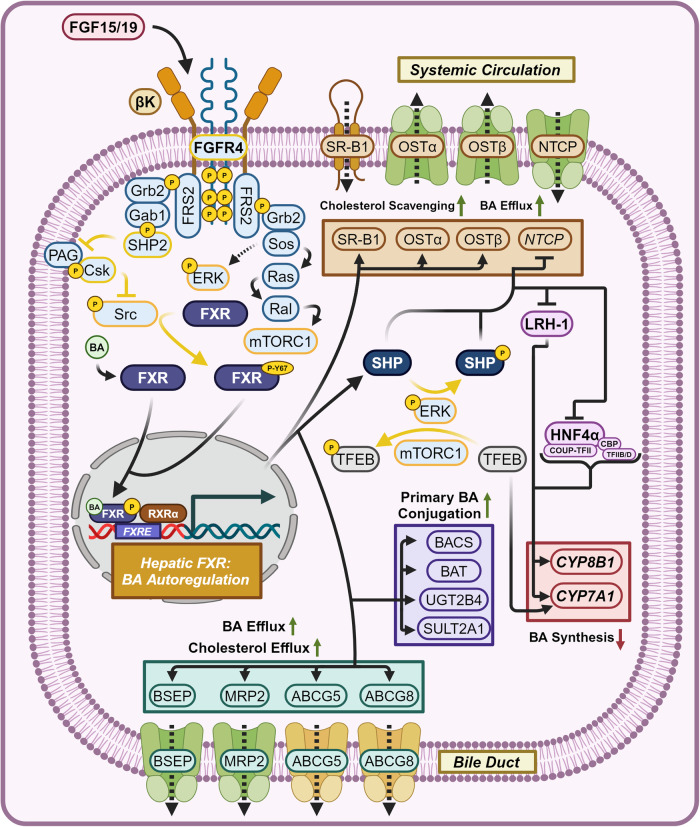
Hepatic FXR signaling—BA autoregulation. Hepatic FXR signaling pertaining to BA autoregulation. Hepatic FXR agonism increases BA/cholesterol efflux, primary BA conjugation, and represses BA synthesis. Dashed mechanism arrows represent the Ras-Raf-MEK signaling cascade, while dashed arrows through a protein represent the direction of substrate flux. Proteins with a yellow outline represent kinases and phosphatases, while yellow arrows represent phosphorylation/dephosphorylation. Yellow P’s represent phosphate groups. Italicized text refers to genes while normal text refers to proteins. Question marks represent unknown mechanisms. This figure was created with BioRender.com

Feedback inhibition for BA synthesis is mediated by two mechanisms, one directing from FXR itself, and another originating from agonized FGFR4/βK complex (Fig. 7). BA agonized FXR induces the expression of SHP, while the downstream actions of agonized [FGFR4/βK]-ERK axis mediated phosphorylation of SHP prevents its proteasomal degradation, both leading to increased SHP levels.^133,134^ SHP dimerizes and directly inhibits the activity of the transcription factors LRH-1 and HNF4α, in addition to inducing the epigenetic repression of their target genes, preventing the transcription of *CYP7A1* and *CYP8B1*. SHP-mediated inhibition and epigenetic repression of LRH-1 target genes represses its own transcription, forming a negative feedback loop.^135–138^ HNF4α-induced *CYP7A1* and *CYP8B1* expression is dependent on the recruitment of COUP Transcription Factor 2 (COUP-TFII), the histone acetyl transferase CREB Binding Protein (CBP), Transcription Factors IID (TFIID), and IIB (TFIIB).^139–141^ In addition, Sos activates Ras, which agonizes Ras-like protein (Ral) and subsequently Mammalian Target of Rapamycin Complex 1 (mTORC1). mTORC1 and ERK phosphorylate and prevent the nuclear translocation of Transcription Factor EB (TFEB), preventing TFEB-induced *CYP7A1* expression.^142,143^

Repression of *CYP7A1* and *CYP8B1* directly lowers metabolic flux through the classic pathway of BA synthesis.^137,144–146^ This two-sided negative-feedback loop lowers the overall BA pool size via *CYP7A1* repression and composition via *CYP8B1* repression, shunting BA synthesis into the alternative pathway and producing the strong FXR agonist CDCA. This results in a smaller BA pool that is more biased in composition towards FXR agonism. FXR is therefore referred to as the master regulator of BA synthesis.^135,147^ In addition, FXR agonism can help prevent systemic BA overload-induced toxicity. High concentrations of BAs and FXR-SHP axis agonism represses hepatic NTCP expression and gut ASBT expression to prevent further BA entry, while FXR induces BSEP, MRP2, I-BABP, OSTα (liver-specific), and OSTβ to increase BA fecal excretion.^92,148^ BACS, BAT, UGT2B4, and SULT2A1 are also upregulated by FXR to increase the rate at which hepatic BAs efflux into the biliary tree.^149^ Sulfation reduces gut BA reabsorption, assisting in the elimination of excess BAs.^17^

##### The FGF15/19-[FGFR4/βK]-FXR axis modulates triglyceride metabolism

**Fig. 8:**
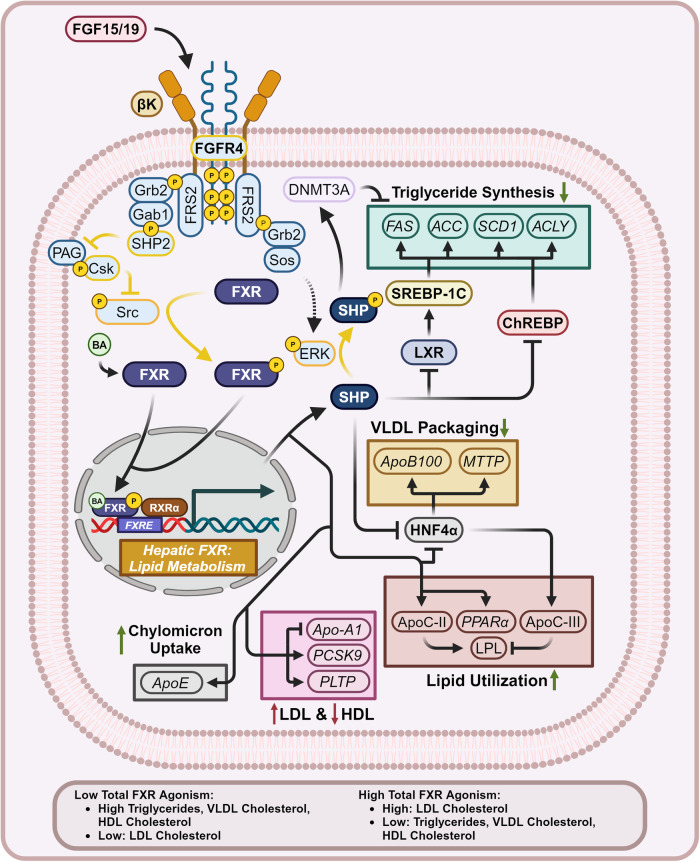
Hepatic FXR signaling—lipid metabolism. Hepatic FXR signaling pertaining to lipid metabolism represses triglyceride synthesis, VLDL packaging, increases lipid utilization, chylomicron uptake, and promotes a pro-atherogenic serum lipid profile. Dashed mechanism arrows represent the Ras-Raf-MEK signaling cascade, while dashed arrows through a protein represent the direction of substrate flux. Proteins with a yellow outline represent kinases and phosphatases, while yellow arrows represent phosphorylation/dephosphorylation. Yellow P’s represent phosphate groups. Italicized text refers to genes while normal text refers to proteins. Question marks represent unknown mechanisms. This figure was created with BioRender.com

Low FXR agonism enhances the rate of cholesterol excretion while high FXR agonism represses the synthesis of triglycerides (Fig. 8). FXR-induced SHP can dimerize with the transcription factor liver X receptor (LXR) and inhibits its induction of sterol regulatory element-binding protein 1C (SREBP-1C), the master transcriptional regulator of triglyceride synthesis.^150,151^ In addition, [FGFR4/βK]-ERK axis activation represses SREBP-1C activity by inducing ERK phosphorylation of SHP, which recruits DNA Methyltransferase-3A (DNMT3A) to epigenetically repress SREBP-1C target genes.^152^ Repression of SREBP-1C activity directly reduces the transcription of Fatty Acid Synthase (*FAS*), Acetyl-CoA Carboxylase (*ACC*), Stearoyl-CoA Desaturase enzyme 1 (*SCD1*), and ATP Citrate Lyase (*ACLY*) genes, key players in triglyceride synthesis. In addition, FXR-SHP axis activation inhibits carbohydrate response element binding protein (ChREBP), another transcription factor which when activated by glucose metabolites induces lipogenesis by upregulating *FAS*, *SCD1*, *ACC*, and *ACLY*.^153^ FXR has been shown to induce the expression of Apolipoprotein CII (ApoC-II) and competitively inhibit the HNF4α-mediated transcription of Apolipoprotein CIII (ApoC-III). ApoC-II expression and ApoC-III repression increase the activity of lipoprotein lipase (LPL), allowing for a more efficient removal of triglycerides out of the systemic circulation into peripheral tissue.^154–156^ Furthermore, FXR has been experimentally shown to induce peroxisomal proliferator-activated receptor alpha (PPARα), a key transcription factor responsible for the catabolism of fatty acids.^157,158^

##### FXR modulation of cholesterol metabolism

FXR-SHP axis activation additionally represses very-low density lipoprotein (VLDL) secretion by SHP dimerization with HNF4α, directly inhibiting it and leading to the epigenetic repression of its target genes. This decreases the rate at which the liver distributes cholesterol and triglycerides. When inhibited, HNF4α fails to induce the expression of downstream *Apolipoprotein B100* (*ApoB100*) and *Microsomal Triglyceride Transfer Protein* (*MTTP*) genes, both of which are essential to VLDL packaging and synthesis.^159^ Although VLDL excretion is reduced, FXR-mediated repression of BA synthesis inhibits cholesterol excretion, allowing for it to accumulate hepatically. Resulting from such, the reduced quantity of VLDL produced will now be cholesterol rich, eventually producing cholesterol-rich low-density lipoproteins (LDL). Long-term FXR agonism contributes towards a potentially pro-atherogenic state due to an RXRα-independent negative regulatory FXRE within the *Apolipoprotein A1* (*Apo-A1*) gene. Apo-A1 is the core structural lipoprotein required for high-density lipoprotein (HDL)-mediated scavenging of systemic cholesterol, necessarily fighting against atherosclerotic plaque development.^160^ FXR activity additionally stimulates the expression of Apolipoprotein E (ApoE) and phospholipid transfer protein (PLTP). Functionally this results in enhanced chylomicron/LDL uptake into the liver, in addition to increasing the rate at which HDL particles uptake phospholipids from VLDL, converting them into LDL.^161^

FXR-mediated changes to serum cholesterol are further complicated by the FXR-induced expression of HDL Scavenger Receptor Class B Type 1 (SR-B1), which increases the hepatic uptake of HDL cholesterol for biliary excretion, further reducing serum HDL cholesterol.^162,163^ FXR signaling attempts to ameliorate this systemic cholesterol excess by increasing the expression of hepatic and gut ATP Binding Cassette Subfamily G Members 5 and 8 (ABCG5) (ABCG8), transporters which serve to efflux hepatic cholesterol into the bile canaliculi and to return cholesterol absorbed by enterocytes back into the gut lumen.^163–166^ Although this may offset some further cholesterol accumulation, increased cholesterol content within the biliary tree without BAs may increase the risk for gallstones.^167^ Another compensatory activity of highly agonized FXR-SHP-axis activity is to reduce the expression of Proprotein Convertase Subtilisin/Kexin Type 9 (PCSK9), increasing the activity of the LDL Receptor (LDLr).^168,169^ However, it needs to be emphasized that even if more LDL cholesterol is absorbed into the liver, the same problem remains, the liver has no active metabolic pathway for large quantity cholesterol excretion.

##### Physiological implications—double-edged FXR lipid phenotypes

Cumulatively, it is then very helpful to characterize the impact of FXR agonism on lipid metabolism into two different states of FXR activity. Within a state of low total FXR activity SREBP-1C activity is high, HDL synthesis is not repressed, and cholesterol is being metabolized into BAs and excreted, producing a state of high triglycerides, VLDL cholesterol, HDL cholesterol, and low LDL cholesterol. In a state of high total FXR activity, SREBP-1C activity is low, HDL synthesis is repressed, and cholesterol is accumulating, producing a state of low triglycerides, VLDL cholesterol, HDL cholesterol, and high LDL cholesterol.

##### The FGF15/19-[FGFR4/βK]-FXR axis modulates carbohydrate metabolism

**Fig. 9:**
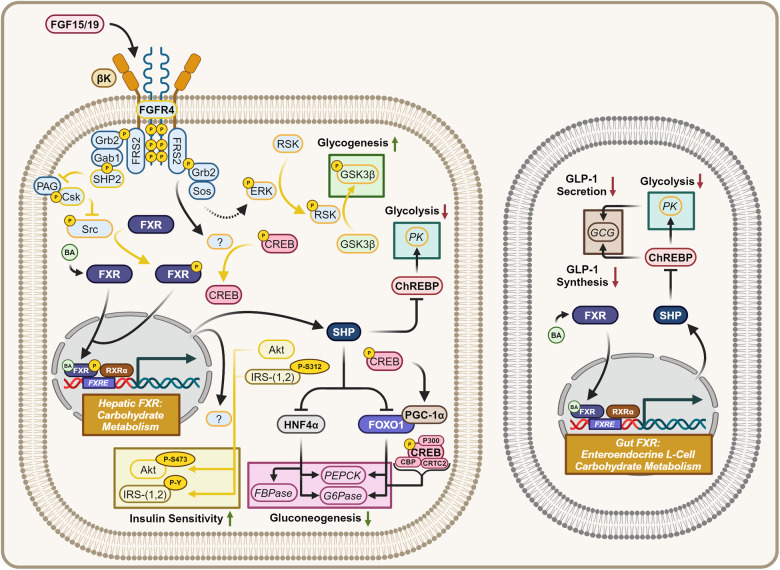
Hepatic/gut FXR signaling—carbohydrate metabolism. Hepatic and gut enteroendocrine L-cell FXR signaling pertaining to carbohydrate metabolism. FXR agonism increases glycogenesis, insulin sensitivity, represses gluconeogenesis, and blunts glycolysis. Gut FXR agonism represses GLP-1 synthesis and secretion. Dashed mechanism arrows represent the Ras-Raf-MEK signaling cascade, while dashed arrows through a protein represent the direction of substrate flux. Proteins with a yellow outline represent kinases and phosphatases, while yellow arrows represent phosphorylation/dephosphorylation. Yellow P’s represent phosphate groups. Italicized text refers to genes while normal text refers to proteins. Question marks represent unknown mechanisms. This figure was created with BioRender.com

FXR agonism activates glycogenesis and inhibits gluconeogenesis (Fig. 9). FXR agonism through unknown mechanisms has been found to mimic insulin and increase insulin sensitivity by inducing the downstream phosphorylation of S473 on Protein Kinase B (Akt). In addition, the dephosphorylation of S312 and the phosphorylation of Tyr residues on Insulin Receptor Substrates 1/2 (IRS1/2) have been observed.^170–173^ FXR activated Akt in addition to [FGFR4/βK]-ERK axis activated Ribosomal-S6-kinase (RSK) phosphorylates and inhibits Glycogen Synthase Kinase 3 Beta (GSK3β).^174,175^ GSK3β is a physiological inhibitor of glycogenesis, whose inhibition leads to positive glycogenic signaling.

FXR-SHP axis activation inhibits HNF4α−induced transcription of Phosphoenolpyruvate Carboxykinase (*PEPCK*), Fructose-1,6-Bisphosphatase (*FBPase*), and Glucose-6-Phosphatase (*G6Pase*) genes. FXR-SHP axis activation also inhibits the transcription factor Forkhead Box Protein O1 (FOXO1) and its induced expression of *PEPCK* and *G6Pase*.^176–178^ FXR-SHP axis activation therefore has an inhibitory role on the expression of three of the four rate-limiting proteins within hepatic gluconeogenesis (G6Pase, FBPase, PEPCK). In contrast to the general trend of enhancing glucose tolerance, FXR agonism blunts both hepatic and enteroendocrine intestinal L-cell glycolytic flux. This is mechanistically mediated by FXR-SHP axis inhibition of ChREBP, repressing the transcription of Pyruvate Kinase (*PK*).^153^ Within enteroendocrine intestinal L-cells this decreases the synthesis and secretion of Glucagon-Like Peptide-1 (GLP-1). Decreased GLP-1 synthesis is explained by reduced ChREBP-induced transcription of GLP-1’s precursor Proglucagon (*GCG*), while hindered GLP-1 secretion is caused by reduced glycolytic flux and hence reduced ATP-driven exocytosis.^179^

##### FGF15/19-[FGFR4/βK] signaling represses hepatic gluconeogenesis

An unknown protein downstream of [FGFR4/βK] complex agonism dephosphorylates the transcription factor cyclic adenosine monophosphate (cAMP) response element binding protein (CREB). The dephosphorylation of pCREB to produce CREB reduces glucagon or adrenergic-induced gluconeogenesis in the short-term by not trans-activating cAMP response element (CRE) mediated expression of G6Pase, PEPCK, and FOXO1. The histone acetyl transferase CBP, Histone Acetyltransferase p300 (p300), and the coactivator CREB regulated transcription coactivator 2 (CRTC2) must be bound by pCREB to epigenetically activate gene expresson.^178,180^ FGF15/19 serves as a long-distance signal for nutrition by further inhibiting starvation-induced gluconeogenesis, in which inactivated CREB cannot induce the expression of the FOXO1 coactivator peroxisome proliferator-activated receptor-gamma coactivator 1 alpha (PGC-1α), drastically suppressing the expression of G6Pase and PEPCK.^181–183^

Viewing carbohydrates, the hepatic FGF15/19-[FGFR4/βK]-FXR axis centrally functions to potentiate insulin-induced glucose uptake, signals for the short-term storage of dietary carbohydrates as glycogen, serves to prevent the unneeded synthesis of carbohydrates in the presence of recently consumed nutrients, hinders glycolytic flux, and intestinally blunts incretin-like activity.

##### FXR modulation of amino acid metabolism

**Fig. 10:**
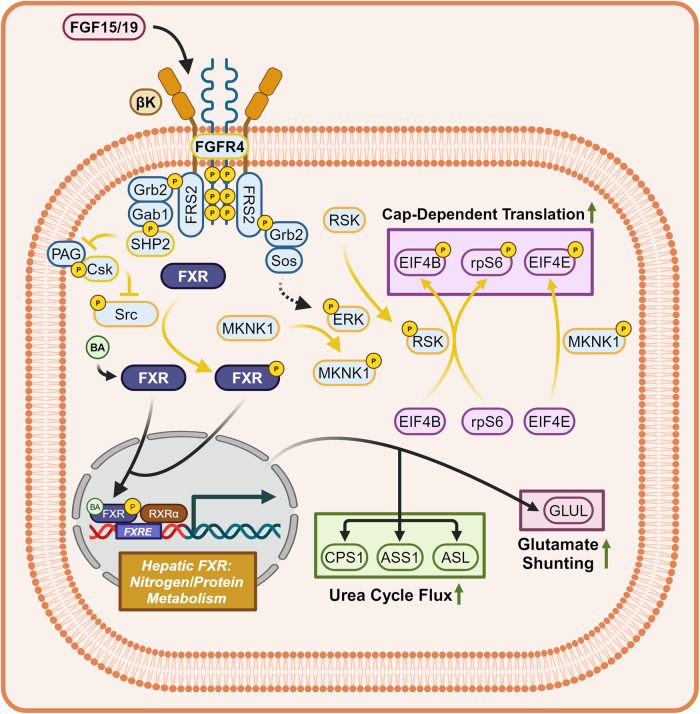
Hepatic FXR signaling—amino acid metabolism. Hepatic FXR signaling pertaining to nitrogen and protein metabolism. Hepatic FXR agonism increases urea cycle flux, glutamate shunting, and increases cap-dependent translation. Dashed mechanism arrows represent the Ras-Raf-MEK signaling cascade, while dashed arrows through a protein represent the direction of substrate flux. Proteins with a yellow outline represent kinases and phosphatases, while yellow arrows represent phosphorylation/dephosphorylation. Yellow P’s represent phosphate groups. Italicized text refers to genes while normal text refers to proteins. Question marks represent unknown mechanisms. This figure was created with BioRender.com

FXR agonism increases the flux of excess amino acids through the urea cycle and signals for the utilization of recently ingested amino acids within protein synthesis (Fig. 10). In mice, hepatic FXR agonism plays a wide role in sustaining the proper metabolism of amino acids. This occurs by improving glutamate shunting of ammonia via Glutamine Synthetase (GLUL) induction and flux through the urea cycle by inducing Carbamoyl Phosphate Synthetase-1 (CPS1), Arginosuccinate Synthase 1 (ASS1), and Arginosuccinate Lyase (ASL).^184,185^ [FGFR4/βK]-ERK axis activation phosphorylates and activates RSK and MAP Kinases-Interacting Serine/Threonine-Protein Kinase 1 (MKNK1), resulting in the phosphorylation of Eukaryotic Translation Initiation Factors 4B (EIF4B) and 4E (EIF4E), respectively.^175^ The phosphorylation of EIF4B and EIF4E are crucial for the initiation of eukaryotic cap-dependent translation.^186^ RSK also phosphorylates Ribosomal Protein S6 (rpS6), a modulator of ribosomal translation which additionally increases the efficacy of cap-dependent translation.^175^

##### General mechanisms—FXR dampens hepatic and systemic inflammation

**Fig. 11:**
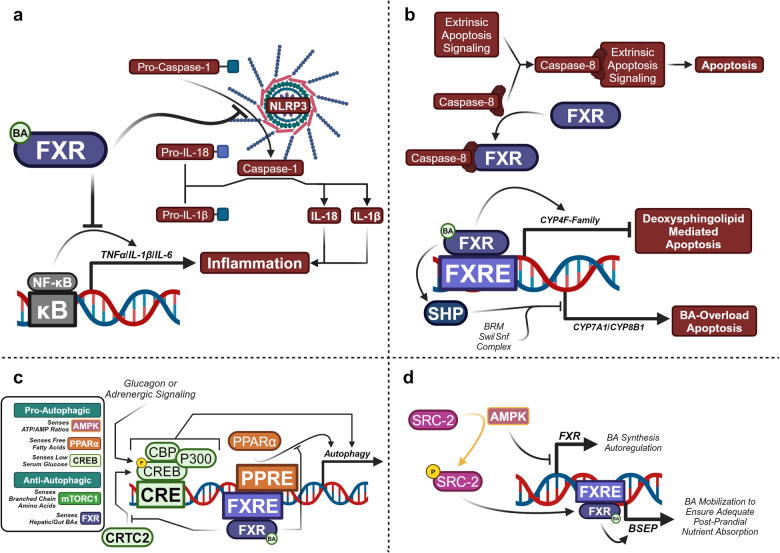
FXR signaling is anti-inflammatory, anti-apoptotic, and anti-autophagic. **a** FXR activity inhibits NLRP3/Caspase-1 and NF-κB induced inflammation. **b** Non-ligand-bound FXR is anti-apoptotic by preventing extrinsic apoptosis, while ligand-bound FXR prevents BA-overload and deoxysphingolipid induced apoptosis. **c** FXR activity is anti-autophagic by preventing pCREB/PPARα-mediated autophagic gene expression. **d** Starvation-induced AMPK enhances BA synthesis and BA mobilization to ensure adequate nutrient absorption. Proteins with a golden outline indicate kinases, while golden arrows represent phosphorylation. This figure was created with BioRender.com

Alongside high expression within the gut and liver, FXR is also expressed within immune cells.^187^ FXR exhibits potent anti-inflammatory activity in peripheral monocytes, in human and mouse myeloid cells, dendritic cells, and in hepatic natural killer T cells.^188–191^ FXR agonism exerts anti-inflammatory properties by physically inhibiting the formation of the NLR Family Pyrin Domain Containing 3 (NLRP3) inflammasome (Fig. 11a). This is accomplished by physically preventing the association of NLRP3 and pro-caspase-1, preventing the maturation of the pro-inflammatory cytokines Interleukin 1 Beta (IL-1β) and Interleukin 18 (IL-18).^192^ In addition, FXR agonism has been found to inhibit the binding of the transcription factor Nuclear Factor Kappa-Light-Chain-Enhancer of Activated B Cell (NF-κB) to cis-regulatory κB sites of pro-inflammatory genes, such as *IL-1β*, Interleukin 6 *(IL-6)*, and Tumor Necrosis Factor Alpha *(TNFα)*.^146,193^ Although FXR mediates hepatic anti-inflammatory properties by the two mechanisms mentioned, it is still unknown if the total anti-inflammatory role of FXR is entirely receptor-mediated or a downstream effect of reducing hepatic lipid content.

##### FXR is anti-apoptotic and disrupts autophagy

Hepatocytes under significant cellular stress may be vulnerable to apoptosis, especially under hepatic conditions such as NAFLD or NASH.^194^ FXR plays a significant hepatoprotective role against apoptosis in which non-ligand-bound FXR binds to the death effector domain of caspase-8, inhibiting extrinsic apoptosis (Fig. 11b).^195^ FXR additionally prevents apoptosis by inducing Cytochrome P450 Family 4 Subfamily F (CYP4F)-mediated metabolism of pro-apoptotic deoxysphingolipids and by preventing BA-induced apoptosis by repressing CYP7A1/CYP8B1 expression.^196–198^

Under extensive whole body starvation, autophagy recruits intracellular macromolecules to the lysosome to be broken down for energy to ensure physiological survival.^199,200^ Within the liver pCREB and CRTC2 greatly contribute to the starvation-induced expression of autophagic genes, likely induced by glucagon or adrenergic signaling. Agonized FXR functionally disrupts the pCREB/CRTC2 interaction preventing autophagy, even during fasting (Fig. 11c).^201^ FXR may also directly bind to autophagic gene promoters and suppress their transcriptional activity. FXR’s suppression over autophagic gene expression is nuanced since the same binding site is regulated by pro-autophagic PPARα, allowing for nutrient-sensitive control over autophagy in addition to that provided by Adenosine Monophosphate (AMP)-Activated Protein Kinase (AMPK) sensing the ATP/AMP balance and mTORC1 sensing free branched chain amino acids.^202^ Further interplay between FXR and the starvation response has been discovered, in which activated AMPK transcriptionally represses FXR and stimulates Steroid Receptor Coactivator-2 (SRC-2) to coactivate FXR-induced expression of BSEP (Fig. 11d).^203,204^ In anticipation of upcoming dietary nutrition, this allows for a transient release from BA synthesis autoregulation and increases BA mobilization into the biliary tree, cumulatively increasing the efficacy in which BA-mediated absorption replenishes current depleted nutrient stores.

#### Canonical BA receptor—TGR5, a metabolic and immunological modulator

##### Molecular biology of TGR5

TGR5 is a G-protein coupled receptor encoded by the *GPBAR1* gene. The G_α_ subunit for TGR5 is coupled to Adenylate Cyclase (AC), designating TGR5 as a G_αs_ G-protein.^84^ The ENSEMBL database identifies 75 sequence orthologues for TGR5, where the sequence homology of *Mus musculus* and *Rattus norvegicus* compared to the human sequence are 83.59% and 82.37%, respectively.^99^ As previously stated for FXR, the sequence divergence between species may not allow for a direct correlation between animal models and results seen in human physiology. The following list ranks the top 8 baseline TGR5-expressing organs in terms of nTPM: adipose tissue, breast, gallbladder, smooth muscle, spleen, stomach, kidney, and heart. Although low in documented nTPM, it is also of use to note that TGR5 is expressed within the small intestine, colon, was recently discovered in mice hepatocytes, and in mice is exercise-inducible via the transcription factor Activating Transcription Factor 6 (ATF6) within skeletal muscle. The membrane-bound TGR5 receptor has many metabolic functions, such as increasing basal metabolic rate, whole-body nutrient tolerance, post-prandial satiety, and via Adiponectin (ADIPOQ) signaling induces insulin-sensitivity, reduces gluconeogenesis, reduces lipogenesis, and increases ceramide metabolism. Non-metabolic functions of TGR5 are to protect against hepatic pathology, reduce hepatic and adipose exposure to inflammatory signaling, and to maintain proper biliary function.^205^

##### Epigenetic regulation of *TGR5* transcription

CpG methylation of the *TGR5* promoter has been found to reduce transcription and is associated with both liver-failure and chronic hepatitis B.^206^ Hyper-methylation of the *TGR5* promoter has also been found associated with hepatocellular carcinoma. Although *TGR5* promoter methylation is age-related and higher in patients older than 60, methylation may serve as a potential diagnostic for both acute-on-chronic hepatitis B liver failure and hepatocellular carcinoma.^207^ Besides direct phenotypic association between hepatic pathology and CpG methylation of the *TGR5* promoter, not much research has been conducted directly on the factors driving *TGR5* epigenetic regulation. It is currently theorized that a significant portion of *TGR5* transcriptional regulation is secondary to FXRE activity within its promoter, being directly driven by the epigenetics, post-transcriptional, and post-translational modifications modulating FXR activity.^208^ TGR5 agonists have been found to induce *SIRT1* and *Sirtuin 3* (*SIRT3)* transcription via activation of PGC-1α.^209^ TGR5-induced SIRT1 expression may further modulate FXR activity, establishing a mutual regulatory relationship.

##### TGR5 agonism drives energy utilization and mitochondrial biogenesis

**Fig. 12:**
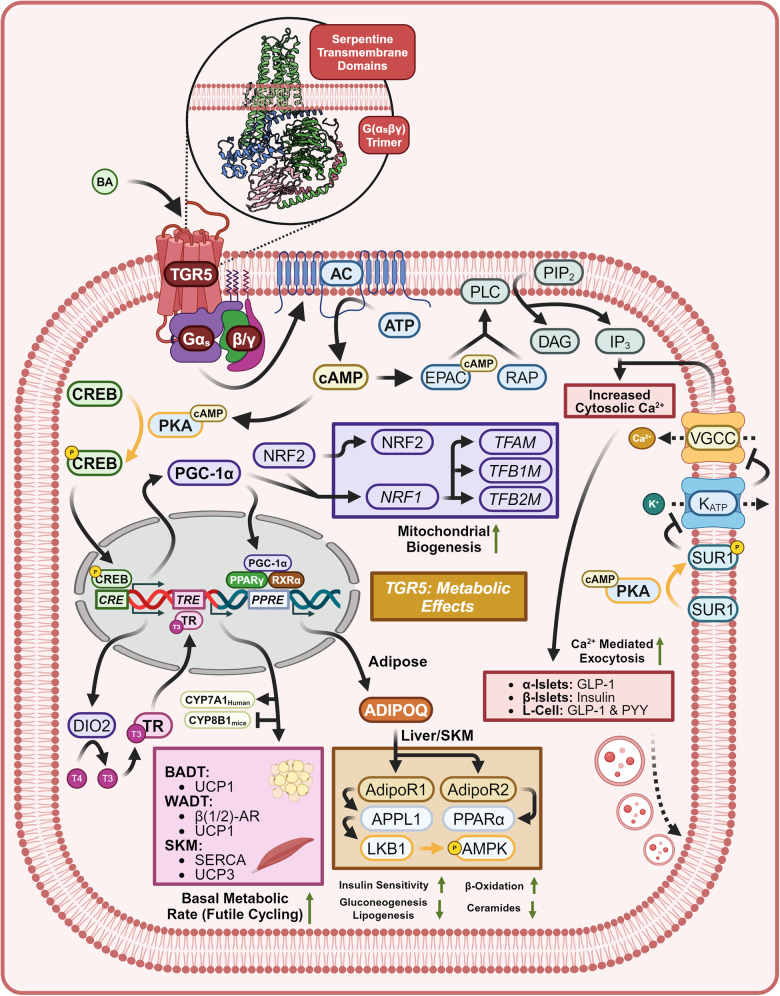
TGR5 signaling—metabolic effects. Metabolic effects of TGR5 signaling, crystal structure shown is PDB 7CFM.^631^ TGR5 agonism induces incretin release, upregulates mitochondrial biogenesis and basal metabolic rate, and leads to ADIPOQ synthesis, increasing carbohydrate/lipid tolerance and reducing ceramide concentrations. Proteins with a yellow outline represent kinases and phosphatases, while yellow arrows represent phosphorylation/dephosphorylation. Yellow P’s represent phosphate groups. Question marks represent unknown mechanisms. Dashed mechanism arrows represent vesicle exocytosis, while dashed arrows through a protein describe the direction of ion flow. Italicized text refers to genes while normal text refers to proteins. This figure was created with BioRender.com

Metabolically relevant TGR5 agonism stimulates AC to produce cAMP and activates Protein Kinase A (PKA) (Fig. 12).^85,210–212^ CREB is phosphorylated by PKA to produce pCREB. pCREB binds to the CRE and induces the adipocyte/skeletal muscle expression of Type 2 Iodothyronine Deiodinase 2 (DIO2) and PGC-1α.^213,214^ Increased expression of DIO2 directly increases the rate of conversion of Tetraiodothyronine (T4) into Triiodothyronine (T3). When the more active form of the hormone T3 binds to the Thyroid Hormone Receptor (TR), TR acts as a transcription factor for genes with Thyroid Response Elements (TREs). In mice, TGR5-induced TR-agonism represses *CYP8B1* expression without the presence of a TRE, while in humans TR-agonism induces the expression of *CYP7A1*.^215–219^ In brown adipose tissue (BADT) TR agonism induces Uncoupling Protein 1 (UCP1) which by establishing a futile cycle of mitochondrial proton shuttling increases energy usage via thermogenesis.^220,221^ In white adipose tissue (WADT) TR agonism induces the expression of β1 and β2 Adrenergic Receptors (β1-AR) (β2-AR) along with UCP1. Spare adrenergic receptors increase WADT sensitivity to adrenergic-induced Hormone-sensitive Lipase (HSL) activity.^222–224^ In skeletal muscle (SKM) Uncoupling Protein 3 (UCP3) is induced by TR agonism along with Sarcoplasmic/ER Calcium-Dependent ATPase (SERCA), resulting in energetically futile cycles of protons at the mitochondria and calcium at the sarcoplasmic reticulum.^225–227^ In all three target tissues, TGR5 agonism increases energy consumption, upregulates mitochondrial oxidative phosphorylation, and hence increases basal metabolic rate.^213,228^

Following the same pattern of energy usage, PGC-1α functions as the master regulator of mitochondrial biogenesis, leads to the adipose exocytosis of ADIPOQ, and enhances adipose UCP1 expression.^229–231^ The coactivator function of PGC-1α in adipose tissue increases the transcriptional activity of Nuclear Respiratory Factor 2 (NRF2), which induces the expression of Nuclear Respiratory Factor 1 (NRF1) by binding to the antioxidant response element (ARE).^232^ NRF1 and NRF2 induce the nuclear expression of mitochondrial respiratory chain proteins.^233^ NRF1 induces the mitochondrial transcription of Transcription Factors A (TFAM), B1 (TFB1M), and B2 (TFB2M), resulting in preparation for mitochondrial biogenesis.^234–236^ PGC-1α is additionally a coactivator for Peroxisomal Proliferator-Activated Receptor Gamma (PPARγ)/RXRα dimers. When the ternary complex binds to Peroxisomal Proliferator-Activated Receptor Response Elements (PPREs), adipocytes synthesize and secrete the adipokine ADIPOQ.^230,231,237^ Systemically, Adiponectin Receptor 1 (AdipoR1) agonism increases whole-body insulin sensitivity by activating Adaptor Protein, Phosphotyrosine Interacting with PH Domain and Leucine Zipper 1 (APPL1), promoting the binding of IRS1/2 to the insulin receptor. In addition, AdipoR1 agonism activates Liver Kinase B1 (LKB1) leading to the phosphorylation and activation of AMPK, which represses hepatic gluconeogenesis, lipogenesis, and cholesterogenesis.^238–241^ Adiponectin Receptor 2 (AdipoR2) agonism increases β-oxidation of fatty acids within the liver and adipose tissue by inducing PPARα.^242,243^ Agonism of both AdipoR1/R2 activates receptor intrinsic ceramidase activity, protecting against the negative metabolic side effects of ceramides.^244–248^

##### TGR5 agonism drives incretin release

Within pancreatic β-islets, α-islets, and enteroendocrine L-cells, TGR5 agonism leads to calcium-induced exocytosis by two mechanisms. The first of which involves cAMP activation of Exchange Protein Activated by cAMP (EPAC), the recruitment and activation of Receptor-Associated Protein (RAP), Phospholipase C (PLC) mediated hydrolysis of Phosphoinositide (PIP_2_) to Inositol Triphosphate (IP_3_) / Diacylglycerol (DAG), and ER calcium release.^249,250^ Specific to the pancreas, EPAC additionally stimulates Rab-interacting Molecule 2 (RIM2) and Piccolo to increase calcium-induced exocytotic granule trafficking and fusion.^251–253^ The second mechanism involves PKA-mediated phosphorylation of Sulfonylurea Receptor 1 (SUR1), inhibition of ATP-Sensitive Potassium Channels (K_ATP_), membrane depolarization, Voltage-gated Calcium Channel (VGCC) activation, and calcium-induced calcium release from the ER.^254–257^ In β-islets, this leads to the exocytosis of insulin.^249,254,258^ In enteroendocrine intestinal L-cells this leads to the exocytosis of GLP-1 and Peptide YY (PYY), resulting in enhanced insulin secretion, reduced rates of gastric emptying, and appetite suppression.^259–263^ By the same two mechanisms pancreatic α-islets secrete GLP-1 instead of glucagon. This transition is explained by α-islet-specific EPAC activity, which induces the expression of proprotein convertase 1 (PC1), biasing alternative splicing of proglucagon mRNA to favor the GLP-1 transcript instead of the glucagon transcript.^249^ Cumulatively under a metabolic lens, TGR5 agonism enhances basal metabolic rate by increasing thermogenic futile cycling, lipolysis, and mitochondrial biogenesis, while leading to increased nutrient tolerance, insulin sensitivity, satiety, and ceramide metabolism.

##### TGR5 promotes hepatic mitochondrial function, vasodilation, and biliary flow

**Fig. 13:**
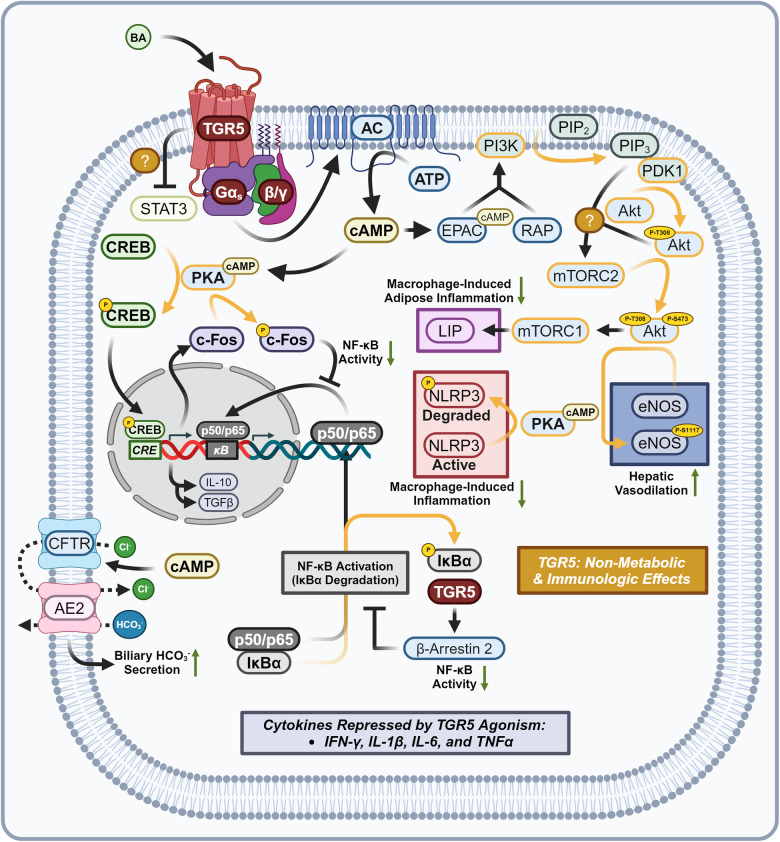
TGR5 signaling—non-metabolic and immunologic effects. Non-metabolic and immunologic effects of TGR5 signaling, crystal structure shown is PDB 7CFM. TGR5 agonism represses macrophage/adipocyte-specific inflammation, enhances hepatic vasodilation, biliary flow, represses pro-inflammatory NF-κB activity, and induces IL-10/TGFβ expression. Proteins with a yellow outline represent kinases and phosphatases, while yellow arrows represent phosphorylation/dephosphorylation. Yellow P’s represent phosphate groups. Question marks represent unknown mechanisms. Dashed mechanism arrows represent vesicle exocytosis, while dashed arrows through a protein describe the direction of ion flow. Italicized text refers to genes while normal text refers to proteins. This figure was created with BioRender.com

TGR5-induced hepatic endothelial cAMP synthesis results in EPAC/RAP-mediated stimulation of Phosphoinositide 3-Kinase (PI3K) and phosphorylation of PIP_2_ into phosphatidylinositol trisphosphate (PIP_3_) (Fig. 13).^264^ PIP_3_ synthesis activates Pyruvate Dehydrogenase Kinase 1 (PDK1) leading to the phosphorylation of Akt at T308. Two models currently exist, in which either PIP_3_ or pT308-Akt activate Mammalian Target of Rapamycin Complex 2 (mTORC2), resulting in the phosphorylation of Akt at S473.^265^ pT308/pS473-Akt along with PKA directly phosphorylate and activate Endothelial Nitric Oxide Synthase (eNOS) at S1117, maintaining hepatic endothelial mitochondrial function and vasodilation.^266–268^ TGR5-induced cAMP-based agonism provides additional benefits by activating Cystic Fibrosis Transmembrane Conductance Regulator (CFTR). CFTR agonism leads to chloride secretion and soon after bicarbonate exchange by Anion Exchange Protein 2 (AE2) into the bile duct, maintaining bile flow and protecting against cholangitis/cirrhosis.^269–273^

##### General mechanisms—TGR5 exerts potent anti-inflammatory activity

TGR5 is extensively expressed within cells derived from the myeloid lineage and is anti-inflammatory.^274^ Immunologically, TGR5-induced pT308/pS473-Akt activates mTORC1, resulting in the differential translation of CCAAT/enhancer-binding protein β (CEBP/β) mRNA to produce Liver-Enriched Inhibitory Protein (LIP).^275–277^ LIP production reduces the expression of pro-inflammatory TNFα and IL-6, reducing both macrophage migration and insulin resistance-associated inflammation within adipose tissue.^278^ TGR5 agonism inhibits NF-κB pathway activation within macrophages, Kupffer cells, and hepatocytes, reducing the expression of the following cytokines: Interferon Gama (IFNγ), Interleukin-1β (IL-1β), IL-2, IL-6, and TNFα.^279–281^ Mechanistically this is accomplished by inhibiting the phosphorylation of NF-κB Inhibitor Alpha (IκBα) and inhibiting the translocation of the NF-κB transcription complex p50 and p65 (p50/p65). TGR5 activation recruits β-Arrestin 2 and inhibits the phosphorylation and proteasomal degradation of IκBα.^282^ TGR5 provoked pCREB activation induces the expression of the transcription factor c-Fos (c-Fos), which when phosphorylated by PKA prevents p50/p65 from binding to κB sites.^283^ In addition, pCREB activation induces the expression of Interleukin 10 (IL-10), and Transforming Growth Factor Beta (TGFβ).^284,285^ Further reductions in pro-inflammatory cytokines are seen by TGR5-AC-PKA axis activity, leading to the ubiquitination and destruction of the NLRP3 inflammasome.^286^ NLRP3 cessation directly leads to decreased concentrations of macrophage-relevant pro-inflammatory cytokines IL-1β and IL-18.^287,288^ Phenotypically, TGR5 agonism has been found to switch macrophages away from the pro-inflammatory M1 phenotype towards the anti-inflammatory M2 phenotype.^278,284,289–291^

#### Non-canonical BA receptors

##### Sphingosine-1-phosphate receptor 2

Sphingosine-1-phosphate Receptor 2 (S1PR2) is a G_i_ coupled G-protein coupled receptor commonly expressed within hepatocytes, cholangiocytes, stellate cells, intestinal epithelial cells, macrophages, and is directly agonized by conjugated BAs.^292–295^ Downstream of such S1PR2 induces the expression of Sphingosine Kinase 2 (SphK2). Sphk2 converts sphingosine to Sphingosine-1-phosphate (S1P), inhibiting HDAC-1/2, and enhancing the expression of genes associated with hepatic glucose and lipid metabolism such as: SREBP-1C, FAS, Carnitine Palmitoyl Transferase 1A (CPT-1A), PPARγ, PGC-1α, ApoB100, and FXR. Mice *S1PR2*^*−/−*^ and *SphK2*^*−/−*^ models found that the lack of S1PR2-mediated induction of ApoB100 and CPT-1A directly enhances the extent of hepatic steatosis, indicating that S1PR2 plays an important modulatory role in NAFLD pathogenesis.^296^ In addition, S1PR2 may activate ERK, Akt, and c-Jun N-terminal Kinase (JNK) signaling cascades, if bound to glycine conjugated CDCA may induce apoptosis, and stimulates the pro-inflammatory M1 polarization of macrophages via the Gi-PI3K-JNK axis.^293,294,297–299^ Inhibition of S1PR2 activity was found to decrease total serum BAs, reduce cholestatic injury in mice bile duct ligation mice models, restore intestinal mucosal barrier function, and reverse pro-inflammatory M1 macrophage polarization in mice Dextran Sulfate Sodium (DSS)-colitis models.^300,301^

##### Muscarinic receptors 2 & 3

Muscarinic receptors 2 (CHRM2) and 3 (CHRM3) are G_i_ and G_q_ coupled G-protein coupled receptors, respectively, and both may be agonized by taurine conjugated BAs.^302^ CHRM2 and CHRM3 overexpression is associated with the initiation and progression of colon, gastric, and pancreatic cancers.^303–306^ In addition, taurine conjugated LCA has been found to induce cholangiocarcinoma as a downstream result of muscarinic agonism.^307^

##### Vitamin D receptor

The vitamin D receptor (VDR) is a nuclear transcription factor that dimerizes with RXRα, is canonically agonized by vitamin D3, and is expressed extensively within the bones, intestine, kidney, and liver.^308–310^ As time progressed it was discovered that VDR may be agonized by LCA, serving as an intestinal BA receptor.^311^ LCA agonized VDR suppresses *CYP7A1* expression by binding to HNF4α and preventing its association with *CYP7A1* or by inhibiting its interaction with COUP-TFII, CBP, TFIID, and TFIIB.^136,139–141,312^ LCA agonized VDR additionally induces the expression of detoxifying metabolic enzymes such as CYP3A4, SULT2A1, MRP3, and ASBT, which cumulatively enhance the metabolism and excretion of BA acids.^313–316^ VDR agonism suppresses FXR-SHP-axis activity independent of its DNA binding domain, preventing FXR from transactivating FXRE-containing promoters.^317^ In addition, VDR agonism transcriptionally represses *SHP*.^318,319^ Lastly, VDR expression and polymorphism within *VDR* has been found to significantly affect microbial beta diversity, directly modulating the efficacy of secondary BA synthesis.^320,321^ A lack of VDR agonism has been associated with decreased *Lactobacillus* presence, and enhanced *Clostridium* and *Bacteroidetes* presence.

##### Pregnane X receptor

Pregnane X receptor (PXR) is a RXRα heterodimeric transcription factor that is largely expressed within the liver and gut.^322^ PXR is commonly referred to as the xenobiotic sensor since it regulates the expression of many classes of enzymes associated with xenobiotic metabolism.^323^ Upon binding to a xenobiotic agonist, LCA, or 3-oxo-LCA, PXR induces the expression of Cytochrome P450 3 A, 2B, and 2 C subfamilies of phase I metabolic enzymes, phase II enzymes such as glutathione S-transferases, UGTs, and SULTs, and phase III enzymes such as Multidrug Resistance 1 (MDR1), MRP2, and Organic Anion Transporting Polypeptide 2 (OATP2). PXR therefore has a direct role in preventing lipophilic BA-mediated toxicity, such as that which occurs with high LCA concentrations.^324–326^ Activated PXR sequesters HNF4α, preventing it from interacting with PGC-1α, transcriptionally repressing *CYP7A1*.^327,328^ PXR agonism enhances de novo lipogenesis by enhancing expression of SCD1 and various long fatty acid elongases, in addition to sequestering the transcription factor Forkhead Box Protein A2 (FOXA2) and preventing the expression of CPT-1A.^329,330^ Lastly, agonized PXR may directly dimerize with pCREB, reducing its ability to induce gluconeogenesis.^331^

##### Constitutive androstane receptor

The hepatically expressed RXRα heterodimeric transcription factor constitutive androstane receptor (CAR) has high homology to that of PXR. Different from that of PXR, CAR is constitutively active, where androstane metabolites were found to be inverse agonists.^332,333^ Analogous to PXR, CAR induces the expression of enzymes responsible for metabolizing BAs, but it is unsure which BAs modulate CAR transcriptional regulation and to what extent.^323,334,335^

##### Liver X receptors

The LXRs are heterodimer transcription factors with RXRα that are endogenously agonized by oxysterols.^336^ Two genes encode the highly homologous LXR family, *NR1H3* for LXRα and *NR1H2* for LXRβ.^337^ LXRα is found extensively within the liver, adipose tissue, the gut, and macrophages, while LXRβ is expressed ubiquitously.^338^ In rats, LXR activation serves as a sensor for cholesterol over-abundance, enhancing the transcription of *CYP7A1, ABCG5*, and *ABCG8* to increase cholesterol excretion and decrease its absorption.^339,340^ In humans, LXR activation has similar effects on ABCG5 and ABCG5 but reduces BA synthesis by inducing *SHP* transcription.^341–343^ HDCA has been found to be a weak LXRα agonist.^344^

##### Peroxisomal proliferator-activated receptor alpha

PPARα is a transcription factor that under starved conditions and adipose tissue lipolysis induces fatty acid uptake and catabolism.^345,346^ HDCA has been found to directly displace Chromosomal Region Maintenance 1 (CRM1) from the Ran GTPase (Ran)/CRM1/PPARα cytosolic shuttling heterotrimer, allowing for PPARα to locate to the nucleus and modulate the transcription of lipid-catabolic genes.^347^ PPARα agonism has been found to suppress expression of NTCP, OATP1, and upregulate BSEP/MRP3/MRP4 expression, cumulatively enhancing BA efflux from the liver.^348,349^ In addition PPARα reduces NLRP3 inflammasome activation, caspase-1 cleavage, and downstream maturation of IL-1β by inducing the transcription of lncRNA Gm15441.^350^

#### Ceramides, a unifying hypothesis against BA-induced cardiometabolic disease

**Fig. 14:**
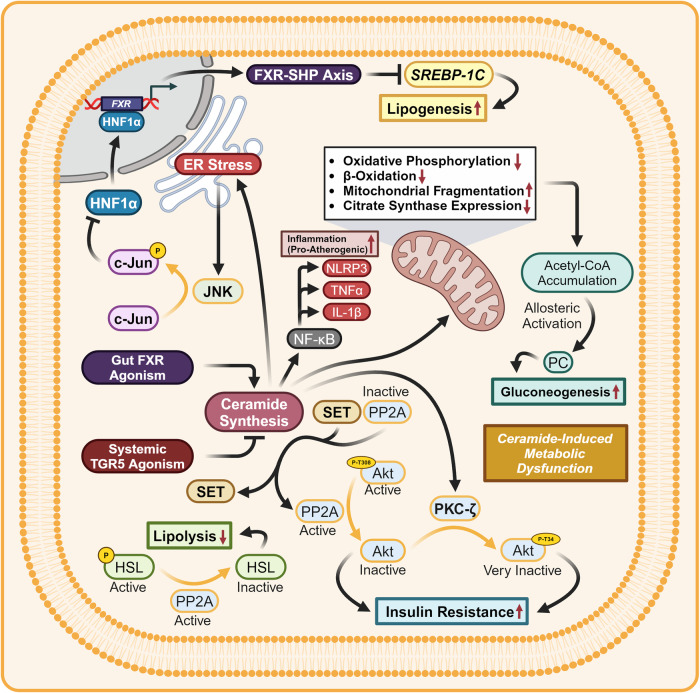
Ceramides, BA-induced culprits behind cardiometabolic disease. Systemic ceramide secretion induced by high gut FXR agonism and low systemic TGR5 agonism increases lipogenesis, gluconeogenesis, pro-atherogenic inflammation, and insulin resistance while inhibiting lipolysis and mitochondrial function. Proteins with a yellow outline represent kinases and phosphatases, while yellow arrows represent phosphorylation/dephosphorylation. Yellow P’s represent phosphate groups. Italicized text refers to genes while normal text refers to proteins. This figure was created with BioRender.com

Abnormal biochemical or physiological changes to the BA pool composition directly alter the ratio between ceramide synthetic gut FXR agonism and ceramide catabolic systemic TGR5 agonism. Resulting increases in ceramide synthesis are directly deterministic for the pathogenesis of cardiometabolic diseases. Ceramides negatively impact the liver by inducing lipogenesis and gluconeogenesis (Fig. 14). Hepatic uptake of gut FXR-induced ceramides has been found to lead to hepatocyte ER stress, the activation of JNK, the phosphorylation of the transcription factor c-Jun, and the inhibition of HNF1α, a key transcription factor responsible for the transcription of the *FXR* gene. This chain of events prevents proper FXR-mediated *SREBP-1C* repression, leading to increases in lipogenesis and overall promotion of the steatotic phenotype of NAFLD/NASH.^124,351^ Hepatic ceramide exposure has also been found to repress the expression of mitochondrial Citrate Synthase (CS), leading to the accumulation of Acetyl-CoA, the allosteric activator of the first enzyme in gluconeogenesis Pyruvate Carboxylase (PC).^132^ Hence, hepatic ceramide exposure directly enhances the gluconeogenic and hyperglycemic phenotype associated with T2DM. Specific to adipose tissue, ceramide exposure directly represses HSL-mediated lipolysis. The protein SET Nuclear Proto-Oncogene (SET) is generally bound to protein phosphatase 2A (PP2A) and inhibits it, but ceramide exposure releases this inhibition. PP2A activation dephosphorylates and inactivates HSL preventing adrenergic G_αs_-cAMP-PKA induced lipolysis.^352–354^ The inhibition of HSL-mediated lipolysis directly predisposes for peripheral adiposity, the hallmark of obesity.

Ceramide exposure to the liver and adipose tissue results in increased insulin resistance and mitochondrial dysfunction. In the liver and adipose tissue activated PP2A dephosphorylates stimulatory pT308 of Akt.^352–355^ In addition, hepatic and adipocyte exposure to ceramides stimulates PKC isoform Zeta (PKC-ξ), leading to the inhibitory phosphorylation of Akt at T34.^356,357^ The inhibition of the central kinase behind the insulin signaling cascade Akt results in impaired glucose tolerance and insulin resistance, the key phenotype of T2DM. In addition, ceramide synthesis directly stimulates the nuclear translocation of NF-κB, inducing TNFα/IL-1β expression and NLRP3-mediated metabolic inflammation. This inflammatory signaling is a driving factor behind atherosclerotic plaque formation, insulin resistance, and the pathogenesis of obesity and NAFLD/NASH.^358–361^ Mitochondrial exposure to ceramides within hepatocytes and adipocytes inhibits oxidative phosphorylation, β-oxidation, and leads to mitochondrial fragmentation, a common finding in T2DM, obesity, and NAFLD/NASH.^362–365^ It is then incredibly clear that the relative ratio between gut FXR and central TGR5 agonism determines the rate and extent of ceramide synthesis, which directly induces the hallmark phenotypes associated with T2DM, obesity, NAFLD/NASH, and ASCVD.

### BA-mediated pathophysiology of diseases

#### BA-centric pathophysiology of T2DM

**Fig. 15:**
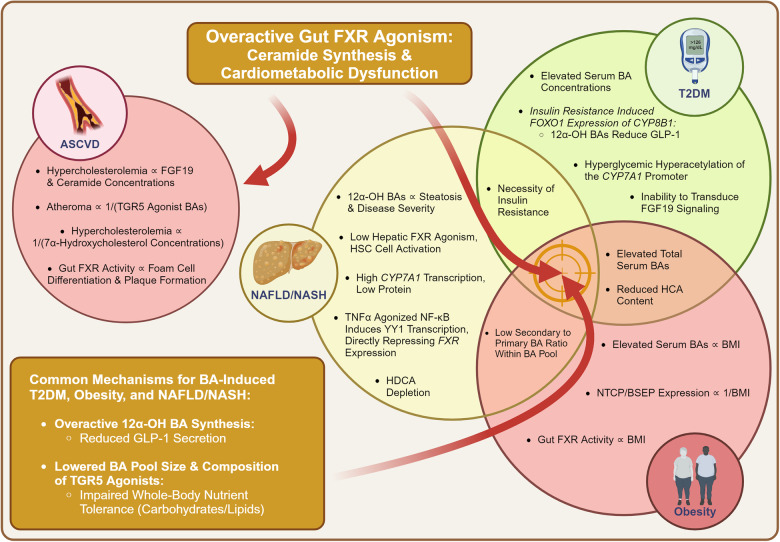
BA-centric pathogenic similarities between cardiometabolic diseases. Venn diagram displays the BA-centric phenotype and mechanisms of pathogenesis associated with each cardiometabolic disease. This figure was created with BioRender.com

Patients with T2DM appear to have elevated serum BA concentrations in both the fed/fasting states, irrespective of the intensity of insulin therapy (Fig. 15).^366–369^ This is thought to occur due to hyperglycemic-induced FXR-independent hyperacetylation of the *CYP7A1* promoter allowing for increased expression.^370^ In addition to an expanded BA pool size, BA-centric mechanisms reinforce the key T2DM characteristics of impaired insulin sensitivity and overactive gluconeogenesis. A direct connection can be made between BA signaling and T2DM by FOXO1, a gluconeogenic transcription factor that should be inhibited by insulin signaling, but in insulin resistance paradoxically remains active. FOXO1 enhances the expression of G6Pase, PEPCK, and in the context of BAs CYP8B1.^178,371^ In obese patients with insulin resistance higher ratios of serum 12α-OH BAs to non-12α-OH BAs are seen but normalizes in fully established T2DM.^372,373^ *CYP8B1*^−/−^ knockout mice were observed to have a lower hydroxylation ratio alongside with increased GLP-1 levels and improved insulin sensitivity, supporting the role that aberrant 12α-OH BA synthesis is associated with reduced incretin release. Overactive CYP8B1 activity leads to an increased BA pool fraction of CA, which increases the speed at which lipid nutrients are absorbed. This results in a smaller quantity of dietary fatty acids that reach enteroendocrine L-cells, lowering ATP generation and ATP-induced GLP-1 secretion.^374^

#### FXR-centric pathophysiology of T2DM

The main FXR-centric mechanism for T2DM pathogenesis is the role of overactive gut FXR agonism and the systemic secretion of ceramides. Gut FXR has a twin-fold nature in relation to gluconeogenesis. Gut FXR agonism favors gluconeogenesis by upregulating ceramide secretion and repressing GLP-1 secretion, while opposes gluconeogenesis by [FGF15/19]-[FGFR4/βK]-FXR axis mediated suppression of hepatic FOXO1/PGC-1α/HNF4α activity.^375^ Systemic ceramide exposure in CA-supplemented diet C57BL/6J mice directly predisposes for T2DM by inhibiting hepatocyte/adipocyte Akt leading to insulin resistance, by repressing the FXR-SHP-[HNF4α/FOXO1] axis, and by stimulating PC activity, leading to increased flux through all four rate-limiting enzymes of gluconeogenesis: G6Pase, PEPCK, FBPase, and PC.^176,376^ Although serum FGF19 levels are lowered in obese and T2DM patients, future research is needed to distinguish if analogous to insulin a FGF15/19 “resistance” exists, silencing either the secretion of FGF15/19 or its signal transduction within the liver.^377^ This may be plausible since both the insulin receptor and FGFR4 are receptor tyrosine kinases which are exposed to abnormal levels of their respective agonists. To provide further evidence, human trials in insulin-resistant and non-insulin-resistant NAFLD patients found an inability for insulin-resistant patients to successfully transduce FGF19 binding into *CYP7A1* repression.^378^ Collectively, decreases in incretin release by insulin resistance-induced 12α-OH BAs, ceramide-induced hepatocyte/adipocyte insulin resistance, and ceramide-induced overactivity of all four rate-limiting enzymes of gluconeogenesis create a strong connection between abnormal BA pool compositions, the gut-liver FXR-axis, and the T2DM phenotype.

#### TGR5-centric pathophysiology of T2DM

A lack of TGR5 signaling can influence both insulin resistance and the inflammatory responses common to the metabolic tissue of T2DM. In the gut, FXR agonism inhibits GLP-1 secretion while TGR5 agonism induces secretion.^174,259^ A relative increase in gut FXR agonism may lead to compromised GLP-1 release.^179,208,379^ This viewpoint is supported by *TGR5*^−/−^ mice which had impaired whole body insulin sensitivity and GLP-1 secretion.^380^ Low central TGR5 agonism is directly correlated to higher levels of T2DM-specific inflammation, leading to adipose/liver-associated insulin resistance by upregulating the NLRP3 inflammasome, increasing NF-κB/STAT3 signaling, and by decreasing the expression of anti-inflammatory LIP.^278,287,381,382^ This in addition to the lack of TGR5-induced increases to basal metabolic rate, ceramide metabolism, and TGR5-[PGC-1α]-ADIPOQ-AdipoR1/R2 mediated signaling, puts patients at risk for unregulated gluconeogenesis and insulin resistance. The importance of high gut TGR5 agonism and low gut FXR agonism in preventing the pathogenesis of T2DM is showcased by recent human studies on HCA, which both antagonizes gut FXR and agonizes gut TGR5. HCA is depleted in T2DM patients relative to healthy controls and directly reduces pre-prandial glucose measurements, indicating that its functional absence, an imbalance between gut FXR and gut TGR5 agonism, may be a key contributing factor to the T2DM phenotype.^383,384^

#### BA-centric pathophysiology of obesity

Serum BA concentrations in obese patients correlates positively with BMI (Fig. 15).^369,385^ Similar to obese patients with insulin resistance, patients who lose weight maintain the high 12α-OH BA to non-12α-OH BA ratio despite lowering total BA and GLP-1 concentrations to normal levels.^386^ Mechanistically the lack of proper non-12α-OH BA synthesis may be a long-term adaptive response to weight-gain that predisposes for further metabolic risk.^387–389^ In addition, human studies identified that hepatic BSEP/NTCP expression was negatively correlated with BMI.^390^ This indicates that obese patients have reduced hepatic efflux of primary BAs into the gallbladder, in addition to an impaired hepatic reuptake of conjugated secondary BAs from the gut. Subsequently, the hepatic primary BA overload and overactive FXR agonism would directly induce hepatic efflux of primary BAs into the systemic circulation. The lack of primary BA biliary transport will then hinder systemic rates of secondary BA synthesis, reducing the BA pool fraction of potent/efficacious TGR5 agonists.

#### FXR-centric pathophysiology of obesity

General FXR expression is protective against obesity due to maintaining lipid and carbohydrate metabolism, but overactive hepatic/gut FXR agonism has been found to exacerbate weight gain, dyslipidemias, and reduce glucose tolerance in HF diet ob/ob mice.^391–393^ Analyzing the pathophysiology of obesity, the strong negative autoregulation on BA synthesis may be an adaptive technique to reduce intestinal lipid absorption under a HF diet, but at the cost of reducing BA pool size and hence metabolic impacts at TGR5.^394^ Human gut FXR agonism within ileal biopsies has been found to directly correlate to BMI.^124^ Chronic gut FXR agonism and elevated serum ceramide concentrations are associated with diet-induced obesity (DIO) in both humans and mice.^358^ Gut FXR overactivity and ceramide-induced gluconeogenesis, lipogenesis, and lipid accumulation play key roles in the development of the obese phenotype.^124,395^ Gut-specific *FXR*^*−/−*^ mice were found to be protected against HF DIO and insulin resistance by either enhancing GLP-1 secretion and/or reducing negative ceramide-based metabolic effects.^83,124^ Whole-body *FXR*^*−/−*^ mice had increased HFD-induced dyslipidemias and glucose intolerance if fed a normal chow diet, but were protected against both obesity and glucose intolerance if fed a HF diet.^163,392,396,397^ The same results were not found in a liver-specific knockout, indicating that the protective role of whole-body FXR knockout may be due to gut-based repression of FXR.^398^ Analogous to that with T2DM, HCA prevalence was significantly lower in obese patients relative to healthy controls. Similarly, this indicates that the imbalance between gut FXR agonism and gut TGR5 agonism may be a crucial driving factor in the pathogenesis and maintenance of obesity.^383^

#### TGR5-centric pathophysiology of obesity

The role and proper agonism of TGR5 is paramount in preventing the onset of obesity.^399,400^ Mechanisms that shift BA pool composition away from TGR5 agonism may predispose and increase the risk for obesity. Experimentally it was identified that systemic knockout mice for βK (*Klb*^*−/−*^) were protected against HF DIO.^401^ The lack of βK was found to shift BA pool composition to favor the classical pathway to produce a higher fraction of DCA, a highly potent TGR5 agonist.^402^ Reduced TGR5 agonism would result in reduced basal metabolic rate and reduced incretin release, predisposing for obesity. This protective role was removed with double knockout *Klb*^*−/−*^/*TGR5*^*−/−*^ mice, insinuating that it is not purely the lack of FGFR4/βK activity that is protective, but the increase in potent/efficacious TGR5 agonizing BAs.^403,404^ Outside of traditional metabolic tissue, TGR5 has been found within the hypothalamus of mice. Within the hypothalamus TGR5 agonism promotes a systemic negative energy balance by increasing systemic sympathetic tone. Following from such, hypothalamic *TGR5*^*−/−*^ HF DIO mice were predisposed for obesity.^400^ Cumulatively, it is then apparent that changes to the BA pool size/composition that reduce TGR5 agonism have a direct effect on the pathogenesis of obesity.

#### BA-centric pathophysiology of NAFLD/NASH

Fasting and postprandial total/12α-OH BA levels of NASH patients correlate positively with steatosis and histological disease severity (Fig. 15).^405–408^ High transcription of *CYP7A1* but low CYP7A1 protein is exhibited in NAFLD/NASH, indicating faulty translation or overactive degradation of CYP7A1, along with the inability for hepatic FXR to suppress *CYP7A1*.^409,410^ Hepatic FXR activity is protective against steatosis and fibrosis, the two main hallmarks of NAFLD and its disease progression into NASH or hepatocellular carcinoma (HCC).^398,411–414^ Whole-body *FXR*^*−/−*^ in mice is associated with increased hepatic levels of cholesterol, free fatty acids, triglycerides, and inflammation, where lipid accumulation is inversely correlated with FXR expression in both mice and humans.^411,415–417^ Intestinal *FXR*^*−/−*^ in mice improves HF diet-induced steatosis.^83,124,132^ This is accomplished by reducing ceramide-induced hepatic FXR repression, allowing for proper FXR-SHP axis SREBP-1C silencing. Further supporting this hypothesis is the activity of HDCA, which decreases triglyceride concentrations by activating PPARα-induced β-oxidation and inhibits gut FXR ceramide-induced steatosis. HDCA was significantly depleted in both NAFLD patients and HF high sucrose NAFLD model mice relative to healthy controls, indicating that the lack of BA PPARα agonism and gut FXR antagonism may be pathogenic for NAFLD/NASH.^347,418^

#### FXR/TGR5-centric pathophysiology of NAFLD/NASH

The fibrotic risk for a NAFLD/NASH patient is positively correlated to both high conjugated CA concentrations and lower ratios of secondary to primary BAs.^408,419^ Analogous to obesity, this may be caused by similar inhibition of BSEP/NTCP. Phenotypically this may present with hepatic efflux of primary conjugated BAs into the systemic circulation and reduced entry of BAs into the gut, hence lower rates of secondary BA synthesis. The key cells responsible for hepatic scarring and fibrosis are hepatic stellate cells (HSC), which when agonized by the FXR-SHP axis exhibit less fibrotic activity.^420–422^ HSC FXR-SHP axis agonism represses the expression of and responsiveness to transforming growth factor beta (TGFβ) and its receptor transforming growth factor beta receptor 2 (TGFβR2) in CCl_4_ mice fibrosis models.^423,424^ In addition, agonism increases the expression of Perilipin-1 (PLIN1) to stabilize fat droplets and induces Peroxisome Proliferator-Activated Receptor-Gamma (PPARγ) to reduce inflammatory cytokine and collagen synthesis.^425,426^ However, in situations of HSC activation, such as in NASH, it becomes more challenging to activate FXR due to increasing rates of SUMOylation.^427^ *FXR*^*−/−*^ mice exhibited enhanced hepatic inflammation and necrosis from lipopolysaccharide (LPS) administration or autoimmune hepatitis, highlighting the crucial role of FXR in reducing hepatic inflammatory responses.^188,193,428^ Further evidence supports the role of the FXR-SHP axis in preventing the inflammatory phenotype of NAFLD/NASH, in which FGF15/19 supplementation prevents hepatic inflammation and cholangiopathies in HF, high fructose, and HC diet NASH mouse models.^429,430^

TGR5 agonism in relation to NAFLD/NASH is nuanced. Intestinal TGR5 agonism protects against hepatic steatosis by GLP-1 secretion and adipose TGR5 agonism protects against lipotoxicity by ADIPOQ signaling. Systemic TGR5 agonism is potently anti-inflammatory and inhibits NF-κB activity in mouse models of hepatic LPS-induced inflammation, likely reducing hepatic inflammatory burden.^280^ TGR5’s hepatic immunological response likely plays a strong role in NAFLD/NASH pathogenesis, in which its activity in Kupfer cells directly prevents LPS-induced cytokine production.^431^ The lack of TGR5 agonism appears to be pathogenic for NAFLD, while TGR5 agonism may be pathogenic for disease progression into NASH, in which TGR5 induces p38MAPK/ERK1/2 activity within HSCs and TGFβ synthesis within mice macrophages, driving profibrotic activity.^284,432,433^

#### Gut-liver axis dysfunction suppresses hepatic FXR and induces NAFLD/NASH

NAFLD pathogenesis and disease progression to a good approximation occurs according to the “two-hit” hypothesis: hepatic lipid accumulation followed by inflammation-induced fibrosis.^434–436^ The activity of hepatic FXR is critical for protection against both steatosis and inflammation-induced fibrosis and was found to be repressed by the protein Ying Yang 1 (YY1).^437^ YY1 is a transcriptional repressor that has roles in cell-cycle progression, mitogenesis, and insulin/insulin-like growth factor (IGF) signaling, but most importantly strongly represses *FXR*.^438–441^ Mechanistically, activated YY1 binds intron-1 of *FXR* and prevents transcription. YY1 was found to be upregulated in the livers of HF DIO mice, db/db mice, and NAFLD patients due to TNFα-induced activation of NF-κB and NF-κB stimulated transcription of YY1.^442–444^ TNFα exposure may be endogenous due to obesity-induced inflammation or exogenous via c exposure.^445,446^ Anti-YY1 shRNA containing adenovirus infection was found to rescue FXR levels and decrease hepatic steatosis in obese mice.^447^ The activity of YY1 hints towards a threshold in which a baseline of obesity-induced inflammation triggers the repression of hepatic FXR, eliminating its important role in protecting against both unregulated SREBP-1C lipogenesis and the activation of profibrotic HSCs; the lack of proper hepatic FXR agonism and overactive gut FXR agonism are both directly associated with the disease onset of NAFLD/NASH. Cumulatively, the proper expression and function of hepatic FXR and repression of gut FXR appear to be key factors in the development of NAFLD/NASH.

#### BA-centric pathophysiology of cardiovascular disease

BAs have dose-dependent effects on the heart, which if elevated may be cardiotoxic and lead to cardiomyopathy.^448^ To expand on such, inhibition of FXR has been found to be protective against ischemic cardiac damage, while FXR agonism is pro-apoptotic in cardiomyocytes.^449^ From the perspective of cardiac arrythmias, conjugated BAs such as taurine conjugated CA have been found to contribute towards atrial arrythmias such as atrial fibrillation.^450^ Similar to other diseases, imbalances to BA pool size and composition are likely to play key roles in the pathogenesis of cardiomyopathic diseases such as heart failure and atrial arrythmias.

It is still largely unknown how potent systemic FXR activity is in modulating atherosclerosis. Some evidence exists supporting that CDCA is a 3-Hydroxy-3-Methyl-Glutaryl-Coenzyme A (HMG-CoA) Reductase inhibitor.^451^ Double knockout *FXR*^*−/−*^ *ApoE*^*−/−*^ mice fed a HF diet were found to have increased atherosclerotic lesion sizes and abnormal plasma lipid profiles.^452^ Furthermore, FXR signaling within vascular smooth muscle was found to blunt myocyte migration and the inflammatory response associated with atherosclerotic plaque development.^453^ Mechanistically, this occurs due to the FXR-SHP axis mediated repression of inducible nitric oxide synthase and cyclooxygenase-2.^454^ Although it may seem that FXR agonism definitively improves atherosclerosis, somewhat contrasting work has been published showing opposite net effects. *FXR*^*−/−*^ in *LDLr*^*−/−*^ or *ApoE*^*−/−*^ mice reduced atherogenic lesion size and produced unexplained plasma lipid differences.^455,456^ Similar results have been found in humans, in which systemic FXR agonists like OCA increase serum LDL cholesterol in a dose-responsive relationship.^457^

It is important to remember that FXR has time-, dose-, and tissue-dependent impacts on lipid physiology. Within humans, hypercholesterolemia is positively correlated with circulating FGF19/ceramide concentrations and inversely correlated with 7α-hydroxycholesterol concentrations. This indicates that a relationship exists between cholesterol burden and gut FXR agonism (Fig. 15). Gut FXR-mediated ceramide synthesis has negative impacts on the cardiovascular system in HC fed mice, directly inducing macrophage to foam cell differentiation and NLRP3 initiation of plaque inflammation, driving further plaque development. Furthermore, gut FXR agonism in HC diet mice via FGF15 secretion represses hepatic *CYP7A1*, *LDLr*, and *ABCG8* expression, but paradoxically has no activity on inducing gut *ABCG5*/*ABCG8* expression, further driving hypercholesterolemia. Gut *FXR*^*−/−*^ mice exhibited significantly reduced ASCVD development and SMPD3-mediated ceramide synthesis, which was reversed in gut *FXR*^*−/−*^ mice that overexpressed SMPD3 via a lentivirus vector.^358,359^ It is likely that increased hepatic cholesterol burden increases BA synthetic flux, providing more gut FXR agonism and FGF15-mediated *CYP7A1* repression. Repression of *CYP7A1* biases BA pool composition towards the potent FXR agonist CDCA, further agonizing gut FXR and forming a positive feedback loop. Persistent gut FXR agonism enhances hepatic cholesterol burden, while ceramide expression directly accelerates atherosclerotic plaque development.

TGR5 is not without cardiovascular concern, in which 3,5-diethoxycarbonyl-1,4-dihydrocollidine-induced mouse models of biliary fibrosis have developed cardiac hypertrophy due to overactive TGR5-Akt signaling.^458^ In dogs TGR5 agonists have been found to induce reflex tachycardia due to TGR5-induced activation of the K(Ca)1.1 potassium leak channel within vascular smooth muscle.^459^ Contrasting results have been shown in other studies, in which TGR5 is cardioprotective by reducing cardiac stress and by reducing atherosclerosis.^279,460^ In human patients it was found that LCA levels negatively correlated with the presence of atheroma, highlighting the potential pro-atherosclerotic phenotype of a low LCA content BA pool.^461^ Further research is needed to pinpoint the positive and negative roles of TGR5 on cardiovascular physiology.

#### BA-centric pathophysiology IBDs

Mucosal immune cells within the gut must defend against pathogens, retain an endogenous balance of commensal bacteria, and prevent disruption of the gut mucosal barrier.^462^ BA pool size and composition may directly modulate the gut micro-environment. Millimolar concentrations of conjugated primary BAs are antimicrobial by triggering FXR agonism-induced defensin responses, assisting to prevent bacterial overgrowth.^463–465^ In some cases, BA-mediated effects have been found to be protective against opportunistic pathogens such as *Clostridium difficile*.^466,467^

DSS and 2,4,6-trinitrobenzene sulfonic acid (TNBS) mouse models of colitis have found that the extent of systemic FXR agonism impacting monocytes, macrophages, dendritic cells, and gut epithelia is inversely proportional to mucosal inflammation.^468,469^ A similar phenotype was found in two different rat models of intestinal inflammation, bile duct ligated cholestasis and mesenteric artery clamp induced reperfusion injury.^470,471^ From such it is believed that FXR represses mucosal inflammation by modulating both the innate immune system and intestinal epithelia, likely by inhibiting the NF-κB/NLRP3 induced expression of TNFα, IL-1β, and IL-6. In humans it was found that patients with colitis had decreased intestinal expression of FXR, while in mice FXR activation reduced colitis disease severity.^101,191,472^ Collectively, low gut FXR activity may be pathogenic for IBD.

TGR5 agonism modulates the integrity and inflammatory status of intestinal mucosa in DSS/TNBS rodent models of colitis.^473^ TGR5 activation was able to directly suppress LPS-induced inflammatory cytokine production in macrophages derived from patients with Crohn’s disease.^282^ Treatment of mucosal macrophages derived from Crohn’s disease patient biopsies with a TGR5 agonist directly reduced pro-inflammatory cytokine expression, supporting that situations of low TGR5 agonist concentration within the BA pool may be pathogenic towards IBD.^282^ TGR5 agonism additionally biases the differentiation of human monocytes into tolerogenic dendritic cells, secreting low levels of TNFα and IL-12.^474^ Lastly, TGR5 induced IL-10 expression has been found to increase the recruitment of anti-inflammatory peripheral regulatory T cells (pTregs) to inflamed colonic tissue.^284^

#### BA metabolites modulate IBD pathogenesis by influencing pTreg/Th17 differentiation

**Fig. 16:**
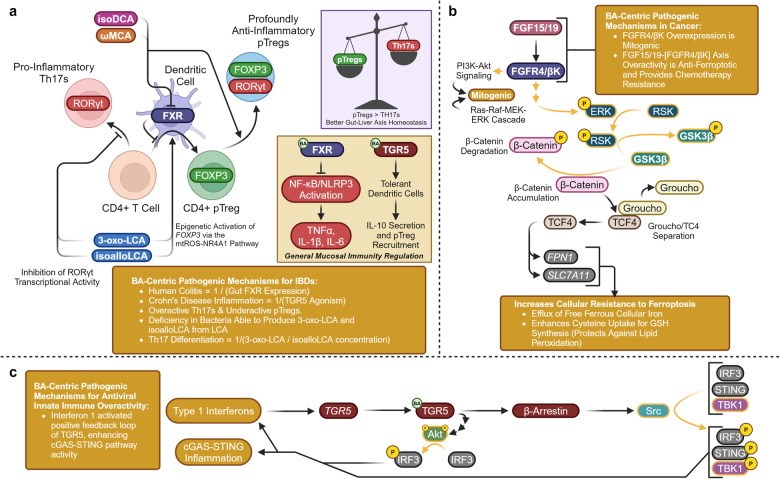
BA-centric pathogenic mechanisms for IBD, cancer, and antiviral innate immune overactivity. **a** Novel BA metabolites enhance pTreg differentiation and inhibit Th17 differentiation, reducing autoimmune IBD-associated inflammation and enhancing gut-liver axis homeostasis. **b** The FGF15/19-[FGFR4/βk] axis can predispose for neoplastic promotion by inducing mitogenesis. In addition, the FGF15/19-[FGFR4/βk] axis can predispose for chemotherapeutic resistance by preventing chemotherapy-associated ferroptosis. **c** The Type 1 Interferon response induces TGR5-mediated activation of the cGAS-STING signaling cascade, enhancing antiviral responses, and potentially enhancing cGAS-STING-associated inflammation. Gold border proteins represent kinases, while gold arrows represent phosphorylation. mt is an abbreviation for “mitochondrial”. This figure was created with BioRender.com

Control of the immune response to bacteria is crucial for proper barrier function (Fig. 16a). Secondary BAs like *Ruminococcus gnavus* 3α/β-HSDH C_3_-epimerized DCA (isoDCA) and ωMCA antagonize FXR transcriptional activity within the dendritic cells of mice, inducing the differentiation of pTregs dampening immune responses.^189,475^ This effect remained without ligand administration within *FXR*^*−/−*^ mice dendritic cells, pinpointing the outcome as a direct endpoint of FXR antagonism. Cluster of Differentiation 4 (CD4) positive (CD4^+^) T cells are directly differentiated into pTregs by expression of the Forkhead Box P3 (FOXP3) transcription factor. On its own, expression of the Retinoid Orphan Receptor Gamma T (RORγt) transcription factor induces the differentiation of CD4^+^ T cells into pro-inflammatory T Helper 17 (Th17) cells, while its activation after that of FOXP3 produces pTregs with enhanced immunosuppressive abilities.^189,476–478^

The administration of isoDCA does not only induce pTreg differentiation but increases the number of FOXP3^+^/RORγt^+^ co-expressed Tregs, indicating enhanced anti-inflammatory activity. In addition to the role of isoDCA in producing pTregs, secondary BA VDR agonists have been found to enhance the differentiation of FOXP3^+^/RORγt^+^ Tregs.^479^ Seemingly contradictory in nature, it is still mechanistically unknown why FXR antagonists like isoDCA, ωMCA, and not UDCA provide pTreg induction when it is FXR agonism that induces well-characterized dendritic cell anti-inflammatory phenotypes. As a caveat, these in vitro experiments were co-cultures of T cells and dendritic cells, so it is still unknown if FXR antagonism at dendritic cells at the in vivo scale is more important for IBD pathogenesis compared to well-known potent general gut anti-inflammatory FXR agonism.

Alongside the FXR-sensitive impact of isoDCA and ωMCA on dendritic cell-induced pTreg differentiation, other secondary BA metabolites play extensive roles in directly modifying CD4^+^ differentiation into Th17s/pTregs, such as 3-oxo-LCA and the C_3_/C_5_ epimer of LCA (isoalloLCA). LCA is metabolized by microbial 3α-HSDH to produce 3-oxo-LCA, which inhibits Th17 differentiation by directly binding to and inhibiting the transcription factor RORγt. *Bacteroidete* phylum 5α/β-reductase and 3β-HSDH activity produces isoalloLCA from 3-oxo-LCA, which when exposed to CD4^+^ T cells induces mitochondrial reactive oxygen species (ROS) generation. This ROS by unknown mechanisms relaxes the chromatin of *FOXP3*, allowing for Nuclear Receptor Subfamily 4 Group A Member 1 (NR4A1) to bind and enhance *FOXP3* transcription, producing FOXP3 and differentiating CD4^+^ T cells into pTregs.^480^

To further the importance of BAs in gut immune regulation, 3-oxo-LCA, isoalloLCA, and the bacterial microbiome prevalence of their required metabolic enzymes are all found to be significantly deficient in patients with IBDs, in which their concentrations are inversely correlated with the expression of human Th17-associated genes.^481^ Alongside this, human metagenomic studies have found that patients with IBDs have lower quantities of secondary BAs.^482^ Cumulatively, this indicates that a lack of both secondary BA substrates and their required metabolic enzymes may provide a deficiency of 3-oxo-LCA- and isoalloLCA-mediated immunosuppression, being pathogenic for IBDs.^483,484^ From a structural perspective, these three ligands have either planar or β-orientation C_3_ functional groups, perhaps serving as a basis for new structure-activity relationships investigating BA-mechanistic immunosuppressive therapies.

#### A bidirectional inflammatory highway, the gut-liver axis

The gut-liver axis holds a central role in BA physiology, in which hepatic function is bidirectionally impacted by the gut in terms of both human tissue and the microbiome. If the liver does not produce a substantial enough BA pool of the correct composition, both gut inflammation and overall bacterial burden increase. This negative hepatic-induced gut phenotype decreases the integrity and increases permeability of the mucosal barrier, enhancing hepatic exposure to bacterial toxins such as LPS, which are now able in higher quantities to enter hepatic portal circulation and trigger hepatic inflammation.^485,486^ Hepatic inflammation may then induce phenotypes associated with NAFLD/NASH and further detriment BA-regulated gut health.^487,488^ To illustrate this point, a deficiency in PXR agonizing BAs has been shown to increase the prevalence of *BSH* containing *Lactobacillus*. Although this outcome may modulate FXR-induced defensin production, PXR agonism deficiency decreases mucosal barrier function by enhancing Toll-Like Receptor 4 (TLR4) mediated inflammatory signaling, likely enhancing LPS burden on the liver.^489,490^

#### BA-centric pathophysiology of cancer

Primary and secondary BAs have been widely associated with carcinogenesis and positive or negative impacts dependent on the concentration administered and the type of neoplasm.^491^ This heterogeneity in outcome is likely driven by the tissue-dependent extent and selection of BA receptor expression and the again tissue-dependent outcome of specific receptors. Nuclear BA receptors such as FXR, PXR, LXR, CAR, VDR, membrane receptors such as TGR5, SIPR2, CHRM2, and CHARM3, and BA-adjacent receptors such as FGFR4/βK influence many downstream pathways commonly associated with neoplastic development such as EGFR, MAPK, STAT3, NF-κB, TLR4, HNF4α, β-catenin, Suppressor of Cytokine Signaling 3 (SOCS3), and in the case of FGFR4/βK, directly FGFR4.^492–497^

The impact of BAs on carcinogenesis is concentration dependent, in which low concentrations are anti-cancerous and high concentrations are carcinogenic. This ambivalence is likely due to their amphipathic nature and from such polypharmacology, which at lower doses is likely inactive but at supraphysiological doses may act as a surfactant damaging membranes to trigger Phospholipase A1 (PLA2)-mediated inflammation and ROS, while PKC activation mediates p38-MAPK-p53-[NF-κB] signaling, apoptosis, and inflammation.^466,498–500^ The inherent cytotoxicity of high-dose BA exposure is supported by the numerous endogenous physiological mechanisms in place to detoxify high levels of lipophilic BAs.

Out of all BAs, UDCA appears to be the most fit as a tumor-suppressive therapy. Throughout various concentrations ranging from 100 μM to 800 μM, UDCA has been found to be tumor suppressive in many different cancers, such as hepatocellular carcinoma, gastric cancer, colon cancer, cholangiocarcinoma, prostate cancer, and glioblastoma.^501–510^ LCA from 0.3 μM to 200 μM, DCA from 10 μM to >500 μM, and CDCA from 10 μM to >500 μM have been found to exert pro-apoptotic and anti-proliferative signaling in multiple different cancers such as breast cancer, colorectal cancer, gastric cancer, hepatocellular carcinoma, ovarian cancer, and prostate cancer.^497,511–517^ At the same time, LCA, DCA, and CDCA have been found to be pro-carcinogenic in similar concentration ranges, enhancing cholangiocarcinoma, colorectal cancer, gastric cancer, hepatocellular carcinoma, Barett’s esophagus, along with many other cancers.^303,493,518–528^ This further establishes that finely-tuned research is needed to pinpoint tissue-dependent specific thresholds in which certain BAs switch from primarily anti-carcinogenic to pro-carcinogenic pharmacodynamic profiles.

#### FGFR4 overexpression is carcinogenic and provides chemotherapeutic resistance

The FGF15/19-[FGFR4/βK] axis is highly associated with both carcinogenesis and chemotherapeutic resistance (Fig. 16b). FGF15/19-[FGFR4/βK] axis activity directly activates mitogenic signaling pathways within hepatocytes, such as PI3K-Akt and Ras-Raf-MEK-ERK, being strongly associated with the promotion of human hepatocellular carcinoma.^529,530^ Alongside the direct promotion of hepatocellular carcinoma, FGFR4/βK prevents ferroptosis within human hepatocytes and breast tissue. Mechanistically, FGF15/19-[FGFR4/βK] axis signaling induces ERK-RSK phosphorylation and inhibition of GSK3β. Inhibition of GSK3β prevents its activity within the β-catenin destruction complex, preventing β-catenin phosphorylation/ubiquitination, and allowing for its accumulation. β-catenin upon displacing the protein Groucho from Transcription Factor 4 (TCF4) within the nucleus activates TCF4 and induces the expression of Solute Carrier Family 7 Member 11 (SLC7A11) and Ferroportin 1 (FPN1).^531,532^ Both of these proteins are anti-ferroptotic. FPN1 directly effluxes reactive ferrous iron while SLC7A11 exchanges extracellular cysteine for intracellular glutamate, maintaining intracellular glutathione stores and ensuring the activity of Glutathione Peroxidase 4 (GPX4)-mediated detoxification of iron-induced lipid peroxidation.^533,534^ If overactive this pathway may strongly repress the ability for cytotoxic agents to kill tumors, acting as a major mechanism of chemotherapeutic resistance for pan-kinase inhibitors such as Lenvatinib.^535^

Besides that of the FGF15/19-FGFR4/βK axis, FXR and TGR5 activity have been found to reduce carcinogenesis. *FXR*^*−/−*^ mice have significantly higher rates of hepatic cancer.^413,417^ In addition, TGR5 by unknown mechanisms antagonizes the activity of Signal Transducer and Activator of Transcription 3 (STAT3) signaling pathways, resulting in suppressed Lipopolysaccharide (LPS) or IL-6 induced proliferation of liver, gastric, colorectal, and breast cancer.^281,536^

#### BA receptor-mediated contributions to the antiviral innate immune response

BA activity at TGR5 plays a significant role within the innate antiviral response (Fig. 16c). The type 1 interferon response associated with viral infections directly induces the expression of TGR5. From such, TGR5 agonism participates in a positive feedback loop, upregulating Akt/Interferon Regulatory Protein 3 (IRF3) signaling for further type 1 interferon synthesis and antiviral responses.^537^ In addition, TGR5 induced β-Arrestin activity has been found to activate Src, providing interplay between the FXR and TGR5 signaling cascades and in this case further upregulating antiviral responses of the innate immune system by phosphorylating Stimulator of Interferon Genes (STING), Tank-Binding Kinase 1 (TBK1), and IRF3, collectively upregulating the latter portion of the cGAS-STING pathway.^538^ This is especially interesting since recent research on the cGAS-STING pathway has indicated its overactivity in the pathogenesis of age-related inflammatory, neurodegenerative, and autoimmune diseases.^539,540^ Therefore, changes to the BA pool composition may directly enhance or repress overactive cGAS-STING pathway activity and its associated pathogenic effects.

## Druggable targets, BA pharmacology

### FXR modulators

#### Steroidal FXR modulators

**Fig. 17:**
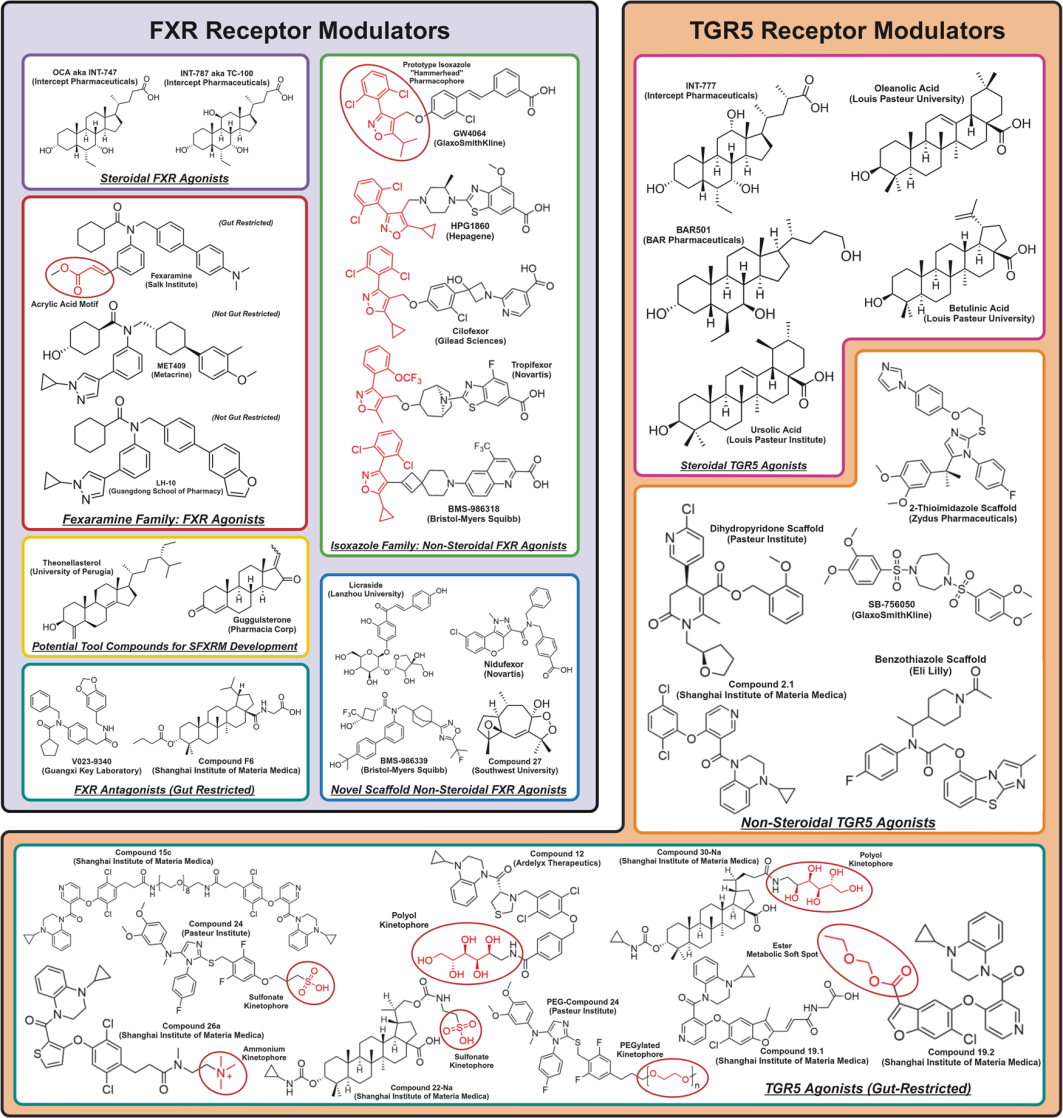
Structures of key FXR and TGR5 modulators. Red circles are drawn around the following key structural motifs: non-steroidal FXR agonist isoxazole pharmacophore, the acrylic acid motif of fexaramine likely to provide gut-selectivity, various kinetophores allowing for TGR5 gut-selectivity, and an example of a metabolic soft spot again providing gut-selective TGR5 agonism. This figure was created with BioRender.com

Alongside endogenous BA FXR modulators, semisynthetic steroidal modulators of FXR have been synthesized, the most famous of which is OCA also known as INT-747 (Intercept Pharmaceuticals) (Fig. 17). OCA is structurally 6α-ethyl-CDCA and touts an impressive 100-fold increase in receptor potency compared to endogenous CDCA.^541,542^ A further modified derivative of OCA known as EDP-305 (Enanta Pharmaceuticals) is ~16-fold more potent than OCA and shows no activity at TGR5.^543^ Another derivative of OCA, TC-100, is C_11_β-OH OCA, a fully selective FXR agonist. TC-100 is roughly 16-fold more water soluble compared to OCA, limiting hydrophobic BA-induced toxicity, and ensuring rapid excretion from hepatocytes, likely exerting more gut-centric agonism.^544^ MFA-1 (Merck) was discovered as a steroidal FXR agonist but binds inverted in orientation to FXR compared to endogenous BAs. No transcriptional data for this has been published.^545^ Lastly, the betulinic acid derivative Compound F6 (Shanghai Institute of Materia Medica) was discovered as a potent gut-restricted FXR antagonist.^546^

#### Non-steroidal FXR modulators

The discovery and development of non-steroidal FXR agonists was a direct attempt to eliminate some of the adverse effect profile associated with steroidal FXR agonists, ideally eliminating pruritis and a pro-atherogenic serum profile.^547^ In general, drugs with a steroidal scaffold have the risk of binding to other steroid receptors (glucocorticoid, mineralocorticoid, androgen, estrogen, progesterone), from such deriving some of their adverse effects. Many believed that a FXR-selective non-steroidal scaffold could improve pruritis, since it was known that steroidal scaffolds could bind to TGR5 on dorsal root ganglionic neurons and release itch neuropeptides.^548^ There was less enthusiasm for a non-steroidal solution against the induction of a pro-atherogenic lipid profile, since it was mechanistically explainable as an FXR-intrinsic outcome.

Most non-steroidal FXR agonists are within the isoxazole “hammerhead” family, originally derived from GW4064 (GlaxoSmithKline).^549^ GW4064 has a low bioavailability, tropism for the Estrogen Receptor-related Receptor Alpha (ERRα), and has a short half-life.^550^ The short half-life and low bioavailability is general for the hammerhead class of FXR agonists, which are structurally similar enough to endogenous BAs to be hepatically taurine conjugated by BACS and BAT. Before conjugation, hammerhead family FXR agonists are ligands for ASBT-mediated gut absorption, but once conjugated are not, reducing their bioavailability by inhibiting enterohepatic circulation.^551^

Many compounds synthesized have been isoxazole-bearing derivatives of GW4064, such as the full agonists tropifexor (Novartis) and cilofexor (Gilead Sciences).^552–556^ Although abandoned, PX-102 (Gilead Sciences) was the structural intermediate between GW4064 and cilofexor (Gilead Sciences).^551,557,558^ Of this same class Novartis has the partial agonist nidufexor (also known as LMB763).^559^ TERN-101 (Terns Pharmaceuticals) was similarly discovered but instead as a partial agonist.^560,561^ Other isoxazole FXR agonists such as BMS-986318 (Bristol Myers Squibb) and the more recent BMS-986339 (Bristol Myers Squibb) have been discovered as high potency FXR agonists.^562,563^ The last major molecule of the isoxazole family is HPG1860 (Hepagene Therapeutics).^564^

Besides that of GW4064 derivative scaffolds, WAY-362450 (Wyeth Pharmaceuticals which was acquired by Pfizer) is a potent full FXR agonist but is horribly insoluble and has been tested only in mice.^565,566^ In 2003 fexaramine (Salk Institute) was discovered, which exhibited partial gut FXR agonism and no significant hepatic agonism. Fexaramine has very poor oral bioavailability, indicated by a single dose oral vs. intraperitoneal relative ~10% bioavailability in mice, establishing fexaramine as the prototype for gut-restricted FXR agonists.^551,567,568^ No clinical data has been published on fexaramine, however, the systemically active derivative of fexaramine MET409 (Metacrine) has been tested in humans against NASH and obtains serum concentrations high enough to trigger robust hepatic FXR agonism.^569^ Structure activity relationships have found that many of the general modifications performed on fexaramine to produce MET409 retain gut-restriction, however it is hypothesized that removing the Michael acceptor acrylic acid motif is what removes gut-restriction.^551,570^ This indicates that fexaramine may be gut-restricted due to covalent adduct formation within intestinal epithelia. This is corroborated by the recent discovery of the systemic FXR agonist LH-10 (Guangdong School of Pharmacy), which is another fexaramine derivative that is systemically active after removing the acrylic acid motif.^571^

Two recent developments have occurred in non-steroidal FXR modulator synthesis, namely the creation of a gut-restricted FXR antagonist and the identification of new natural product non-steroidal FXR agonist scaffolds. The 4-aminophenylacetamide scaffold V023-9340 (Guangxi Key Laboratory) was found to be potent gut-restricted FXR antagonist without significant hepatic suppression.^572^ Lastly, the sesquiterpenoid Compound 27 (Southwest University) and the glucoside flavonoid licraside (Lanzhou University) have been found to be novel scaffold non-steroidal FXR agonists.^573,574^

#### Key preclinical results—systemic FXR modulators

Systemic FXR agonists have emerged as potent but temperamental therapeutics. The endogenous weak FXR agonist CA was found to repress triglyceride levels in KK-A^y^ mice models, dampening hepatic steatosis and NAFLD development.^150^ OCA in Zucker fa/fa obese rats was found to reverse insulin resistance, correct obesity-induced lipid abnormalities, protect against weight gain, and was protective against hepatic steatosis.^173^ If treated with the FXR agonist OCA, the colons of DSS-induced colitis mice were found to have lower levels of the pro-inflammatory cytokines IL-1β and IL-6.^469^ In addition, OCA repressed TLR4-induced pro-inflammatory activity within intestinal epithelial cells, while reducing inflammatory cytokines expression in cultured human CD14^+^ monocytes and dendritic cells.^191,469^

GW4064, WAY-362450, PX-102, tropifexor, nidufexor, and cilofexor are also potent in treating cardiometabolic diseases. GW4064 was found to repress gluconeogenesis and lipogenesis within a db/db diabetic mice model.^170^ However, long-term dosing of GW4064 in obese insulin-resistant mice due to overactive FXR activity led to exacerbated weight gain and further worsened insulin resistance.^391^ GW4064 was also found to improve cholestatic hepatotoxicity in rats and reperfusion damage by upregulating SHP in Kupfer cells.^575,576^ WAY-362450 prevented fructose-induced hepatic steatosis in C57BL/6J mice and protected against NASH onset for mice fed a methionine and choline-deficient diet.^577,578^ WAY-362450 administration had a protective role against atherosclerotic lesion formation in *ApoE*^*−/−*^ or *LDLr*^*−/−*^ mice.^190,579^ Administration of tropifexor, nidufexor, and cilofexor have all been found to improve NASH symptoms in choline-deficient HF diet, chemical/dietary, and insulin-resistant obese NASH models in rodents.^559,580,581^ OCA, cilofexor, and a non-steroidal tool-compound named INT-2228 (Intercept Pharmaceuticals) had comparable impacts on serum LDL/HDL cholesterol levels in chimeric PXB-mice, reproducing the phenotype of heightened LDL cholesterol and lowered HDL cholesterol.^582^ BMS-986318 and BMS-986339 have both been found to strongly activate FXR-mediated antifibrotic effects in mice bile duct ligation NASH models.^562,563^ LH-10, licraside, and Compound 27 were all found to ameliorate α-napthylisothiocyanate (ANIT) mice model cholestasis.^571,573,574^ HPG1860 was found to prevent CCl_4_-induced NASH model inflammation, steatosis, ballooning, and fibrosis similar in effect to OCA.^564^

#### Key preclinical results—gut-restricted FXR modulators

Fexaramine in DIO mice reduced diet-induced weight gain, insulin resistance, fasting serum total cholesterol/triglycerides, serum TNFα/IL-1β, gluconeogenesis, and enhanced basal metabolic rate within adipose tissue. Mechanistically, fexaramine activates gut FXR-induced expression of FGF15/19, which represses *CYP7A1*, shunting cholesterol into the alternative pathway of synthesis.^151,567,583,584^ Enhanced BA pool composition of CDCA is metabolized by fexaramine-induced increases to *BSH* and *bai* operon containing bacteria. The net effect of this is a smaller total BA pool with a higher composition of LCA, enhancing TGR5 agonism and TGR5-induced GLP-1 release.^568,585^

In addition to fexaramine’s impact to BA pool composition, *TGR5*^*−/−*^ mice models have found that the changes in weight gain, insulin sensitivity, and triglycerides may be due to FXR-dependent interplay with TGR5 signaling. Besides altering BA pool composition, this outcome is likely accomplished within adipose tissue and the liver by the action of the [FGF15/19]-[FGFR4/βK]-FXR axis on the FXRE found within the *TGR5* promoter, increasing spare receptors and hence total BA pool TGR5 agonist potency.^586^ The TGR5-dependent ability of fexaramine to repress gluconeogenesis and lipogenesis hints that fexaramine may be able to induce ceramide metabolism. Fexaramine was able to induce DIO2 and PGC-1α expression in the same *TGR5*^−/−^ model, highlighting unknown mechanisms in which FXR can mimic the phenotype typically seen with TGR5 agonists. This may be explainable by fexaramine-induced increases to BA pool LCA composition, likely triggering the effects of non-canonical BA receptors.^568^

Alongside the success of fexaramine, the direct administration of gut FXR-antagonistic BAs such as taurine/glycine conjugated βMCA and UDCA have been shown to prevent and reverse HF DIO, insulin resistance, hyperglycemia, and steatosis in mice. Three different experiments in mice have been performed using either gut-specific *FXR*^*−/−*^ or taurine/glycine conjugated βMCA and have confirmed that the metabolic benefit is directly related to gut FXR inhibition of ceramide secretion.^83,124,132^ Conjugated UDCA administration through a ceramide-centric mechanism was found to strongly repress atherosclerotic plaque development in *ApoE*^*−/−*^ mice.^358^ Intragastric administration of UDCA has been found to reduce NASH-associated hepatic inflammation and restore gut dysbiosis in a HF HC mice NASH model.^587^ V023-9340 and Compound F6 have both been found to improve metabolic outcomes in HF diet and Gubra-amylin mice NASH models.^546,572^ Compound F6 activity was directly tied to inhibiting intestinal ceramide synthesis.

#### Clinical results—FXR modulators

CA supplementation with 15 mg/kg was not found to reduce hepatic triglyceride content in humans.^588^ CDCA was found to accelerate colonic transit and improve bowel function in patients with constipation-predominant irritable bowel syndrome.^589^ EDP-305 was involved in two phase 2 studies, one for PBC and the other for NASH. For PBC EDP-305 was able to lower alkaline phosphatase, while for NASH it reduced serum alanine transaminase levels and hepatic lipid content.^543,590^ In addition, EDP-305 increased serum LDL cholesterol and caused pruritis. As a proof of concept, PX-102 was found in small human trials to repress BA synthesis and increase FGF19 secretion.^557^ Two phase 1 trials have been conducted for single and multiple ascending doses, however the results of such have not been published as the compound has been abandoned.^591^ WAY-362450 was involved in two phase 1 trials, one of which was terminated due to pharmacokinetic issues, while the other completed, both of which had no published results.^592,593^

Tropifexor was primarily designed as an anti-NASH therapeutic and has completed a phase I safety trial.^552,594^ Recently a phase IIa/b trial FLIGHT-FXR was completed in which decreases in alanine transaminase levels, decreased hepatic fat fraction, increased weight loss, and improved surrogate markers for NASH improvement but not actual histological disease improvement were observed.^594,595^ In addition, tropifexor was found to increase serum LDL cholesterol and cause pruritis. Secondary trials have found that tropifexor was able to improve cholestatic biomarkers and potentially serve as a drug for PBC.^596^ Nidufexor was designed as an anti-NASH drug but has been studied specifically in patients with NASH and diabetic nephropathy.^559^ The recently completed phase 2 trial found 24-week drug-dependent decreases in both 24-h urine albumin quantities and urinary albumin to creatinine ratios.^594^ Cilofexor was analyzed in phase 2 trials as an anti-NASH drug and was found to at 24 weeks reduce hepatic steatosis, improve liver biochemistry, and reduce serum BA concentrations.^553,594^ Two human trials have been conducted for HPG1860, a phase 1 pharmacokinetics study and a very recent phase 2a pharmacodynamic study in NASH patients.^597,598^ Although no results have been formally published, press releases from Hepagene indicate that phase 1 dosing saw no increases to LDL cholesterol and biomarker evidence of FXR-mediated suppression of BA synthesis. Press releases from the 12-week phase 2a trial discuss significantly decreased liver fat content, reduced serum alanine transaminase, a low incidence of pruritis, and no evidence of LDL cholesterol elevation.

TERN-101 has had two phase 2 trials initiated, LIFT for monotherapy against non-cirrhotic NASH, and DUET for TERN-101 and TERN-501 thyroid receptor beta agonist (TRβ) combination therapy. Within LIFT, TERN-101 monotherapy was non-superior at all doses compared to placebo for reducing alanine transaminase, although this may be an effect of the low study sample size.^599^ As of writing the DUET trial has no recorded results.^600^

A phase 1b trial for MET409 was performed in NASH patients in which it reduced hepatic adiposity, induced pruritis, and increased serum LDL cholesterol.^569^ A phase 2a trial was started for MET409 alone or in combination with empagliflozin for T2DM/NASH patients for which no results have been published.^601^ A phase 2 study on ileo-colonic delivery of UDCA had no major physiological effects, while two phase 3 trials are currently being recruited to test the effect of UDCA on statin-induced glucose intolerance and intestinal inflammation, respectively.^602–604^ Oral administration of 20 mg/kg/day of UDCA to morbidly obese patients was found to reproduce what is expected from preclinical studies, namely that gut-FXR inhibition increases cholesterol clearance as BAs and increases triglyceride synthesis.^605^

#### OCA—clinical outcomes and adverse effect profiles

The original OCA trials although somewhat effective were not without fault. In the phase 3 POISE trial evaluating 5–10 mg/day of OCA for PBC, OCA did reduce biomarkers of disease but significantly increased the risk of severe adverse events by ~3-fold in the 10 mg group compared to placebo.^606^ In addition, new-onset diabetes, dyslipidemia, and pruritis were dose-sensitive with OCA administration. Similar results were found against primary sclerosing cholangitis (PSC) with significant dose-dependent pruritis in the phase 2 AESOP trial, which included doses from 1–10 mg/day.^607^

Strong evidence of dose-dependent pharmacodynamics was identified within a phase 2 trial aimed at insulin resistance in NAFLD, in which OCA was tested at 25–50 mg/day within T2DM patients, most of which obese. The primary outcome of the trial was a two-step hyperinsulinemic-euglycemic clamp. Low-exposure insulin resistance was improved in the 25 mg/day group by 28.0%, while the high dose 50 mg/day group was non-significant with a point estimate of 20.1% improvement. High-exposure insulin resistance was improved by 18.3% in the 25 mg/day group, while the high dose 50 mg/day group was non-significant with a point estimate of 10.8% improvement.^608,609^ This lack of significance may be due to the small sample size of ~20 patients per group, but if interpreted as is, the trial reveals that OCA-mediated FXR agonism provides dose-dependent outcomes, where overshooting target agonist efficacy gives less favorable or potentially negative outcomes. Further illustrating such, increases to LDL cholesterol were found at all doses while decreased HDL cholesterol was only found at high doses.

Shifting focus to NASH indications, the phase 2 FLINT trial found that over 72 weeks 25 mg/day of OCA improved NASH histology. This was accompanied by increased VLDL cholesterol, LDL cholesterol, and decreased HDL cholesterol, replicating the FXR-associated preclinical adverse effect profile.^457,610^ Soon after FLINT, the phase 2 CONTROL trial found that 20 mg/day of atorvastatin was without surprise able to ameliorate FXR-induced LDL cholesterol induction by OCA dosed at 5–25 mg/day.^611^

#### What went wrong? OCA and the REGENERATE trial

Although OCA had somewhat positive results for PBC, PSC, NAFLD, and NASH, a common thread of adverse events persisted. The REGENERATE trial was the final nail in the coffin, whose original goal was to test the ability of OCA to treat pre-cirrhotic fibrosis secondary to NASH.^612^ Using results from the REGENERATE trial, the FDA rejected the NDA for the NASH indication of OCA, primarily due to its inability to at 10–25 mg/day meet the primary endpoint of improvement of NASH by more than one stage within 18 months. In addition, the FDA was cautious of the concerning benefit-to-risk profile of OCA due to its plentiful adverse effects, many of which were due to overtly strong systemic FXR agonism and repression of BA synthesis: drug-induced liver injury, excess risk of cholecystitis and bile stones, risk of new-onset dyslipidemia needing statin treatment, excessive risk for new-onset prediabetes/diabetes, acute kidney injury, and severe pruritis requiring discontinuation.^7^

In subsequent analyses of REGENERATE, OCA was able to obtain the primary endpoint but only by changing the method of pathological analysis from one pathologist to a consensus between three pathologists, in which OCA showed a dose-response relationship with the incidence of primary endpoint completion.

Let us now analyze one-by-one some of the most significant and near-significant adverse effects of the REGENERATE trial and try to mechanistically rationalize their occurrence. Dyslipidemia is explainable by our high total FXR model of lipid metabolism, high LDL cholesterol, low triglycerides, low VLDL cholesterol, and low HDL cholesterol. Short-term cardiovascular events may be explainable by the cardiomyotoxic or arrhythmogenic role of systemic FXR agonism, while longer-term results are explainable by a gut FXR-induced pro-atherogenic serum lipid profile. Gallbladder disease and related complications are directly explainable by FXR-mediated repression of hepatic BA synthesis, allowing for a more hydrophobic BA pool and cholesterol rich biliary fluid. A similar mechanism may explain drug-induced liver injury, in which a more hydrophobic and cytotoxic BA pool triggers hepatocyte damage. Hyperglycemia and new-onset diabetes is explainable by multiple factors: inability to allow for proper hepatic glycogen cycling due to overt GSK3β inhibition, reductions to systemic TGR5 agonism due to BA pool contraction, and reductions to hepatic and gut glycolysis, decreasing net GLP-1 secretion.

One last question remains, why do OCA and other FXR agonists cause pruritis? Human trials for OCA, tropifexor, cilofexor, EDP-305, and MET409 all identified pruritis.^555^ Therefore, pruritis is not induced by a steroidal scaffold or driven by ligand promiscuity at TGR5, but is instead FXR-intrinsic. Recently published research has found that FXR agonists such as OCA and cilofexor induce hepatic expression and secretion of the pruritogenic cytokine Interleukin-31 (IL-31), a large player in atopic dermatitis. Mechanistically, in PXB-chimeric mice it was found that serum IL-31 increased roughly 13-fold after OCA administration, supported by increasing hepatic and plasma IL-31 mRNA. Validation of this mechanism was found in humans, in which a dose-response relationship was found between IL-31 expression and cilofexor dosing in NASH patients. This dose-response relationship was not found for other competing causes, such as autotaxin-mediated lysophosphatidic acid synthesis. Pruritic severity in these patients was significantly correlated with serum IL-31 concentrations, establishing that FXR-induced IL-31 expression is the most plausible cause for OCA-induced pruritis.^613^

#### An old friend in disguise—is INT-787 aka TC-100 a FRESH take on FXR?

INT-787, a new name for TC-100 was recently announced by Intercept Therapeutics as their “next-generation” FXR agonist, primarily targeted at gut diseases and severe alcohol-associated hepatitis.^614^ Within an experiment in which HF diet ob/ob NASH model mice were given both INT-787 and OCA, only INT-787 lowered serum alanine transaminase, IL-1β, and TGFβ. Both OCA and INT-787 were able to restore the expression of matrix metalloprotease (MMP)-inhibitory Reversion-Inducing-Cysteine-Rich Protein with Kazul Motifs (RECK) proteins. OCA reduced MMP-2 and MMP-9 induction, while INT-787 only reduced MMP-2. Both OCA and INT-787 reduced A Disintegrin and Metalloproteinase Domain-Containing Protein 10 (ADAM10), 17 (ADAM17), and reduced the number of cytokeratin expressing hepatocytes. Lastly, INT-787 provided a stronger suppression of hepatic stellate cell activity compared to OCA.^615^

Although these initial results seem hopeful, more data is needed for a definitive conclusion. As previously discussed, the hydrophilic nature of TC-100 limits its hepatic exposure and provides a more gut-centric activity.^544^ Press briefings on the unpublished INT-787 phase 1 trial show evidence of outstandingly low non-dose-dependent pruritic risk and dose-dependent exposure, indicating that INT-787 may have a more favorable benefit-to-risk ratio compared to OCA.^616^ A human phase 2 trial on patients with severe alcohol-associated hepatitis named FRESH is currently recruiting with estimated trial results early to mid-2025.^617^

#### The next step forward for FXR—selective FXR modulators (SFXRMs)

IL-31 mediated pruritis, the induction of a pro-atherogenic serum lipid profile, and the potential risk of FGF19-[FGFR4/βK]-mediated carcinogenicity compel many to wonder if it is possible to selectively induce certain FXR-mediated downstream effects and prevent others? FXR transcriptional regulation of different genes is controlled by FXR isoform, tissue-specific coactivator/corepressor availability, and post-translational modification. From such, the next step forward in FXR modulator design should be towards the development of Selective FXR Modulators (SFXRMs). These modulators would conformationally support certain downstream effects, while weakly or absently supporting others, perhaps even producing tissue-dependent effects.^618^ We highlight three potential techniques to accomplish such. The first of which is to develop isoform-specific FXR modulators, biasing which FXRE-containing genes are expressed due to isoform-specific differences in FXRE motif preference.^98,619^ The second of which is to target orthosteric binding modes or allosteric sites that compete with or change the conformation of FXR domains responsible for tissue-dependent coactivator/corepressor recruitment.^620–622^ The last of which is to allosterically compete against FXR-modulator binding domains to prevent post-translational modification (acetylation/SUMOylation).^623^

Two molecules exhibit properties useful for future SFXRM development, one of which recruits a FXR corepressor while the other binds to an allosteric site and selectively modulates FXR activity.

The first of which is the marine sponge derived molecule theonellasterol (University of Perugia), which protects against cholestatic injury in mice.^624^ This FXR antagonist recruits Nuclear Receptor Corepressor 1 (NCOR1) to FXR and prevents its natural induction of OSTα, BSEP, and SHP. It also prevents FXR from repressing expression of MRP4, enhancing hepatic BA efflux in bile duct ligation mice models and preventing BA-overload induced toxicity.

The second of which is guggulsterone (Pharmacia Corp), which allosterically binds to FXR to prevent FXR association with coactivators, prevents unliganded-FXR release from corepressors, and prevents orthosteric ligand binding. In HepG2 cells, dose-titrations of guggulsterone enhances BSEP and represses SHP expression in the presence of CDCA, while in Caco2 cells, represses I-BABP expression in the presence of CDCA. In Huh-7 cells the effect on SHP was reversed, highlighting the tissue-dependence of coactivator recruitment. In rats, guggulsterone selectively induced BSEP and SHP expression without any FXR-mediated *CYP7A1*/*CYP8B1* repression, lowered triglycerides, and raised HDL cholesterol, potentially providing a solution for the FXR-plagued pro-atherogenic serum lipid profile.^625–627^ Although guggulsterone showed no effect on lipid profiles within a small human study, it serves as a crucial tool compound to motivate the future development of SFXRMs.^628^

### TGR5 modulators

#### Systemic TGR5 modulators

TGR5 agonists in recent years have gained popularity for their ability to stimulate GLP-1 secretion (Fig. 17). The most well-known potent and selective TGR5 agonist was originally discovered from structure-activity relationship studies on OCA, in which a single methyl group introduced to a suspected selectivity pocket provided strong TGR5 activity and removed most FXR activity.^8^ 23-(S)-Methyl-OCA, also known as INT-777 (Intercept Pharmaceuticals) was found to have micromolar potency and exhibit 166% of LCA’s documented effect of inducing cAMP synthesis.^629^ A further modified version of INT-777 is also pharmacologically active as a TGR5 agonist and is known as BAR501 (BAR Pharmaceuticals).^630^ Other semisynthetic TGR5 agonists have been discovered such as 7-Methoxy-CDCA (University of Minnesota), which along with recent discovery of TGR5 allostery inspired the design of the positive allosteric modulators 7-Methoxy-CA and 12-Oxo-7-Methoxy-CA, also known as compounds B1 and A1, respectively (Changzhou University).^631–633^ Alongside semisynthetic steroidal scaffolds, natural steroidal TGR5 agonists have been discovered from plant extracts with hypoglycemic effects. These plant extracts were refined to discover the triterpenoids betulinic acid, oleanolic acid, and ursolic acid, all of which exhibit potent and selective TGR5 agonism (Louis Pasteur University).^634,635^

The first major non-steroidal TGR5 agonists were the 3-aryl-4-isoxazolecarboxamide scaffolds (GlaxoSmithKline), which exhibited decent pharmacological effects if given via an intrajejunal infusion to dogs but suffered from very poor oral bioavailability.^636,637^ Following from such the 1,4-bis(sulfonyl)-1,4-diazepane scaffold SB-756050 was discovered, which touted micromolar potency and full specificity for TGR5 (GlaxoSmithKline). When orally administered to humans in a phase 1 trial, SB-756050 exhibited horrible human non-linear pharmacokinetics and highly patient-dependent pharmacodynamic effects.^638^ Besides this trial, no notable in human TGR5 modulator trials have been reported. In response to this, TGR5 agonist development branched into diverse chemical space in an attempt to produce an orally bioavailable scaffold. Non-steroidal scaffolds such as imidazo[1,2-a]pyrimidine (Torrent Pharmaceuticals), 2-aryl-3-aminomethylquinoline (Kalypsys), triazole (Pfizer), tetrahydropyrido[4,3-d]pyrimidine (Pfizer), (pyrimidin-2-yl)azetidine (Novartis), nicotinamide (Shanghai institute of Materia Medica), isonicotinamide (Novartis), 3-phenoxypyrazine-2-carboxamide (Henan University), dihydropyridone (Pasteur institute), benzothiazole (Eli Lilly), pyridine (Roche/Takeda), and 2-thio-imidazole (Zydus Pharmaceuticals) have been reported.^289,639–648^

#### Preclinical results—systemic TGR5 modulators

In C57BL/6J mice INT-777 has been found to strongly induce GLP-1 secretion, enhance insulin-induced glucose uptake into skeletal muscle, protect against HF DIO mice via DIO2/UCP1 induction, protect against steatosis by repressing hepatic triglyceride synthesis, and inhibit gluconeogenesis by inhibition of FOXO1 and repression of G6Pase/PEPCK.^380,649^ The positive allosteric modulators B1 and A1 have been found to increase the intrinsic efficacy of CDCA agonism at TGR5, but not increase total maximal cAMP induction.^632^

Multiple non-steroidal scaffolds have been able to either induce GLP-1 secretion and/or reduce oral glucose tolerance test (OGTT) Area Under the Curve (AUC) in either C57BL/6J mice or human NCI-H716 cells.^639–643,645–647^ In HF high fructose fed mice, the orally administered steroidal TGR5 agonist BAR501 was able to reverse insulin resistance, reverse histological markers of NASH, and increase BADT energy expenditure by inducing the expression of UCP1/PGC-1α.^650^ The imidazo[1,2-a]pyrimidine scaffold TRC210258 was found to lower triglyceride levels, LDL cholesterol levels, increase HDL cholesterol, and improve glycemic control within DIO mice and hamsters.^644^ Viewing cardiometabolic health, INT-777 administration inhibited ASCVD development in HC fed *LDLr*^*−/−*^ mice. This was mechanistically explained to be a downstream effect of elevated cAMP levels, which repressed NF-κB activation.^279^ This result was replicated when dual knockout *ApoE*^*−/−*^ and *LDLr*^*−/−*^ mice were treated with the dual FXR/TGR5 agonists, but was lost once the model was expanded to a *ApoE*^*−/−*^ *LDLr*^*−/−*^ *TGR5*^*−/−*^ triple knockout.^651,652^

The isonicotinamide TGR5 agonist scaffold was able to successfully inhibit TNFα and IL-12 synthesis both in human monocytes and mice models, additionally stabilizing macrophages within the M2 anti-inflammatory phenotype. This effect was reversed in *TGR5*^*−/−*^ mice.^289^ Similarly, BAR501 administration was shown to similarly reinforce M2 macrophage phenotypes during mice models of colitis, along with suppressed expression of TNFα, IL-1β, IL-7, and IFNγ.^284^ Both examples highlight how TGR5 agonists may serve as immunomodulators in chronic inflammation.

#### What’s wrong with systemic TGR5 modulators?

Analogous to the adverse effect profile of systemic FXR agonists, systemic TGR5 agonists have their burden of adverse effects. Alongside their general positive physiological role, preclinical doses high enough to induce GLP-1 expression have also been found to negatively impact gallbladder function, producing a gallbladder that is overfilled and unresponsive to CCK-induced contraction. Mechanistically, cAMP-induced CFTR agonism enhances biliary flow to fill the gallbladder, while cAMP-activated PKA phosphorylates myosin light chain kinase, inhibiting its ability to produce smooth muscle contraction.^269,653^ The gallbladder effects of systemic TGR5 agonists are very similar to pruritis for systemic FXR agonists, in that both are intrinsically caused by receptor activation and are not due to ligand ambiguity.^647,648,653^

#### Leveraging size, polarity, and metabolic stability to save the gallbladder

To remove TGR5-mediated side effects at the gallbladder many have attempted to produce gut-restricted TGR5 agonists, providing GLP-1 release without significant exposure to the gallbladder. Three major strategies have been implemented to obtain gut-restricted activity: increasing molecular weight, introducing a very polar motif known as a kinetophore to reduce membrane permeability, or to introduce a metabolic soft spot.^654^ Each of these approaches has its own intrinsic challenge. TGR5 is expressed basolaterally within enterocytes, meaning that gut-restricted agonists must retain low systemic permeability but enough permeability to reach the basolateral enterocyte surface in meaningful quantities. Endogenous BAs are able to bypass this requirement and reach the basolateral surface in small quantities via passive diffusion or substantial quantities by using ASBT/I-BABP/(OSTα/β-MRP3)-mediated transport.^655,656^

#### Preclinical results—gut-restricted TGR5 modulators

The first attempts at a gut-restricted TGR5 agonist employed the usage of a large molecular weight compound. Compound 15c (Shanghai Institute of Materia Medica) was designed as a large ~1400 Da, ~230 Å^2^ polar surface area, low permeability, and pseudosymmetric molecule, based upon the previously discovered nicotinamide scaffold (Compound 2.1 - Shanghai Institute of Materia Medica).^647^ Non-trivially this large molecule appears systemically with nanomolar concentrations within the bile and serum. The Caco-2 efflux ratio of 61 indicates that in normal enterocytes reverse efflux may limit basolateral exposure, while within enteroendocrine L-cells it may successfully accumulate on the basolateral face.^657^ This molecule exhibited enhanced GLP-1 secretion in human and mice in vitro models, along with a significantly reduced effect on gallbladder filling relative to compound 2.1 within mice models.^658^

Using the kinetophore approach the same group built upon this design further by including quaternary ammonium groups to mimic that of the BA sequestrant cholestyramine to generate compound 26a (Shanghai Institute of Materia Medica). Compound 26a was found to have even lower intestinal permeability compared to 15c and was able to produce long-lasting hypoglycemia in ob/ob mice by inducing GLP-1 secretion, however, had complicated long-term effects on gallbladder filling.^659^ Compound 15c was modified by thiophene replacement to a thiazolidine and the replacement of the quaternary ammonium groups to a polyol-bearing motif to produce compound 12 (Ardelyx Therapeutics). Compound 12 had more predictable gallbladder effects and sustained mice GLP-1 secretion.^660^ Other approaches have been used within this same kinetophore approach, such as the addition of sulfonate motifs (Compound 24 - Pasteur Institute/Compound 22-Na - Shanghai Institute of Materia Medica), carboxylic acid motifs (Compound 19.1 - Shanghai Institute of Materia Medica), polyol motifs (Compound 30-Na - Shanghai Institute of Materia Medica), and PEGylated motifs (PEG-Compound 24 - Pasteur Institute), many of which exhibiting drastically reduced TGR5-mediated effects on gallbladder filling.^654,657,661,662^

Compound 19.1 was further modified by the implementation of an ethoxymethyl ester motif to produce compound 19.2 (Shanghai Institute of Materia Medica). The design of compound 19.2 improved from that of compound 19.1 and all those before it by the incorporation of a plasma metabolic soft spot. If the compound was able to partition into the systemic circulation, it would be hydrolyzed by plasma esterases and become inactive as a TGR5 agonist. This allowed for compound 19.2 to exert strong TGR5 agonism and exhibit good gut-permeability without worrying about systemic exposure. As a result, compound 19.2 exhibited multiple-fold increases in GLP-1 secretion in human and mice in vitro models, reduced AUC for oral glucose tolerance tests in mice, had near-zero detection in the plasma or gallbladder in mice, and displayed non-significant increases to gallbladder weight.^663^

#### Can gut-restricted TGR5 agonists become the next Ozempic?

Current pharmacological techniques to manipulate GLP-1 receptor agonism are based on peptidic injectable formulations or peptidic oral formulations co-formulated with salcaprozate sodium to protect against gastric acid-catalyzed hydrolysis.^664^ The discovery and synthesis of gut-restricted TGR5 agonists with minimal systemic exposure provides an exciting, oral, non-peptidic, and paradigm-shifting opportunity for future GLP-1 relevant drug discovery. Although this new modality for treatment seems promising, no human trials have been conducted yet.

### Novel BA-centric targets against disease

#### Preclinical FXR/TGR5 dual agonists treat metabolic disease and the TNFα-[NF-κB] axis

Alongside mutually exclusive FXR and TGR5 modulators, developments have been made to discover novel BA-centric therapeutic approaches (Table 2). INT-767 (Intercept Pharmaceuticals) is a dual FXR/TGR5 agonist that displays properties consistent with the pharmacology of both FXR and TGR5. Mimicking a pure FXR agonist, INT-767 represses *CYP7A1*/*CYP8B1* transcription within the classical pathway of BA synthesis and by unknown mechanisms induces the expression of alternative pathway genes. Similar to that of a pure TGR5 agonist, INT-767 has been found to induce cAMP levels, raise intracellular calcium levels, and enhance GLP-1 secretion, improving insulin resistance, lipid tolerance, and mitochondrial function within DIO mice.^208,665^ Additional studies have found that INT-767 has a unique role in reversing HF DIO by normalizing glucose metabolism, NAFLD by repressing both lipogenesis and inflammation, and NASH by reducing histological disease severity and TNFα-induced NF-κB activity in mice.^290,666,667^

**Table 2:** BA-centric drug discovery: pharmacological classes, impacted diseases, rationale, and example compounds

Pharmacological class:	Proven & predicted target diseases:	Mechanistic rationale:	Example compounds:
FXR Agonist (Steroidal)	Proven: T2DM, Obesity, NAFLD/NASH, IBD	FXR-induced repression of gluconeogenesis, lipogenesis, and fibrosis. Gut FXR-FGF19 repression of hunger.	CDCA, OCA aka INT-747, EDP-305, INT-787 aka TC-100, MFA-1
FXR Agonist (Non-Steroidal)	Proven: T2DM, Obesity, NAFLD/NASH, IBD	FXR-induced repression of gluconeogenesis, lipogenesis, and fibrosis. Gut FXR-FGF19 repression of hunger.	GW4064, Tropifexor, Cilofexor, WAY-362450, Nidufexor, MET409, TERN-101, BMS-986318, BMS-986339, HPG1860, LH-10, Licraside, Compound 27, INT-2228
FXR Agonist (Gut Restricted)	Proven: T2DM, Obesity, NAFLD/NASH, IBD	Gut FXR-FGF19 repression of hunger, change in BA pool towards TGR5 agonism, and TGR5-independent TGR5 pharmacology.	Fexaramine
FXR Antagonist	Proven: T2DM, Obesity, NAFLD/NASH, ASCVD, IBD	Inhibition of gut FXR ceramide synthesis.	UDCA, MCAs
FXR Antagonist (Gut Restricted)	Proven: T2DM, Obesity, NAFLD/NASH, ASCVD	Inhibition of gut FXR ceramide synthesis without inhibiting hepatic FXR.	Compound F6, V023-9340
SFXRMs	Predicted: All Systemic/Gut FXR Agonist Indications	Providing mechanistic selectivity to minimize common FXR agonist adverse effects.	Theonellasterol and Guggulsterone
TRG5 Agonist (Steroidal)	Proven: T2DM, Obesity, NAFLD/NASH Predicted: ASCVD	Increased ceramide metabolism, GLP-1 secretion, basal metabolic rate, ADIPOQ secretion, and NF-κB inhibition.	INT-777, BAR501, 7-Methoxy-CDCA, 7-Methoxy-CA, Compound B1, Compound A1, Betulinic Acid, Oleanolic Acid, Ursolic Acid
TGR5 Agonist (Non-Steroidal)	Proven: T2DM, Obesity, NAFLD/NASH, ASCVD	Increased ceramide metabolism, GLP-1 secretion, basal metabolic rate, ADIPOQ secretion, and NF-κB inhibition.	Compound 2.1, SB-756050, and the following structural classes: - 3-aryl-4-isoxazolecarboxamide - Imidazo[1,2-a]pyrimidine - 2-aryl-3-aminomethylquinoline - Triazole - Tetrahydropyrido[4,3-d]pyrimidine - (pyrimidin-2-yl)azetidine - Nicotinamide - Isonicotinamide - 3-phenoxypyrazine-2-carboxamide - Dihydropyridone - Benzothiazole - Pyridine - 2-thio-imidazole
TGR5 Agonist (Gut Restricted)	Proven: T2DM Predicted: Obesity, NAFLD/NASH, ASCVD	Increased GLP-1 secretion.	Compounds 15c, 26a, 12, 14, 22 Na, 19.2, 30 Na, 24, 19.1, 19.2
Novel Target – FXR/TGR5 Dual Agonist	Predicted: T2DM, Obesity, NAFLD/NASH	Combination of (Steroidal/Non-Steroidal) FXR/TGR5 Agonists	INT-767 and Deoxyschizandrin
Novel Target – FXR Agonist & URAT1 Antagonist	Predicted: Gout	FXR-mediated anti-inflammatory effects and uricosuric effects of URAT1 inhibition.	Compound 4
Novel Target – Gut Restricted FXR Agonist & FABP1 Antagonist	Predicted: Obesity, NAFLD, ASCVD	Gut FXR agonist effects and reduced fatty acid absorption via FABP1 antagonism.	ZLY28
Novel Target – TGR5 Agonist & CysLT_1_R Antagonist	Predicted: NASH	TGR5 agonist effects and reduced hepatic inflammation via CysLT_1_R.	Compound 2.2
Novel Target – TGR5 Agonist & RORγt Inverse Agonist	Predicted: IBD	TGR5 agonist effects and reduced Th17 differentiation via RORγt inverse agonism.	Compound 7
Novel Target – FXR/LXR Dual Agonist	Predicted: NAFLD/NASH	FXR agonist effects and LXR agonism-mediated changes to lipid synthesis. (Warning: Species-specific effects of LXR agonism)	Withaferin A
Novel Target – FXR Antagonist & PXR Agonist	Predicted: PSC, PBC, IBD.	FXR antagonism enhances biliary flow, while PXR repressed inflammatory cytokine expression.	Compound 5 and Compound 11
Novel Target – FXR/PPARγ Dual Partial Agonist	Predicted: T2DM, Obesity, NAFLD/NASH	FXR agonist effects and insulin sensitization via PPARγ agonism.	Compound 18
Novel Target – YY1 Antagonist	Predicted: NAFLD/NASH	Enhanced hepatic FXR expression, reducing steatosis and inhibiting fibrosis.	miRNA: 7, 29a, 181, 186, 218 NO Donors: DETA NONOate
Novel Target – FGF19 Analog (FGFR4/βK Agonist)	Proven: T2DM, Obesity, NAFLD/NASH	Inhibition of hypothalamic hunger center and repression of SREBP-1C target genes.	Aldafermin aka NGM282
Novel Target – CYP8B1 Antagonist	Predicted: T2DM, Obesity, NAFLD/NASH	Increased GLP-1 secretion.	None to Date
Novel Target – FGFR4 Antagonist	Proven: Cancers with phenotypes of FGFR4-overexpression (Ex: Hepatocellular Carcinoma)	Reversal of FGFR4-overexpression chemotherapeutic resistance.	ASP5878, PRN-1371, JNJ-42756493 aka Erdafitinib, NVP-BGJ398, H3B-6527, FGF401, INCB0620709, BLU-554
Novel Target – BA Sequestrant	Proven: T2DM, ASCVD Predicted: Obesity, NAFLD/NASH	Increased BA exposure to enteroendocrine L-cells enhancing GLP-1 secretion, and excreting LDL cholesterol as BAs.	Colesevelam, Cholestyramine, Colestipol
Novel Target – ASBT Antagonist	Proven: NASH Predicted: T2DM, Obesity, NAFLD, ASCVD	Lowers BA entry into enterocytes to agonize gut FXR, enhances BA exposure to enteroendocrine L-cells for higher GLP-1 release.	Odevixibat, SC-435, Maralixibat, Vorilixibat
Novel Target – Gut Microbiome Modifiers	Proven: T2DM/NASH Predicted: Obesity, NAFLD, IBD, ASCVD	Increasing *BSH*-containing bacteria increases UDCA BA pool composition, reducing gut FXR-induced ceramide synthesis, additionally reducing LDL cholesterol by shunting it into BA synthesis. Reducing *BSH*-containing bacteria increases BA pool composition of TGR5 agonists.	VSL#3 Probiotic and Metformin

Besides INT-767, similar results have been found when analyzing the phytochemical FXR/TGR5 dual agonist deoxyschizandrin (Longhua Hospital). Deoxyschizandrin was found to counter HF DIO in mice by promoting energy expenditure, anorexia, and leptin sensitivity. Within both HF DIO and methionine and choline-deficient L-amino acid diet mice, deoxyschizandrin was found to effectively reduce NAFLD hepatic phenotypes.^668^

#### Interplay between FXR/TGR5 dual agonists and the TNFα-[NF-κB]-YY1-*FXR* axis

The TNFα-[NF-κB] axis directly induces the expression of the *FXR* repressor YY1, another unique target for NAFLD/NASH drug discovery.^669^ If one wishes to enhance the expression and activity of FXR in NAFLD or NASH, it is then natural to wish to inhibit the activity of or repress the expression of YY1. FXR/TGR5 dual agonists such as INT-767 directly inhibit the TNFα-[NF-κB] axis, analogous to known techniques already employed to inhibit the expression of YY1.^670^ Besides upstream regulation, YY1 targeted therapy is likely to provide stronger and more relevant impacts for therapeutic modulation of NAFLD/NASH. Many approaches have already been attempted to inhibit YY1. Besides upstream NF-κB inhibition, others have used YY1 targeted siRNA or the administration of nitric oxide (NO) donors such as Diethylenetriamine (DETA) NONOate, which S-nitrosylate and inhibit the YY1 DNA binding domain.^670–677^ No small molecule competitive antagonist for YY1 has been discovered, opening a wealth of opportunity in drug discovery, perhaps being a prime target for PROTAC development.

#### Preclinical FXR/URAT1, FXR/FABP1, TGR5/CysLT_1_R, and TGR5/RORγt modulators

In contrast to INT-767 and deoxyschizandrin, other dual receptor modulators have been found that impact both a BA-centric receptor and another non-BA-centric disease specific receptor. The first of which was compound 4 (Guangdong University), which was found to function as a FXR agonist and a Human Urate Transporter 1 (URAT1) inhibitor. Mechanistically it is thought that FXR-mediated inhibition of the NLRP3 inflammasome will decrease gout-associated inflammation, while URAT1 inhibition will function as a uricosuric, enhancing uric acid excretion in the urine.^678^

The dual modulator ZLY28 (Guangdong Pharmaceutical University) was discovered to both be a gut-restricted FXR agonist and Fatty Acid Binding Protein 1 (FABP1) antagonist. FABP1 is essential for lipid chaperoning and the gut-mediated absorption of fatty acids. FABP1 antagonism and FXR agonism therefore is synergistic to treat NASH. Within CCl_4_ treated mice this compound exhibited full FXR agonism within the gut evidenced by FGF19-mediated effects hepatically, but no systemic FXR agonism. Alongside this, reduced hepatic steatosis, inflammation, and ballooning was observed with significantly lower hepatic triglyceride accumulation compared to OCA, indicating synergistic efficacy.^679^

The dual modulator compound 2.2 (University of Perugia) was found to be a TGR5 agonist and a Cysteinyl Leukotriene Receptor 1 (CysLT_1_R) antagonist. Compound 2.2 was designed as an anti-NASH drug, which was found to inhibit hepatic steatosis, the hepatic expression of inflammatory biomarkers, and prevent DIO in HF HC mice.^680^

The novel RORγt inverse agonist and TGR5 agonist compound 7 (University of Naples) has been shown to provide robust anti-inflammatory activity in mice models of TNBS-induced colitis, reducing TNFα, IL-1β, and IL-6 expression while increasing IL-10 and TGFβ expression. This compound provides immense potential as a therapeutic against IBD by inhibiting both NF-κB/NLRP3 signaling and the differentiation of Th17 cells.^681^

#### Preclinical FXR/LXR, FXR/PXR, and FXR/PPARγ modulators

Two dual modulators have been discovered that impact both BA-canonical and non-canonical receptors. The first of which is the phytochemical withaferin A (JSS Medical College), which was found to be a LXR/FXR dual agonist. Withaferin A was found to potently suppress steatosis, fibrosis, and TGFβ expression in HF diet NAFLD mice, in addition to preventing lipid droplet accumulation in HepG2 and Huh7 cells.^682^

The second canonical/non-canonical dual modulators are compounds 5/11 (University of Naples), found to be FXR antagonists and PXR agonists. These compounds were able to repress FXR-mediated activity, suppress IL-8, and suppress IL-1β expression in HepG2 cells. Repressed FXR agonism may enhance biliary flow and help cholestatic diseases, while PXR-induced IL-8/IL-1β suppression may provide benefit in IBDs.^683,684^

The last canonical/non-canonical dual modulator discussed is compound 18 (Hiroshima International University), a novel dual partial agonist of FXR and PPARγ providing strong mechanistic synergy for T2DM-induced NAFLD. Within Huh7 cells compound 18 was found to slightly increase BSEP/OSTα expression relative to an inactive control, while markedly inducing SHP expression and repressing SREBP-1C expression. Phosphorylation of PPARγ at S273 has been associated with higher levels of insulin resistance. Administration of compound 18 to 3T3-L1 cells was found to suppress PPARγ S273 phosphorylation as potent as the FDA-approved PPARγ agonist rosiglitazone.^685^ Cumulatively PPARγ-mediated insulin sensitization in conjunction with FXR-mediated hepatic effects may produce a strong clinical candidate. Although it remains to be seen if the PPARγ agonism produces the same side effects that make currently available PPARγ agonists undesirable, such as weight gain, increased fluid retention, and a higher risk for the development of congestive heart failure.^686^

#### Biasing BA pool composition, can we inhibit CYP8B1?

Due to the association of 12α-OH BAs in T2DM, obesity, and NAFLD/NASH, immense interest has been generated in the drug discovery of CYP8B1 inhibitors. In perfect timing the enzymatic characterization and X-ray crystal structure of CYP8B1 has been published within the last year, allowing for structure-based drug design.^687^ CYP8B1 inhibition may serve as an excellent target for pharmacological inhibition, since shunting BA synthesis into the alternative pathway will produce a similar effect to that of fexaramine, producing a more TGR5 agonistic BA pool.

#### Preclinical and clinical benefits of FGF19 analogs

In the same nature of modifying endogenous BA physiology to impact disease, recent efforts have focused on the synthesis and characterization of FGF15/19 analogs. Subcutaneous FGF19 administration was found to be anti-diabetic and anti-obesogenic by inhibiting AgRP/NPY neurons within the hypothalamic hunger center of mice.^126,127^ This results in an overall reduction in caloric intake leading to reduced weight gain and improved insulin sensitivity. The subcutaneous administration of the FGF19 analog Aldafermin, also known as NGM282 (NGMBio), has been shown to provide a rapid, sustained, and robust decrease in hepatic lipid content along with improvements to NASH histopathology in both animal models and human clinical trials.^688–690^

#### Potential solutions against FGFR4-mediated carcinogenic impacts

Although the FGF15/19-[FGFR4/βK] axis provides benefits like those provided by GLP-1 agonists, overactive FGF15/19-[FGFR4/βK] axis activity as previously discussed can be carcinogenic. From such, many pharmaceutical companies have developed treatments that may target cancer cells that use FGFR4/βK overexpression as a mechanism for chemotherapeutic resistance, developing pan-FGFR inhibitors and selective FGFR4 inhibitors.^691,692^ Similar to other cases of chemotherapeutic resistance, mutations have been found in FGFR4 such as FGFR4-V550L or FGFR4-V550M which limit the design and applicability of current FGFR4 antagonists. In line with parallel research on FGFR2, PROTAC-mediated FGFR4 degradation may allow for one to target regions of the receptor not intrinsically associated with inhibition, providing more opportunities against mutation.^693–695^

#### BA sequestrants increase TGR5 agonism and GLP-1 release

Besides centrally acting targets, effort has been put into finding gut-restricted non-BA-receptor targets. The first of which is a repurposing of the already FDA-approved BA sequestrant anionic exchange resins: colesevelam, cholestyramine, and colestipol. BA sequestrants prevent the enteric reabsorption of BAs, increasing the luminal concentration of BAs present in the distal gastrointestinal tract. BA sequestrants increase the fecal excretion of BAs, lowering systemic LDL cholesterol by shunting it into BA synthesis. This leads to increased BA exposure and agonism of enteroendocrine L-cell TGR5, enhancing systemic GLP-1 secretion in mice.^696^ The enhanced GLP-1 secretion was correlated with improved insulin sensitivity and suppressed hepatic glycogenolysis in a DIO rat model.^697,698^ Alongside DIO rat models, this GLP-1 secretory outcome was found and validated in a systemic review of phase II human trials, serving as evidence for the potential usage of BA sequestrants not just for hypercholesterolemia but as an insulin-sensitizing and anorexic therapeutic.^699–701^

#### ASBT inhibitors increase BA synthesis and reduce cholestatic risk

Analogous to the role of BA sequestrants, ASBT inhibitors prevent the ileal reuptake of BAs. Decreased ileal reuptake of BAs reduces hepatic FXR agonism and enhances hepatic BA synthesis and flux through the biliary tree, lowering serum cholesterol and cholestatic risk.^702^ Preliminary results in *MRP2*^*−/−*^ mice models have indicated that ASBT inhibitors have a functional role in the treatment of PSC, inhibiting inflammatory/profibrotic gene expression.^703^ The ASBT inhibitor A4250 also known as Odevixibat (Alibero Pharma) was already FDA-approved as a first-in-class drug used to treat familial intrahepatic cholestasis, while the second compound SC-435 (University of Connecticut) has been used purely as a test compound.^704–706^ Alongside these current compounds, two new ASBT inhibitors are in human clinical trials, volixibat (Mirium Pharmaceuticals) and maralixibat (Mirium Pharmaceuticals). Maralixibat is currently approved for Alagille syndrome and recently completed the phase 2b/3 MARCH trial for progressive familial intrahepatic cholestasis, showing positive safety and efficacy data.^707^ Volixibat is currently in in two phase 2b/3b trials, VISTAS for PSC, and VANTAGE for PBC.^708,709^ Both of which are likely to be approved by 2027-2028.

#### Microbial modification changes BA pool composition

The last gut-restricted non-BA-receptor target is that of the gut microbiome. Modification of the gut microbiome directly impacts both the primary to secondary and conjugated to unconjugated ratios within the BA pool.^710,711^ Current efforts are to either pharmacologically reduce *BSH*-containing bacterial or administer probiotic/prebiotic mixes to enrich for *BSH*-containing bacteria.^712^ An example of the first technique is exemplified with metformin, which by unknown mechanisms decreases *Bacteroides fragilis*, which in mice increased gut UDCA levels and provided the positive benefit of reducing ceramide synthesis.^713^ The alternative approach is highlighted by the probiotic supplement VSL#3 (VSL3 Pharma), which has been shown to induce BA deconjugation and maturation into secondary BAs, induce BA synthesis, enhance insulin sensitivity by driving BA pool composition to favor TGR5 agonism, and protect mice from NASH.^714,715^ Alongside BA pool composition changes it is theorized that a higher prevalence of *BSH*-containing bacteria reduces the surfactant efficacy of gut BAs, reducing systemic LDL cholesterol by inhibiting cholesterol absorption and increasing cholesterol excretion as BAs.^54^

## Conclusions and future perspectives

### Continuing with BAs—recommendations for future success

It is essential to acknowledge the pharmacological complexity of BA receptor modulators, exemplified by OCA’s capacity to impact numerous physiological circuits and induce severe side effects. Messy pharmacology, low bioavailability, dose-dependent cytotoxicity, and non-linear pharmacokinetics have plagued BA-centric drug discovery. However, we believe that dismissing BA research prematurely is unwarranted, since significant progress has been made addressing these concerns and unlocking new potential for BA receptor modulators.

Messy receptor pharmacology has been substantially improved by developing tissue-specific drugs. Many of the original side effects of OCA have been directly remedied, or at least mechanistically understood to pave the way for future innovation. For instance, hepatic FXR-mediated IL-31 pruritis has been strongly mitigated by the usage of gut-restricted FXR agonists. Although challenges such as a pro-atherogenic serum lipid profile persist, emerging strategies such as SFXRMs, the discovery and usage of new non-steroidal scaffolds, and scaffold-specific structure activity relationships have been found to meaningfully reduce adverse effect incidence and severity. Similarly, positive developments have been seen in the case of TGR5 agonists, in which the initial alarming concern about gallbladder non-contractility has been solved by the implementation of gut-restricted TGR5 agonists. Despite the intrinsic complications of modulating FXR and TGR5, tremendous preclinical experimentation and clinical validation has given us the tools to remove less favorable outcomes, maximizing the chance of therapeutic success (Table 3).

**Table 3:** Approved or candidate BA-centric drugs and their clinical trials

Pharmacological class	Name	Clinical trial(s)	Trial indications	Approval phase
Steroidal FXR Agonist	OCA	- *NCT01473524* - *NCT02177136* - *NCT00501592* - *NCT01265498* - *NCT02633956* - *NCT02548351* - *NTC1625026* - *NCT02308111*	- PBC - NAFLD/NASH - Gallstones/Obesity - PSC	- PBC (Approved) - NAFLD (Phase 2) - NASH (Phase 2) - Gallstones/Obesity (Phase 2) - PSC (Phase 2)
	EDP-305	- *NCT03421431* - *NCT03394924*	- PBC - NASH	- PBC (Phase 2) - NASH (Phase 2)
	INT-787 aka TC-100	- *NCT05639543*	- Severe Alcohol-Associated Hepatitis	- Phase 2
Steroidal FXR Antagonist	UDCA	- *NCT02871882* - *NCT02033876* - *NCT03724175* - *NCT05500937*	- T2DM - Intestinal Inflammation	- T2DM (Phase 2) - Intestinal Inflammation (Phase 2/3)
Non-Steroidal FXR Agonist	PX-102	- *NCT01998659* - *NCT01998672*	- Healthy Patient Single/Multiple Ascending Dose	- Phase 1
	Tropifexor	- *NCT02855164* - *NCT02516605*	- NASH - PBC	- NASH (Phase 2) - PBC (Phase)
	Cilofexor	- *NCT02854605* - *NCT02781584*	- NASH	- Phase 2
	WAY-362450 aka FXR-450	- *NCT00509756* - *NCT00509756*	- Safety/Tolerability in Healthy Patients	- Phase 1
	HPG1860	- *NCT04480697* - *NCT05338034*	- Safety/Tolerability in Healthy Patients - NASH	- Phase 2a
Non-Steroidal FXR Partial Agonists	Nidufexor	- *NCT03804879*	- T2DM/Nephropathy	- Phase 2
	MET409	- *NCT04702490*	- T2DM/NASH	- Phase 2
	TERN-101	- *NCT04328077* - *NCT05415722*	- Non-Cirrhotic NASH	- Phase 2
Non-Steroidal TGR5 Agonist	SB-756050	- *NCT00733577*	- T2DM	- Phase 1
FGF19 Analog	Aldafermin aka NGM282	- *NCT02443116* - *NCT01943045*	- T2DM - NASH	- Both (Phase 2)
BA Sequestrants	Colesevelam	- *NCT01066364* - *NCT00484419* - *NCT00690937* - *NCT00147745* - *NCT00789750* - *NCT00151762* - *NCT00151749* - *NCT00147719* - *NCT00147758* - *NCT00789737* - *NCT00361153* - *NCT00570739* - *NCT01066364* - *NCT00990184* - *NCT00993824* - *NCT00951899* - *NCT00951899* - *NCT00151749* - *NCT00361153*	- ASCVD & Hypercholesterolemia - T2DM	- ASCVD & Hypercholesterolemia (Approved) - T2DM (Approved)
	Cholestyramine	- *NCT00000594* - *NCT00000463* - *NCT00000488* - *NCT00000461* - *NCT03510715* - *NCT03510884*	- ASCVD & Hypercholesterolemia	- ASCVD & Hypercholesterolemia (Approved)
	Colestipol	- *NCT00203476* - *NCT00000512* - *NCT00000599*	- ASCVD & Hypercholesterolemia	- ASCVD & Hypercholesterolemia (Approved)
ASBT Inhibitor	Odevixibat	- *NCT04483531* - *NCT03566238* - *NCT03659916* - *NCT04674761* - *NCT05035030* - *NCT04336722*	- Progressive Familial Intrahepatic Cholestasis - Alagille Syndrome - Biliary Atresia	- Alagille Syndrome/Biliary Atresia (Phase 3) - Progressive Familial Intrahepatic Cholestasis (Approved)
	Maralixibat	- *NCT03905330* - *NCT02160782*	- Progressive Familial Intrahepatic Cholestasis - Alagille Syndrome	- Progressive Familial Intrahepatic Cholestasis (Phase 3) - Alagille Syndrome (Approved)
	Vorilixibat	- *NCT05050136* - *NCT04663308*	- PSC - PBC	- Phase 2b-3
Pan-EGFR Inhibitors	ASP5878	- *NCT02038673*	- Solid Tumors	- Phase 1
	PRN-1371	- *NCT02608125*	- Solid Tumors	- Phase 1
	JNJ-42756493 aka Erdafitinib	- *NCT02421185* - *NCT02365597* - *NCT03238196* - *NCT04172675*	- Hepatocellular Carcinoma - Urothelial Cancer - Metastatic Breast Cancer - Urinary Bladder Neoplasms	- Urothelial Cancer (Phase 3) - Others (Phase 2)
	NVP-BGJ398	- *NCT01975701* - *NCT03510455* - *NCT03773302* - *NCT04197986*	- Recurrent Resectable Unresectable Glioblastoma - Cholangiocarcinoma - Urothelial Carcinoma	- Glioblastoma (Phase 2) - Cholangiocarcinoma (Phase 3) - Urothelial Carcinoma (Phase 3)
Selective FGFR4 Inhibitors	H3B-6527	- *NCT03424577*	- Hepatocellular Carcinoma	- Phase 1
	FGF401	- *NCT02325739*	- Hepatocellular Carcinoma	- Phase 1-2
	INCB0620709	- *NCT03144661*	- Hepatocellular Carcinoma - Esophageal Cancer - Nasopharyngeal Cancer - Ovarian Cancer - Solid Tumors	- Phase 1
	BLU-554	- *NCT02508467* - *NCT04194801*	- Hepatocellular Carcinoma	Phase 1/2

Improving low oral bioavailability, dose-dependent cytotoxicity, and non-linear pharmacokinetics have been the strongest forces behind non-steroidal scaffold research. Besides the negative outcomes of WAY-362450 for FXR and SB-756050 for TGR5, most if not all non-steroidal scaffolds have shown dramatically improved pharmacokinetic parameters. Diverging from endogenous BAs, non-steroidal or heavily modified steroidal scaffolds provide a refined approach for clinical development, removing most of the amphipathic surfactant-like effects that trigger dose-dependent cytotoxicity.

Recent advancements in both tissue-targeted and structurally diverse approaches have provided ample opportunities to wrangle the originally untamable beast of BA physiology. Combining this with the incredibly important role of BA physiology in the pathogenesis of cardiometabolic, inflammatory, and neoplastic diseases, there is immense clinical potential and a high likelihood for the future success of BA-centric therapeutics.

### How may BA therapeutics be better or worse than the existing therapeutic paradigm?

The current landscape of T2DM treatment predominantly revolves around generating insulin analogs or potentiating endogenous insulin signaling. Besides recent developments in GLP-1 receptor agonists and Sodium-Glucose Cotransporter-2 (SGLT2) inhibitors, existing therapies either require injections or have negative side effects such as weight gain. Progress has been made against obesity, but approved therapeutics only focus on hunger repression or inhibiting the dietary uptake of lipids. While the potential approval of a Thyroid Receptor Beta (THβ) agonist such as resmetirom (Madrigal Pharmaceuticals) may offer promise as a first NAFLD/NASH drug, there are currently no FDA-approved drugs for these conditions.^716^ ASCVD lacks a cure, relying on slowing the rate of the inevitable by pharmacologically lowering LDL cholesterol. In the realm of IBD, there is no curative treatment, only the usage of aminosalicylate, corticosteroid, or biologic therapy to reduce intestinal inflammation. Hepatocellular carcinoma chemotherapy resistance employs small molecule antagonists against FGFR4, but most of these approaches are strongly impacted by patient-specific mutations.

Contrasting with the status-quo, BA-centric approaches offer some distinct advantages over the traditional approaches. BA therapeutics offer significant benefits for T2DM, obesity, and NAFLD/NASH treatment relative to existing therapeutics, inhibiting ceramide synthesis, strongly modulating hepatic metabolism, and mirroring the existing therapeutic viability of GLP-1 receptor pharmacology. While BA therapeutics may not surpass the immense benefit of statin therapy, their ability to prevent ceramide synthesis is likely to strongly impact the inflammatory aspects of ASCVD disease progression. IBD management with BA therapeutics has a high benefit compared to the traditional approach directly targeting the Th17 and Treg centric pathway that drives disease pathogenesis. Lastly, a more advanced BA therapeutic approach such as PROTAC development may provide more profound FGFR4 suppression, better outcomes, and a lower likelihood for patient-specific resistance.

### Navigating preclinical to clinical translational challenges

BA research encounters many challenges that prevent the direct translation of preclinical findings into clinical applications. There are four major translational challenges seen between preclinical research and clinical outcomes that must be minimized to ensure translational success. The first challenge arises from the substantial differences in lipid metabolism between rodents and humans, potentially biasing both cholesterol availability for BA synthesis and interpretability of lipid-centric outcomes. Rodents, unlike humans, predominantly store cholesterol within HDL, intrinsically having a lower risk for ASCVD before humanization techniques are applied.^717^ Addressing this, known solutions involve the development of rodent genetic variants or human hepatocyte xenotransplantation models that express lipid profiles very similar to that of humans.

Similarly, the second translational challenge stems from species-specific differences between the BA pools of rodents and humans. For example, rodents produce large quantities of FXR-antagonistic MCAs while humans do not. Although currently unresolved, genetic ablation of *CYP2C70* with or without *CYP2A12* has been found to produce a BA pool more akin to that of humans.^22,25,718–720^ Further genetic and biochemical research is crucial for refining the BA pools of rodents to more similarly match that of humans.

The third translational challenge revolves around species-specific differences in the coding sequence of BA receptors. Beyond the impacts of varying the composition of a single BA within the BA pool, small differences in the coding sequence of BA receptors strongly modulate the potency and efficacy of all known receptor agonists/antagonists. A prime example of such are MCBAs, whose modulatory activity on canonical BA scaffold receptor pharmacology in some cases is completely different species-to-species. To further highlight the issue, many reports have shown that species-specific differences to the BA receptor coding sequence can cause considerable shifts up to a half-order in magnitude in assay EC_50_/IC_50_ results when comparing human and rodent models.^654,721^ To address this challenge, the most straightforward solution may be to humanize rodent BA receptor coding sequences to match those of humans.

The final translational challenge in BA research relates to intrinsic species-specific differences in signaling cascades, in which post-translational protein modifications, transcription factor cis-elements, and their biological outcomes may differ from species to species.^722^ While altering the substrate selectivity and enzymatic activity of an entire signaling cascade proves challenging due to the multitude of targets, adding, removing, or changing the identity of cis-element sequences emerges as a viable option.

### Future directions for BA-centric research

BA-centric research has developed tremendously since its inception, but as more questions are answered many new questions are asked. From the perspective of biochemistry many questions need to be answered pertaining to BA synthesis and maturation. It is crucial to reveal what factors drive BA synthesis between the classical and alternative pathway of synthesis, highlighted in importance by the abnormal 12α-OH BA levels seen in T2DM, obese, and NAFLD/NASH patients. From such, more research needs to be done on the mechanisms connecting TGR5 and CYP8B1, especially how this may be modulated during insulin resistance.

From the perspective of physiology, perhaps the most imperative question to further investigate is the effect of gut FXR-induced ceramides on disease pathogenesis. Alongside such, more work is needed to pinpoint human organ-specific BA pool compositions, which in tandem with a better understanding of the gut/liver FGF15/19-[FGFR4/βK]-FXR cascades would prove incredibly important. The impacts of FXR and TGR5 within mucosal immunity also warrant further investigation, in which there needs to be a better consensus on how these two signaling pathways regulate Treg and Th17 differentiation. The discovery of non-canonical BA receptors opens many questions, importantly opening the door for new modulatory points within the FXR/TGR5 signaling cascades and BA physiology as a whole. Lastly, more research is needed to investigate the physiological purpose and functions of MCBAs.

BA pharmacology is evolving rapidly. Gut restricted receptor modulators allow for potent pharmacological effects without systemic side effects that almost discarded the therapeutic field as a whole. Further research is needed to develop additional ways to circumvent the traditional pitfalls of BA receptor modulators. SFXRMs should be thoroughly investigated, alongside new design strategies for gut-restriction, non-steroidal scaffolds, tissue-specific formulations, and strongly said it is imperative to see if the potent preclinical effects of gut-restricted FXR agonists/antagonists and TGR5 agonists translate to humans.

### Don’t give up on BAs

If developed correctly, systemic and gut-restricted FXR/TGR5 modulators, upstream regulators of BA signaling cascades, proteins that control BA pool size/composition, and microbiome-targeted therapies provide very attractive pharmacological targets for drug discovery against cardiometabolic, inflammatory, and neoplastic diseases. BAs are widespread physiological regulators, modulating carbohydrate, lipid, protein, inflammatory, and basal metabolic pathways. Abnormal BA pool size and/or composition can physiologically predispose for cardiometabolic disease, notably by upregulating the synthesis of ceramides. Abnormal BA pool size or composition additionally influences the ensemble biochemical properties of the BA pool, such as surfactant efficacy and relative agonism between FXR/TGR5.

**Fig. 18:**
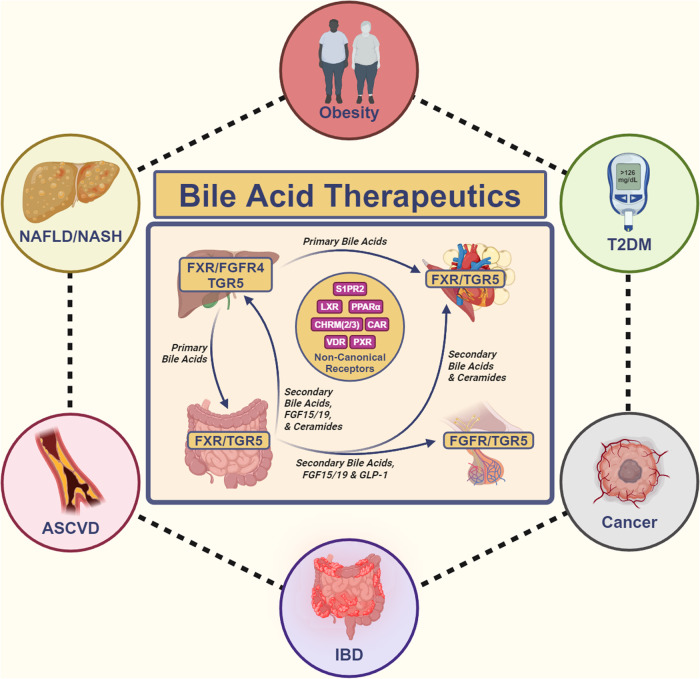
Summary—BA signaling in health and disease. BAs and their associated signaling pathways are central regulators of health. Their biochemistry, physiology, and pharmacology can protect against or if aberrant induce the pathogenesis of disease. BA therapeutics offer immense clinical potential, which if further developed may provide novel treatments for multiple high morbidity/mortality conditions. This figure was created with BioRender.com

There is a dire need for new innovations in drug discovery to more effectively treat cardiometabolic, inflammatory, and neoplastic diseases. In this review, we provided a bottom-up approach on BAs, explaining their biochemistry, physiology, and pharmacology at canonical and non-canonical receptors (Fig. 18). Using such, we thoroughly dissected how abnormal BA physiology directly induces disease pathogenesis and in the case of cardiometabolic disease is highly driven by gut-synthesized ceramides. Lastly, we rationalized novel targets for further translational drug discovery and provided future perspectives. Cardiometabolic, inflammatory, and neoplastic diseases are hard to drug due to complex biochemistry, the lack of deterministic models for disease pathogenesis, and have no well-defined curative treatments, leaving a large gap in the market for new therapies to ease the burden and treat unmet needs. BAs and their associated signaling cascades are crucial for proper health and homeostasis. Although BA therapeutics had a tumultuous start, they have now been refined to reveal a high therapeutic potential, simply said, don’t give up on BAs.
